# Commonalities and differences in set-up and data collection across European spondyloarthritis registries — results from the EuroSpA collaboration

**DOI:** 10.1186/s13075-023-03184-7

**Published:** 2023-10-19

**Authors:** Louise Linde, Lykke M. Ørnbjerg, Simon H. Rasmussen, Thorvardur Jon Love, Anne Gitte Loft, Jakub Závada, Jiří Vencovský, Karin Laas, Dan Nordstrom, Tuulikki Sokka-Isler, Bjorn Gudbjornsson, Gerdur Gröndal, Florenzo Iannone, Roberta Ramonda, Pasoon Hellamand, Eirik K. Kristianslund, Tore K. Kvien, Ana M. Rodrigues, Maria J. Santos, Catalin Codreanu, Ziga Rotar, Matija Tomšič, Isabel Castrejon, Federico Díaz-Gonzáles, Daniela Di Giuseppe, Lotta Ljung, Michael J. Nissen, Adrian Ciurea, Gary J. Macfarlane, Maureen Heddle, Bente Glintborg, Mikkel Østergaard, Merete L. Hetland

**Affiliations:** 1 Copenhagen Center for Arthritis Research (COPECARE), Rigshospitalet, Glostrup, Denmark; 2 Faculty of Medicine, University of Iceland, Reykjavik, Iceland; 3 Department of Rheumatology, Aarhus University Hospital, Aarhus, Denmark; 4 Department of Rheumatology, 1st Faculty of Medicine, Charles University, Prague, Czech Republic; 5 Department of Rheumatology, East-Tallinn Central Hospital, Tallinn, Estonia; 6 Departments of Medicine and Rheumatology, Helsinki University Hospital, Helsinki, Finland; 7 Faculty of Health Sciences, University of Eastern Finland, Jyvaskyla, Finland; 8 Rheumatology Unit, University of Bari, Bari, Italy; 9 Rheumatology Unit, Department of Medicine (DIMED), University of Padova, Padova, Italy; 10 Department of Clinical Immunology and Rheumatology, Amsterdam Medical Center, Amsterdam, Netherlands; 11 Center for Treatment of Rheumatic and Musculoskeletal Diseases (REMEDY), Diakonhjemmet Hospital, Oslo, Norway; 12 Sociedade Portuguesa de Reumatologia, Reuma.pt, Lisbon, Portugal; 13 Department of Rheumatology, Hospital Garcia de Orta, Almada, Lisbon, Portugal; 14 Center for Rheumatic Diseases, University of Medicine and Pharmacy, Bucharest, Romania; 15 Department of Rheumatology, University Medical Centre Ljubljana, Ljubljana, Slovenia; 16 Department of Rheumatology, Hospital General Universitario Gregorio Marañón, Madrid, Spain; 17 Universidad de La Laguna and Rheumatology Service, La Laguna, Spain; 18 Clinical Epidemiology Division, Department of Medicine Solna, Karolinska Institutet, Stockholm, Sweden; 19 Department of Rheumatology, Geneva University Hospital, Geneva, Switzerland; 20 Department of Rheumatology, University Hospital Zurich, University of Zurich, Zurich, Switzerland; 21 Aberdeen Centre for Arthritis and Musculoskeletal Health (Epidemiology Group), University of Aberdeen, Aberdeen, UK; 22 Center for Rheumatology and Spine Diseases, DANBIO Registry, Rigshospitalet, Glostrup, Denmark

**Keywords:** Spondyloarthritis, European registries, Clinical data collection, Collaborative research, Real-world evidence

## Abstract

**Background:**

In European axial spondyloarthritis (axSpA) and psoriatic arthritis (PsA) clinical registries, we aimed to investigate commonalities and differences in (1) set-up, clinical data collection; (2) data availability and completeness; and (3) wording, recall period, and scale used for selected patient-reported outcome measures (PROMs).

**Methods:**

Data was obtained as part of the EuroSpA Research Collaboration Network and consisted of (1) an online survey and follow-up interview, (2) upload of real-world data, and (3) selected PROMs included in the online survey.

**Results:**

Fifteen registries participated, contributing 33,948 patients (axSpA: 21,330 (63%), PsA: 12,618 (37%)). The reported coverage of eligible patients ranged from 0.5 to 100%. Information on age, sex, biological/targeted synthetic disease-modifying anti-rheumatic drug treatment, disease duration, and C-reactive protein was available in all registries with data completeness between 85% and 100%. All PROMs (Bath Ankylosing Spondylitis Disease Activity and Functional Indices, Health Assessment Questionnaire, and patient global, pain and fatigue assessments) were more complete after 2015 (68–86%) compared to prior (50–79%). Patient global, pain and fatigue assessments showed heterogeneity between registries in terms of wording, recall periods, and scale.

**Conclusion:**

Important heterogeneity in registry design and data collection across fifteen European axSpA and PsA registries was observed. Several core measures were widely available, and an increase in data completeness of PROMs in recent years was identified. This study might serve as a basis for examining how differences in data collection across registries may impact the results of collaborative research in the future.

**Supplementary Information:**

The online version contains supplementary material available at 10.1186/s13075-023-03184-7.

## Background

Clinical registries and observational cohorts are essential for studying disease course, treatment effect, and safety in real-world patients. To study rare exposures and outcomes, very large study populations are required, such as through collaborative research across countries. Many countries have established clinical rheumatology registries [1–13]; however, differences in their design, data availability, and completeness pose a challenge when researchers pool data from multiple countries [14, 15].

In rheumatoid arthritis (RA), two surveys conducted among 25 European clinical cohorts and registries, and 14 biological disease-modifying anti-rheumatic drugs (bDMARD) registries under the European Alliance of Associations for Rheumatology (EULAR), suggested that existing heterogeneity in the data collection represents a limitation for data merging and collaborative research. As an example, the registries used diverse methods and instruments for measuring patient-reported outcomes, hampering direct comparability and interpretation [16–19].

The EuroSpA Research Collaboration Network (RCN) is a scientific collaboration among European clinical registries, collecting information on patients with spondyloarthritis (SpA), including axial SpA (axSpA) and psoriatic arthritis (PsA). The individual registries collect a broad range of clinical data relevant for the everyday management of patients with SpA (www.eurospa.eu). However, specific knowledge about the commonalities and differences in data collection across the 16 participating registries is limited. The experience from RA clinical registries [16, 17] prompted the need for a similar cross-country exploration of data collection practices in SpA to gain a better understanding of the data used in pooled analyses. Ultimately, such knowledge may guide the design and interpretation of future collaborative studies. Furthermore, as recently suggested in the European Medicines Agency Patients Registries Initiative [20], it would be beneficial for collaborative research if a set of commonly collected variables with high data availability were defined.

The objective of this study was therefore to explore the design of European registries collecting information on axSpA and PsA, including the commonalities and differences in (1) the set-up, clinical data collection, and funding; (2) data availability and completeness; and (3) the wording, recall period, and scale of patient-reported outcome measures (PROMs).

## Methods

The study consisted of three parts: (1) an online survey designed to capture aspects of registry set-up and clinical data collection, (2) data availability and completeness analysis performed on real-world data collected through EuroSpA, and (3) investigation of the wording, recall period and scale used for selected patient-reported outcome measures (PROMs).

### Online survey regarding registry design

The survey data were collected and managed using the Research Electronic Data Capture (REDCap) tool, a secure, web-based software platform designed to support data capture for research studies [21, 22]. The survey covered the following 12 themes: general registry information (e.g., set-up, infrastructure for data-collection, funding), data management, demographics, diagnosis, disease characteristics, medication, safety, PROMs, lifestyle, laboratory measures, imaging, and comorbidities. The number of individual questions covered in each theme varied from 9 (safety) to 56 (general registry information), the full survey is included as Supplementary material. Each registry assigned 1–3 persons with a thorough knowledge of the registry, hereafter called “registry experts,” to complete the survey. Two investigators (LL, LØ) then reviewed the responses for inconsistencies and missingness. Next, a one-hour semi-structured interview was conducted through a video link by the same two investigators to supplement and validate the survey responses. A common interview guide was shared with the registry experts ahead of the interview (see Supplementary material).

### Patient data availability and completeness assessment of uploaded datasets

Considering the themes explored in the online survey, data availability across registries and data completeness across variables were investigated. A variable was considered available if collected in the registry; the data completeness was reported for each available variable. We used patient data that had been prospectively collected in the registries and uploaded onto a secure server by the individual registries for secondary use in the EuroSpA collaboration. Data were pseudonymized, i.e., personal identifiers had been removed and replaced with placeholder values prior to upload. Previous EuroSpA studies have been based on data uploaded in a similar manner [23, 24]. For the current study, we included data on patients with a clinical diagnosis of axSpA or PsA, aged 18 years or older, and followed in one of the participating registries from the start of their first course of biological (b) DMARD or targeted synthetic (ts) DMARD therapy between 2000 to 2021. Data from the baseline visit of the first b/tsDMARD treatment course were used for this study. A baseline visit was defined as a visit from 4 weeks before to 4 weeks after the treatment initiation date, with priority given to the closest visit before treatment start. Baseline visit data included age, time since diagnosis, clinical disease characteristics, medication, PROMs, and inflammatory markers. Other variables, e.g., HLA-B27, lifestyle, comorbidities, and classification criteria were considered patient-specific and were included independently of the baseline visit, if available in the registry. The availability of variables not accessible for evaluation in the uploaded data was instead based on the survey responses provided by the registry experts.

### Wording, recall period, and scale used for selected patient-reported outcome measures (PROMs)

In the online survey, the registry experts reported the specific wording (translated into English when necessary), recall period, and scales (NRS or VAS) used in the patient global, pain and fatigue assessments. Further details were explored during the follow-up interview, and furthermore, the reported scale was verified by visual inspection of the distribution of the patient scores in the uploaded data.

## Results

Registries from 15 countries participated: ATTRA (Czech Republic), DANBIO (Denmark), ERSBTR (Estonia), ROB-FIN (Finland), ICEBIO (Iceland), GISEA (Italy), AmSpA (Netherlands), NOR-DMARD (Norway), Reuma.pt (Portugal), RRBR (Romania), biorx.si (Slovenia), BIOBADASER (Spain), SRQ (Sweden), SCQM (Switzerland), and BSRBR-AS (UK). BSRBR-AS and AmSpA collected data on axSpA only. Data availability and completeness were assessed in a total of 33,948 patients (axSpA: 21,330, PsA: 12,618).

### Online survey regarding registry design

In Table 1, an overview of the 15 registries, based on the online survey and follow-up interviews, is presented. The full survey is included as Supplementary material. A diagnosis was registered using the International Classification of Diseases – tenth revision (ICD-10) in 5 registries, classification criteria in 2 registries, and expert opinion in 1 registry. In the remaining 7 registries all three methods could be applied (Table 1). Treatment with b/tsDMARDs was registered by all, while treatments with conventional synthetic (cs) DMARDs, non-steroidal anti-inflammatory drugs (NSAIDs), and glucocorticoids were registered in 14, 8, and 11 registries, respectively (Table 1). The estimated coverage of eligible patients ranged from 0.5% (Netherlands) to 100% (Romania) for both diagnoses (Table 1). The sources of funding for the registry activities differed, 7/14 from research grants (covering 2–80% of expenses/cost), 4/14 from the public sector (covering 10–100%), 12/14 from industry (20–100%) and other sources in 3/14 registries (10–100%) (Table 1). The funding was further explored during the follow-up interviews and covered expenditures related to the development and running of IT platforms, dedicated research nurses, secretaries, data managers, and statisticians.

**Table 1:** Set-up of 15 registries in EuroSpA

**Country**	**Czechia**	**Denmark**	**Estonia**	**Finland**	**Iceland**	**Italy**	**Netherlands**	**Norway**	**Portugal**	**Romania**	**Slovenia**	**Spain**	**Sweden**	**Switzerland**	**UK**
**Registry**	**ATTRA**	**DANBIO**	**ESRBTR**	**ROB-FIN**	**ICEBIO**	**GISEA**	**AmSpA**	**NOR-DMARD**	**Reuma.pt**	**RRBR**	**Biorx.si**	**BIOBADASER**	**SRQ**	**SCQM**	**BSRBR-AS**
**General information**															
*Patients included in the registry*															
AxSpA/PsA patients in the data completeness analyses (*n*)^a^	3512/1331	4855/3652	<100/<100	1699/720	386/424	171/457	<100/-	1805/1052	1985/1094	1325/242	616/448	1126/1036	791/1084^b^	1893/1021	1091/-
AxSpA, year of start	2002	2002	2013	2000	2008	2010	2019	2000	2009	2013	2010	2000	1999	2005	2012
PsA, year of start	2002	2002	2013	2000	2008	2010	-	2000	2009	2013	2010	2000	1999	2006	-
Other rheumatological conditions	√	√	√	√	√	√		√	√	√	√	√	√	√	
*Additional entry criteria*															
Treatment with a b/ts DMARD	√		√		√	√		√		√	√	√			
*Basis for diagnosis registration*															
ICD-10		√	√	√	√	√		√					√		
Expert opinion	√	√					√		√	√		√		√	
ASAS/mNY criteria	√	√			√		√		√	√	√			√	√
CASPAR criteria	√	√			√		-		√	√	√			√	-
*Number of visits per year*															
First year	3	3	2	2-3	2	2	2	4	3	2	4	1	2	2	3
Subsequent years	2	2	2	1	1	2	2	2	3	2	2	1	2	2	1
**Data entry**															
Electronic	√	√	√	√	√	√	√	√	√	√	√	√	√	√	√
Paper-based	√				√	√	√			√		√		√	√
Interactive fields		√	√				√	√	√	√	√	√	√	√	√
**Medication**															
b/tsDMARDs	√	√	√	√	√	√	√	√	√	√	√	√	√	√	√
csDMARDs	√	√	√	√	√	√		√	√	√	√	√	√	√	√
NSAIDs	√	√					√	√	√				√	√	√
Oral glucocorticoids	√	√	√	√	√	√		√	√	√	√			√	
**Coverage**															
*Geographic area covered by the registry*															
Nationwide	√	√	√		√				√	√	√	√	√	√	√
*Estimate of eligible patients included*															
axSpA	95	95	95	60	95	15	0.5	25	10	100	70	5	82	10	0.5
PsA	95	85	95	60	95	10	-	25	15	100	60	4	76	10	-
*Institutions collecting patient data*															
Private rheumatology practices	√	√			√				√	√			√	√	
University hospital rheumatology departments	√	√	√	√	√	√	√	√	√	√	√	√	√	√	√
Other hospital rheumatology departments	√	√	√	√	√	√		√	√	√	√		√	√	√
**Funding of registry activities (%)**															
Research grants	5			80				20	15	50	20				2
Public sector		15			100							10	50		
Industry	95	85	100	20				80	85	50	80	90	50	90	98
Other						100^c^	100^d^							10	

*axSpA* Axial spondyloarthritis, *PsA* Psoriatic arthritis, *DMARD* Disease-modifying anti-rheumatic drugs, *ICD-10* International Classification of Diseases – tenth revision, *ASAS* Assessment of Spondyloarthritis International Society, *mNY* Modified New York, *CASPAR* Classification Criteria for Psoriatic Arthritis, *NSAID* Non-steroidal anti-inflammatory drugs, *NA* Not available

^a^Secondary pseudonymized baseline data from initiation of the first biologic (b) or targeted synthetic (ts) disease-modifying anti-rheumatic drug (DMARD) treatment on patients with a clinical diagnosis of axSpA and PsA, 18 years or older, followed in one of the participating registries since the start of their first b/tsDMARD between 2000 to 2021

^b^Sweden has provided data on Secukinumab treated patients only

^c^National society for rheumatology is sponsor

^d^General research funds

### Patient data availability and completeness assessment of uploaded datasets

**Fig. 1:**
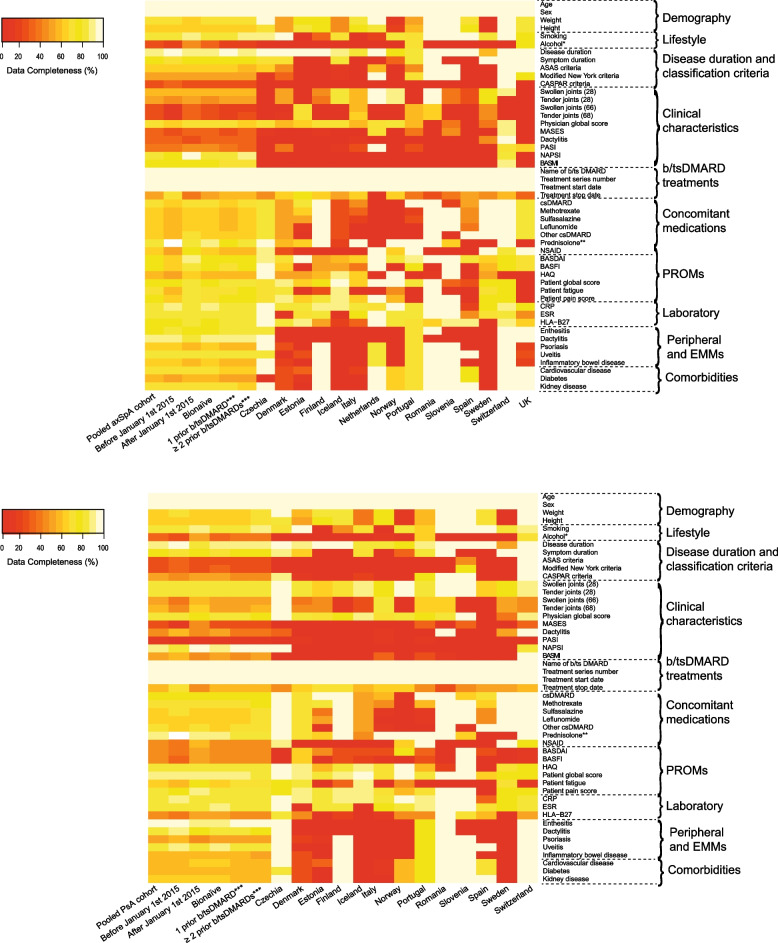
Data completeness for variables collected in axSpA (upper panel) and PsA (lower panel) overall and stratified by time-period for initiation of a b/tsDMARD treatment course, b/tsDMARD history and registry. Legend: Unless otherwise stated, we used secondary pseudonymized baseline data from initiation of the first biologic (b) or targeted synthetic (ts) disease-modifying anti-rheumatic drug (DMARD) treatment on patients with a clinical diagnosis of axial spondyloarthritis (axSpA) and psoriatic arthritis (PsA), 18 years or older, followed in one of the participating registries since the start of their first b/tsDMARD between 2000 and 2021. Sweden has provided data on Secukinumab-treated patients only. ASAS, Assessment of Spondyloarthritis International Society; CASPAR, Classification Criteria for Psoriatic Arthritis; MASES, Maastricht ankylosing spondylitis enthesitis index; PASI, Psoriasis Area and Severity Index; NAPSI, Nail Psoriasis Severity Index; BASMI, Bath Ankylosing Spondylitis Metrology Index; cs, concomitant synthetic; NSAID, non-steroidal anti-inflammatory drug; PROMs, patient-reported outcome measures; BASDAI, Bath Ankylosing Spondylitis Disease Activity Index; BASFI, Bath Ankylosing Spondylitis Functional Index; HAQ, Health Assessment Questionnaire; CRP, C-reactive protein; ESR, erythrocyte sedimentation rate; HLA-B27, Human Leukocyte Antigen subtypes B*2701-2759; EMMs, extra-musculoskeletal manifestations. *Baseline data on patients who initiated a TNFi between January 1, 2009 and December 31, 2018 (alcohol); **baseline data on patients who initiated a new b/tsDMARD from January 1, 2015, and May 31, 2022 (prednisolone); ***baseline data on patients initiating a later line b/tsDMARD (1 prior or ≥2 prior)

In Table 2, data availability and completeness are presented in pooled and stratified data (treatment courses initiated before vs. after January 1, 2015, and axSpA vs PsA), and in Fig. 1 data are further stratified by b/tsDMARD history and registry. Age, sex, disease duration, C-reactive protein (CRP), and details regarding b/tsDMARDs were available in all 15 registries with a data completeness ranging from 85 to 100% (Table 2). Bath Ankylosing Spondylitis Disease Activity Index (BASDAI) scores were also available in all registries; while data completeness varied by the time period (later time period: 71% vs earlier: 54%) and diagnosis (axSpA: 78% vs PsA: 39%) (Table 2). The data completeness in variables describing peripheral involvement, such as swollen/tender joint counts and the Health Assessment Questionnaire (HAQ), were higher in PsA (50–85%) vs. axSpA (16–58%). Conversely, variables designed to evaluate axial involvement, such as the BASDAI, the Bath Ankylosing Spondylitis Functional and Metrology Indices (BASFI and BASMI), had higher data completeness in axSpA (39–78%) vs PsA (7–39%) (Table 2). All PROMs had higher data completeness in the later time period (68–86%) compared to before 2015 (50–79%) (Table 2). Variables describing uveitis and peripheral musculoskeletal manifestations (enthesitis and dactylitis) of SpA were more complete than were comorbid conditions (diabetes, cardiovascular, and kidney disease) (Table 2).

**Table 2:** Results regarding data availability and completeness

**Data source**	**Pooled data from 15 European registries collecting information on patients with SpA**					
	Pooled data (*n*=33,948)		Before January 1, 2015 (*n*=16,207)	From January 1, 2015 (*n*=21,423)	axSpA (*n*=21,330)	PsA (*n*=12,618)
Variables	No of registries with available data	Data completeness, mean % (range)^a^	Data completeness, mean %^a^			
**Demography**						
Age	15	100 (100–100)	100%	100%	100%	100%
Sex	15	100 (100–100)	100%	100%	100%	100%
Weight	14	67 (7–100)	71%	64%	68%	65%
Height	14	64 (13–100)	65%	64%	65%	63%
**Lifestyle**						
Smoking	13	85 (15–100)	82%	88%	85%	84%
Alcohol consumption^b^	4	29 (7–76)	21%	44%	26%	32%
**Disease duration and classification criteria**						
Disease duration (years)	15	92 (53–100)	96%	88%	93%	90%
Symptom duration (years)	9	75 (33–100)	72%	78%	75%	74%
ASAS criteria	9	46 (5–100)	47%	46%	63%	16%
Modified New York criteria	9	38 (5–100)	40%	35%	49%	17%
CASPAR criteria	7	27 (6–100)	29%	26%	15%	48%
**Clinical characteristics at baseline**						
Swollen joint count (28)	14	60 (28–100)	59%	61%	45%	85%
Tender joint count (28)	14	56 (28–100)	53%	59%	38%	85%
Swollen joint count (66)	10	29 (5–74)	20%	37%	16%	50%
Tender joint count (68)	10	31 (6–76)	21%	39%	17%	54%
Physician global	13	71 (13–92)	71%	71%	64%	82%
Enthesitis (MASES)	6	25 (6–70)	20%	29%	29%	16%
Dactylitis (yes/no)	5	33 (10–97)	40%	28%	26%	46%
Skin (PASI binary)	4	40 (1–92)	53%	31%	35%	49%
Nails (NAPSI binary)	2	44 (27–83)	44%	44%	23%	92%
BASMI	8	26 (3–100)	24%	27%	39%	7%
**Biological or targeted synthetic DMARD treatment**						
Name of b/tsDMARD	15	100 (100–100)	100%	100%	100%	100%
Treatment series number	15	100 (100–100)	100%	100%	100%	100%
Treatment start date	15	100 (100–100)	100%	100%	100%	100%
Treatment stop date	15	53 (5–71)	69%	39%	51%	56%
**Concomitant medication at baseline**						
Conventional synthetic (cs) DMARD	14	71 (2–100)	67%	74%	68%	75%
Methotrexate	14	66 (2–100)	64%	68%	63%	71%
Sulfasalazine	14	63 (2–100)	62%	65%	63%	65%
Leflunomide	14	62 (2–100)	60%	63%	60%	64%
Other csDMARDs	13	65 (2–100)	60%	68%	63%	67%
Oral glucocorticoids^c^	11	86 (33–100)	-	86%	84%	88%
NSAIDs	8	56 (16–100)	42%	69%	61%	46%
**Patient-reported outcomes at baseline**						
BASDAI	15	63 (28–100)	54%	71%	78%	39%
BASFI	11	59 (16–100)	50%	68%	74%	35%
HAQ	12	68 (14–97)	63%	72%	58%	83%
Patient global	14	82 (43–100)	79%	85%	79%	87%
Patient fatigue	8	68 (23–90)	57%	79%	71%	64%
Patient pain	13	77 (26–100)	68%	86%	74%	82%
**Laboratory parameters at baseline**						
CRP	15	85 (22–100)	88%	83%	85%	85%
ESR	13	84 (46–100)	85%	82%	83%	85%
HLA-B27	14	67 (8–95)	63%	71%	80%	46%
**Peripheral and extra-musculoskeletal manifestations of spondyloarthritis (ever/never)**						
Enthesitis	5	78 (73–100)	82%	75%	80%	73%
Dactylitis	6	80 (4–100)	91%	72%	79%	81%
Psoriasis	12	56 (2–100)	61%	53%	60%	50%
Uveitis	11	84 (4–100)	87%	82%	83%	86%
Inflammatory bowel disease	11	57 (1–100)	61%	53%	60%	51%
**Comorbidities (ever/never)**						
Cardiovascular	13	65 (10–100)	63%	68%	69%	59%
Diabetes	13	55 (7–100)	53%	57%	54%	57%
Kidney disease	12	66 (3–100)	65%	67%	69%	60%

Unless otherwise stated, we used secondary pseudonymized baseline data from initiation of the first biologic (b) or targeted synthetic (ts) disease-modifying anti-rheumatic drug (DMARD) treatment on patients with a clinical diagnosis of axial spondyloarthritis (axSpA) and psoriatic arthritis (PsA), 18 years or older, followed in one of the participating registries since the start of their first b/tsDMARD between 2000 to 2021. Sweden has provided data on Secukinumab-treated patients only

*ASAS* Assessment of Spondyloarthritis International Society, *CASPAR* Classification Criteria for Psoriatic Arthritis, *MASES* Maastricht Ankylosing Spondylitis Enthesitis Index, *PASI* Psoriasis Area and Severity Index, *NAPSI* Nail Psoriasis Severity Index, *BASMI* Bath Ankylosing Spondylitis Metrology Index, *NSAID* non-steroidal anti-inflammatory drug, *BASDAI* Bath Ankylosing Spondylitis Disease Activity Index, *BASFI* Bath Ankylosing Spondylitis Functional Index, *HAQ* Health Assessment Questionnaire, *CRP* C-reactive protein, *ESR* Erythrocyte sedimentation rate, *HLA-B27* Human Leukocyte Antigen subtypes B*2701-2759

^a^Among registries with available data on the variable

^b^Data based on patients who initiated a TNFi between January 1, 2009, and December 31, 2018

^c^Data based on patients who initiated a new b/tsDMARD from January 1, 2015, and May 31, 2022

### Variables not available in the uploaded data

Additional variables, such as physical activity, intramuscular and intra-articular use of glucocorticoids, EuroQol-5 Dimensions (EQ-5D), other comorbid conditions, imaging, and adverse events were available in some registries, as reported by the registry experts (Supplementary Table S1). Data completeness for these variables was not available in this study.

### Wording, recall period, and scale used for selected patient-reported outcome measures (PROMs)

An overview of selected PROMs used in axSpA across registries is presented in Table 3 and a similar overview for PsA in Supplementary Table S2. For both diagnoses, differences in the wording, recall period, and scale were observed. For patient global, the questions referred to either “overall impact due to disease activity” or “overall impact due to the rheumatic disease”. For patient pain, the questions referred to either “pain due to the rheumatic disease,” “spinal pain,” or pain non-specifically. For patient fatigue, the questions referred to either “unusual fatigue/tiredness,” “fatigue due to the disease,” or to fatigue non-specifically. For both patient global, pain and fatigue assessments, the recall periods varied from “at the moment” to “last week,” and the assessments were performed using either numeric rating scales (NRS) from 0 to 10 or 100 or visual analog scales (VAS). The BASDAI and BASFI were assessed using either NRS 0–10 or 100 mm/10 cm VAS.

**Table 3:** Overview of selected patient-reported outcome measures in axSpA across registries

Registry	Patient global assessment		Patient pain assessment		Patient fatigue assessment		BASDAI/BASFI	
	Wording^a^	Scale^b^	Wording^a^	Scale^b^	Wording^a^	Scale^b^	Registered	Scale^b^
ATTRA (Czechia)	Please indicate below how you feel when you consider all the ways in which your illness now affects you	VAS and NRS (0–100)	How much pain has your illness caused you DURING THE PAST WEEK?	NRS (0–100)	How much of a problem has unsual fatigue been for you DURING THE PAST WEEK?	NRS (0–100)	Yes/yes [25]	VAS
DANBIO (Denmark)	How does the arthritis affect your overall life at the moment?	VAS	How much pain due to arthritis do you have at the moment?	VAS	How tired are you at the moment?	VAS	Yes/yes [26]	VAS
ESRBTR (Estonia)	Patient´s evaluation of disease activity on a VAS ranging from “no activity” to “very active”.	VAS	Patient´s evaluation of pain on a VAS ranging from “no pain” to “strong pain”.	VAS	-	-	Yes/no	NRS
ROBFIN (Finland)	How is your health condition today? VAS ranging from “very good to “worst possible”	VAS	How much pain have you been experiencing during last week?	VAS	How much fatigue have you been experiencing during last week?	VAS	Yes/yes [27]	VAS
ICEBIO (Iceland)	Put a mark on the line below which illustrates the disease activity on your health due to your disease during the last week	VAS	Put a mark on the line below which illustrates the pain due to your disease during the last week	VAS	Put a mark on the line below which illustrates the fatigue due to your disease during the last week	VAS	Yes/yes	VAS
GISEA (Italy)	Considering all the ways your arthritis has affected you, how active do you feel your arthritis is today on a scale ranging from 0 to 100?	NRS 0–100	Numerical rating scale ranging from 0 (no pain) to 100 (worst imaginable pain) measuring actual pain intensity during the last 24 hours	NRS 0–100	-	-	Yes/yes	VAS
AmSpA (Netherlands)	How active was your disease the last week?	NRS	How much back pain did you have during the night the last week?	NRS	How tired were you the last week?	-	Yes/yes	VAS
NOR-DMARD (Norway)	We kindly ask you to evaluate the activity in your arthritis during the last week. Considering all the symptoms you have had, how would you evaluate your condition?	VAS	How much pain have you had during the last week?	VAS	To what degree has a feeling of unusual tiredness or exhaustion been a problem for you during the last week?	VAS	Yes/no	VAS
Reuma.pt (Portugal)	Considering the way the disease disturbs you, how did you feel during the last week?	NRS and VAS	Please indicate the level of pain that you felt in your spine at any moment (day or night) during last week	VAS	FACIT questionnaire	-	Yes/yes [28]	VAS
RRBR (Romania)	Please rate how much the disease is globally affecting you, taking into account all the aspects of the disease (e.g., psoriasis and arthritis) over the past week	NRS	^c^	-	^d^	-	Yes/no	VAS
Biorx.si (Slovenia)	How does your disease affect you today?	NRS	How severe was the pain in the past week?	NRS	-	-	Yes/yes	NRS
BIOBADASER (Spain)	No specific wording, depends on each center	NRS	-	-	-	-	Yes/no [29]	VAS
SRQ (Sweden)	How have you felt in the last week, in general, given your rheumatic disease?	VAS	How much pain have you had in the last week due to your rheumatic disease?	VAS	How tired have you been in the last week due to your rheumatic disease?	VAS	Yes/yes [30, 31]	VAS
SCQM (Switzerland)	How active is your disease today?	NRS	How would you rate your overall pain in the past 7 days?	NRS	^d^	-	Yes/yes	VAS
BSRBR-AS (UK)	Bath Ankylosing Spondylitis Global Score (BAS-G)	-	Pain question in SF12, EQ5D, pain 100 mm VAS	-	Chalder Fatigue Scale	-	Yes/yes [32, 33]	VAS

*NRS* Numeric rating scale from 0 to 10 unless stated otherwise, *VAS* Visual analog scale from 0 to 100 mm, *BASDAI* Bath Ankylosing Spondylitis Disease Activity Index, *BASFI* Bath Ankylosing Spondylitis Functional Index

^a^The wording is based on a translation from the original language (if not English) in the online survey and follow-up interviews

^b^Scales are evaluated visually using graphic presentation of the respective patient assessments in secondary anonymized baseline data from initiation of the first biologic (b) or targeted synthetic (ts) disease-modifying anti-rheumatic drug (DMARD) treatment on patients with a clinical diagnosis of axial spondyloarthritis, 18 years or older, followed in one of the participating registries since the start of their first b/tsDMARD between 2000 to 2021. Sweden has provided data on Secukinumab treated patients only

^c^BASDAI pain question

^d^BASDAI fatigue question

## Discussion

In this study, we investigated the design and data collection in 15 European SpA registries, covering ≈34,000 patients with axSpA and PsA. By collecting details of coverage, recruitment, funding, and assessment of PROMs in the participating registries, we have provided insights into potential challenges when attempting to pool data. High data completeness was observed in core demographic, clinical, and treatment-related variables, and moreover, we observed an increased data completeness of PROMs in recent years.

This study is the first to comprehensively characterize the commonalities and differences across European SpA registries. Heterogeneity across registries has been acknowledged as a factor in interpreting pooled data since EuroSpA was established in 2017, and this study provides further insights into such differences [14, 15, 34, 35]. In RA, two collaborative cross-country studies concluded that further collaboration would benefit from harmonization of data collection [16, 17]. Similarities between our study and the RA studies include the survey-based collection of information from registry experts regarding different aspects of European registries. Our study, however, adds further weight by incorporating real-world data uploaded by the registries for assessment of data completeness.

We noted large variation in coverage across registries, some covering up to 100% of eligible patients and others only a small proportion. This implies that some registry cohorts may be generally representative of patients with SpA in that country or region, whereas other cohorts may be highly selected. Such heterogeneity should be considered when pooling data across registries. Another interesting finding was that in some registries, a diagnosis could be assigned using several methods, i.e., either ICD-10, classification criteria, or expert opinion, while in two registries, classification criteria was the only method used. This may reflect that the registries have different main purposes - some of them are primarily clinical while others are mainly used for research. How a diagnosis is established is of importance since the concordance between clinical diagnoses and fulfillment of classification criteria is not complete, and the clinical characteristics of the patients may also differ according to the diagnostic strategy. In a recent study, 83% of patients with a clinical axSpA diagnosis (ICD-10 of all axSpA diagnoses combined) fulfilled either Assessment of SpondyloArthritis international Society (ASAS) or modified New York classification criteria, and those fulfilling the criteria were more often men and HLA-B27 positive but had less enthesitis [36]. To gain more insight, a future perspective would be to investigate how the different registration strategies are balanced in the registries.

We observed similar frequencies of missingness in our data and in the collated estimates previously reported by Radner et al. in European RA registries for disease duration, patient global score, patient pain, HAQ, joint counts and CRP (0–20%) and treatment with NSAID (20–40%), while our data were more complete regarding cigarette smoking and fatigue [16]. However, it should be noted that the frequencies presented by Radner et al. were self-reported estimates, while in this study they were based on calculations of real data [16]. As could be expected, the BASDAI and BASFI, which are measures developed for use in patients with ankylosing spondylitis, had more complete data in axSpA than in PsA patients, probably reflecting that the majority of the latter has a phenotype with predominantly peripheral involvement. It could also suggest that axial PsA is not routinely looked for in the clinical encounter and therefore tools to assess the axial domain of PsA are not applied in a subset of patients. In general, routine registration of PsA patients may be challenged by the heterogeneity of PsA and the large number of potentially affected domains.

Interestingly, we found higher data completeness across all PROMs in the later time period (after 2015), which may be a sign of an increasing focus on patient engagement, as illustrated by implementing online digital solutions to facilitate data collections using touch screens and apps [37–39].

Our evaluation of PROMs across registries revealed differences in the use of wording, recall period, and scale. The differences were most evident for the patient global, pain and fatigue scores, which could reflect that no specific wording, recall, or scale for the assessment of these concepts has been recommended across rheumatic diseases. However, some variation in the use of scale was still observed for the BASDAI and BASFI although these have been validated in several countries [25–33].

Regarding the wording, only rough comparisons should be made due to the probable semantic differences following the translation of the original questions performed by the registry experts. Possible explanations of the differences observed in our study are many, given the heterogeneity of the registries in general. For instance, we could speculate that data collection practices in axSpA and PsA might have been influenced by RA registries since the movement towards including PROMs as outcome measures in rheumatology started with the development of a core set for endpoints in RA [40]. Several years later, recommendations for AS-specific scores and scales for spinal pain, patient global, and fatigue were proposed in the ASAS core set [41, 42].

In line with this theory, we have seen that the majority of the SpA registries included in our study ask about pain in more general terms and not about spinal pain specifically. Conversely, since widespread pain has been shown to be a strong predictor of poor outcome [43], *and* spinal pain is already included in the BASDAI, the registries may also have made an active decision to consider pain more generally. The impact of such cross-registry differences in PROM wording, recall period, and scale on data from pooled analyses has not been investigated.

Some limitations to our study should be noted. First, since the online survey and follow-up interviews were conducted in a small group of experts from each registry, we cannot exclude that the responses might have differed slightly, had other registry experts been assigned the task. This limitation would, however, mainly apply to the areas where we have not presented real data for verification, e.g., in registry set-up (including coverage, funding, and data management), safety, lifestyle, and imaging. Next, since all except one registry (BSRBR-AS in the UK) are non-English, the patient assessments were translated by the registry experts from the original language to English to compare the wording. Such a translation should ideally have been done by a native speaker, who has good knowledge of both languages and then translated back by a similarly knowledgeable bilingual [44]. Furthermore, the study revealed that some key patient variables were collected in all registries, whereas considerable heterogeneity in data availability was observed for other variables. Also, the wording, recall periods, and scales used for patient assessments differed across registries. Finally, we observed variation in data completeness of patient-reported outcomes over time with an increase in recent years, perhaps reflecting a larger emphasis on their relevance.

## Conclusions

This study has uncovered considerable variation in the design of axSpA and PsA registries across fifteen countries in Europe. Moreover, differences in the availability and completeness of data in general, and the wording, recall periods, and scales used for patient assessments contributed to the heterogeneity., This study might serve as a basis for examining how differences in the current data collection across registries impact the pooled analyses, thereby informing the potential need for a more unified strategy in future collaborative research.

### Supplementary Information

**Additional file 1.** Full online survey.

**Additional file 2.** Interview guide – data mapping. **S1.** Data availability as reported by the registries. **S2.** Overview of selected patient reported outcomes in PsA across registries.

**Additional file 3.** Supplementary data: CRediT statement. 

## Data Availability

The data in this article was collected in the individual registries and made available for secondary use through the EuroSpA Research Collaboration Network [https://eurospa.eu/#registries] Relevant patient-level data may be made available on reasonable request to the corresponding author, but will require approval from all contributing registries.

## Abbreviations

axSpA: Axial spondyloarthritis
ASAS: Assessment of SpondyloArthritis international Society
BASDAI: Bath Ankylosing Spondylitis Disease Activity Index
BASFI: Bath Ankylosing Spondylitis Functional Index
BASMI: Bath Ankylosing Spondylitis Metrology Index
bDMARD: Biological disease-modifying anti-rheumatic drugs
cs: Conventional synthetic
EQ-5D: Euro Qol-5 Dimensions
EULAR: European Alliance of Associations for Rheumatology
HAQ: Health Assessment Questionnaire
ICD-10: The International Classification of Diseases – tenth revision
NRS: Numeric rating scale
NSAIDs: Non-steroidal anti-inflammatory drugs
PROM: Patient-reported outcome measure
PsA: Psoriatic arthritis
RA: Rheumatoid arthritis
RCN: Research Collaboration Network
REDCap: Research Electronic Data Capture
SpA: Spondyloarthritis
ts: Targeted synthetic
VAS: Visual analog scale
