# Supplementary material for: Commonalities and differences in set-up and data collection across European spondyloarthritis registries — results from the EuroSpA collaboration
Source: Arthritis Res Ther. 2023 Oct 19;25:205. doi: 10.1186/s13075-023-03184-7 (PMC10585911; doi:10.1186/s13075-023-03184-7)
Supplement: Supplementary file 1 — Additional file 1. Full online survey. [file 13075_2023_3184_MOESM1_ESM.pdf]

Participant information

Record ID	1
Name	Ziga Rotar
Registry	Biorx.si (Slovenia)
E-mail	ziga.rotar@gmail.com
Deltager	⊗ ja

Response was added on 2021-11-12 09:32:35.

General registry information

What is the status of your registry?	⊗ running and including patients

Coverage

Please estimate how many (percentage) of the eligible spondyloarthritis patients in your country, that are registered	70
Please estimate how many (percentage) of the eligible psoriatic arthritis patients in your country, that are registered	60
How did you obtain the coverage estimate above? (Tick all that apply)	☒ other
Please specify	estimation - the registry is mandatory, but the entry is not strictly enforced by the National health insurance, 2. only patients with axSpA and PsA are followed, i.e., the patients with reactive, enteropathic, undifferentiated SpA are not...
Which institutions/organisations can include patients in your registry? (Tick all that apply)	☒ departments of rheumatology at hospitals<br>☒ departments of rheumatology at university hospitals
Please give an estimate of how many departments of rheumatology at hospitals (not including university hospitals) that include patients in your registry	6
Please give an estimate of how many departments of rheumatology at university hospitals that include patients in your registry	2
When is the data registered? (Tick all that apply)	☒ at pre-specified registry visits
What is the schedule for pre-specified registry visits?	0, 12, 24, 36, 48, 72, 96,... q24wks- during the pandemic, the week 36 was often omitted, and after week 48, a visit per year is what we aim for now.
Is it possible to register visits outside of the pre-specified visit schedule, eg. if a patient has a flare?	⊗ yes

Ethics

Is approval from a local ethics committee needed for a study on de-identified data (eg. a EuroSpA upload)?<br>(Tick all that apply)	☒ yes<br>☒ comment
Comment	we have an umbrella ethics committee approval for effectiveness and safety analyses
Do patients need to sign informed consent to be included in your registry?<br>(Tick all that apply)	☒ yes<br>☒ comment
Comment	the patients sign an informed consent and data release form
Are any additional local approvals needed for a study on de-identified data (eg. a EuroSpA upload)?<br>(Tick all that apply)	☒ no

Funding

How is the registry funded?<br>(Tick all that apply)	☒ from research grants<br>☒ industry
Please estimate the percentage of funds coming from research grants	20<br>((0-100%))
Please estimate the percentage of funds coming from industry, eg. pharmaceutical company	80<br>((0-100%))
The percentages add correctly up to a 100%	
What is the basis of participation by a clinic/department/office in the registry?<br>(Tick all that apply)	☒ mandatory
Is the clinic/department/office financially compensated for registration?	⊗ no

Inclusion criteria

What event triggers the inclusion of a patient into the registry?<br>(Tick all that apply)	☒ other
Please specify	first bDMARD/tsDMARD treatment initiation
Which criteria do you base the inclusion on?<br>(Tick all that apply)	☒ diagnosis<br>☒ treatment
Is a minimum age required for inclusion?	☒ yes, 18 years or above

Which diagnoses are included in your registry?<br><br>(Tick all that apply)	☒ ankylosing spondylitis (AS)<br>☒ non-radiographic axial spondyloarthritis (nr-axSpA)<br>☒ psoriatic arthritis (PsA)<br>☒ rheumatoid arthritis
Which year did inclusion of AS patients begin?	2010
Which year did inclusion of nr-axSpA patients begin?	2015
Which year did inclusion of PsA patients begin?	2010
Do patients need to be treated with biological DMARDs (including targeted synthetic DMARDs) to be included in the registry?	⊗ yes
Have the inclusion criteria changed over time?	⊗ no
General comments	the inclusion criteria did not change, but the eligibility criteria for starting bDMARDs did...<br>They are less strict than they used to be.<br>nr-ax-SpA can be identified from the classification criteria

Response was added on 2021-11-12 09:37:19.

**Data management**	
What are the options for data entry?<br>(Tick all that apply)	☒ electronic
Are the data fields in your registry interactive, such that invalid or unprobable data is flagged when entered (edit checks)<br>(Tick all that apply)	☒ yes<br>☒ comment
Since when (year) has the data fields been interactive?	2008
Comment	validation is not perfect
Please describe any other data validation procedures that you may use	we rarely send out discrepancy queries
How does your registry retain data management services?<br>(Tick all that apply)	☒ external company<br>☒ a researcher/administrative personal does data management beside other duties
Which is the background of your data manager(s)?<br>(Tick all that apply)	☒ clinical
Which are the most commonly used data analysis software/programming languages in your organization?<br>(Tick all that apply)	☒ R<br>☒ excel
How is the data stored in the registry?<br>(Tick all that apply)	☒ a relational database framework (like SQL)
Which are the main data formats used for raw data extractions?<br>(Tick all that apply)	☒ r-derived format
Where is your data collection platform hosted?<br>(Tick all that apply)	☒ external company
Who maintains your data collection platform?<br>(Tick all that apply)	☒ external company
How frequently is the database updated with the latest information?<br>(Tick all that apply)	☒ real-time
Is linkage to other databases or registries possible?	⊗ no

General comments	linkage is doable as patients are identified in the database using the health insurance number, but there is no interactive linkage.

Response was added on 2021-11-12 09:39:12.

Demography

Please indicate which of the following variables are collected in your registry
(Tick all that apply)

- ☒ age (year of birth)
- ☒ sex
- ☒ weight
- ☒ height

Time points for registration

	at inclusion in registry	at start/change of treatment	at follow-up visits	other
Weight	☒	☒	☒	☐
Height	☒	☒	☒	☐

Diagnosis

How is a diagnosis registered?
(Tick all that apply)

- ☒ classification criteria
- ☒ other diagnostic categories

Specify which diagnostic categories: RA, PsA, axSpA

Do you register
(Tick all that apply)

- ☒ date for diagnosis

Do you register
(Tick all that apply)

- ☒ not registered

Time points for registration

	at inclusion in registry	at start/change of treatment	at follow-up visits	other
Day/month/year of diagnosis	☒	☐	☐	☐

Which classification criteria are registered?
(Tick all that apply)

- ☒ ASAS
- ☒ New York
- ☒ CASPAR

Time points for registration

	at inclusion in registry	at start/change of treatment	at follow-up visits	other
ASAS	☒	☐	☐	☐
New York	☒	☐	☐	☐
CASPAR	☒	☐	☐	☐

In which patients can you register ASAS? ⊗ both

Do you register individual ASAS classification items? (Tick all that apply) ☒ yes

Do you register individual New York classification items? (Tick all that apply) ☒ yes

Do you register individual CASPAR classification items? (Tick all that apply) ☒ yes

Response was added on 2021-11-12 09:48:38.

Axial spondyloarthritis

Which disease status characteristics can be registered in axSpA patients? (Tick all that apply)	☒ swollen joint count<br>☒ tender joint count<br>☒ enthesitis<br>☒ physician global<br>☒ other
Please specify	patinent global

Time points for registration

	at inclusion in registry	at start/change of treatment	at follow-up visits	other
Swollen joint count	☒	☒	☒	☐
Tender joint count	☒	☒	☒	☐
Enthesitis	☒	☒	☒	☐
Physician global	☒	☒	☒	☐

Swollen joints

How many swollen joint counts can be registered? (Tick all that apply)	☒ 28<br>☒ other
Please specify	+25 ACJ, SCJ, DIP1-5, ankle, MTP1-5
Do you register specific location of swollen joints? (Tick all that apply)	☒ yes

Tender joints

How many tender joint counts can be registered? (Tick all that apply)	☒ other
Please specify	+26 ACJ, SCJ, DIP1-5, ankle, MTP1-5, hip
Do you register specific location of tender joints? (Tick all that apply)	☒ yes

Enthesitis

Do you register specific location of enthesitis?<br>(Tick all that apply)	☒ yes
Which locations are registered?<br>(Tick all that apply)	☒ achilles tendon insertion<br>☒ 1st costochondral joint<br>☒ 7th costochondral joint<br>☒ posterior superior iliac spine<br>☒ anterior superior iliac spine<br>☒ iliac crests<br>☒ 5th lumbar spinous process
Please indicate which specific indices that are used, if applicable	MASES

Physician Global

Please write the wording of the question regarding physician global	Physician global disease activity assessment - grade on a scale 0-10 (0-no activity, 10- high disease activity)<br>(translate into english if possible)

Coxitis

Do you assess coxitis? If yes, please indicate how it is assessed	no

Response was added on 2021-11-12 09:51:43.

Psoriatic arthritis

Which disease status characteristics can be registered in PsA patients? (Tick all that apply)	☒ swollen joint count<br>☒ tender joint count<br>☒ enthesitis<br>☒ physician Global<br>☒ other
Please specify	patient global, BASDAI,BASFI

Time points for registration

	at inclusion in registry	at start/change of treatment	at follow-up visits	other
Swollen joint count	☒	☒	☒	☐
Tender joint count	☒	☒	☒	☐
Enthesitis	☒	☒	☒	☐
Physician Global	☒	☒	☒	☐

Swollen joints

How many swollen joint counts can be registered? (Tick all that apply)	☒ 28<br>☒ Other
Please specify	+25 ACJ, SCJ, DIP1-5, ankle, MTP1-5
Do you register specific location of swollen joints? (Tick all that apply)	☒ yes

Tender joints

How many tender joint counts can be registered? (Tick all that apply)	☒ 28<br>☒ Other
Please specify	+26 ACJ, SCJ, DIP1-5, ankle, MTP1-5, hip

Entesitis

Do you register specific location of enthesitis? (Tick all that apply)	☒ yes
Which locations are registered? (Tick all that apply)	☒ achilles tendon insertion<br>☒ 1st costochondral joint<br>☒ 7th costochondral joint<br>☒ posterior superior iliac spine<br>☒ anterior superior iliac spine<br>☒ iliac crests<br>☒ 5th lumbar spinous process
Please indicate which specific indices that are used, if applicable	MASES

Physician global

Please write the wording of the question regarding physician global	Physician global disease activity (0-10)<br>(Translate into english if possible)

Coxitis

Do you assess coxitis? If yes, please indicate how it is assessed	Ask the patient if he/she has hip (groin) pain

Response was added on 2021-11-12 09:56:56.

Medication

Which therapies are registered in your registry? (Tick all that apply)

- ☒ biological dmards (bDMARDs), including targeted synthetic dmards (JAK)
- ☒ conventional synthetic dmards (csDMARDs)
- ☒ glucocorticoids
- ☒ NSAIDs
- ☒ medication for comorbidity

bDMARDs

Which year did bDMARD registration begin?	2008
Is it mandatory to register bDMARD therapy?	⊗ yes

csDMARDs

Which year did csDMARD registration begin?	2008
Is it mandatory to register csDMARD therapy?	⊗ yes

Glucocorticoids

Which year did glucocorticoid registration begin?	2008
Is it mandatory to register glucocorticoid therapy?	⊗ yes
Is the mode of administration registered? (Tick all that apply)	☒ not registered

NSAIDs

Which year did NSAID registration begin?	2008
Is it mandatory to register NSAID therapy?	⊗ no

Medicaton for comorbidities

What types of medical therapy for comorbidities are registered?	all medications used at the time of visit can be entered

What information regarding ONGOING medications is registered?

	start date	stop date	temporary start and stop dates	discontinuation reasons	dosage	frequency	administration mode
bDMARDs	☒	☒	☒	☒	☒	☒	☒
csDMARDs	☐	☐	☐	☐	☒	☐	☐
glucocorticoids	☐	☐	☐	☐	☒	☐	☐
NSAIDs	☐	☐	☐	☐	☒	☒	☐
medication for comorbidity	☐	☐	☐	☐	☒	☒	☐

Time points for registration (ONGOING medications)

	at inclusion in registry	at start/change of treatment	at follow-up visits	other
bDMARDs	☒	☒	☒	☐
csDMARDs	☒	☒	☒	☐
glucocorticoids	☒	☒	☒	☐
NSAIDs	☒	☒	☒	☐
medication for comorbidity	☒	☒	☒	☐

What information regarding PAST medications is registered at inclusion in registry?

	start date	stop date	temporary start and stop dates	discontinuation reasons	dosage	name of drug
bDMARDs	☒	☒	☒	☒	☒	☒
csDMARDs	☐	☐	☐	☒	☒	☒
glucocorticoids	☐	☐	☐	☐	☒	☐
NSAIDs	☐	☐	☐	☒	☒	☒

Discontinuation reasons

What are the possible reasons for discontinuation of a bDMARD?<br>(Tick all that apply)	☒ lack of efficacy<br>☒ adverse events<br>☒ remission<br>☒ lost to follow-up
Is it possible to register multiple reasons for discontinuation?	⊗ no
Does your registry link to prescription database or other external data sources on prescriptions on a regular basis as input to registry?<br>(Tick all that apply)	☒ no

Response was added on 2021-11-12 10:07:02.

Patient reported outcomes

Which patient reported outcomes (PROs) are registered in axSpA and/or PsA patients? (Tick all that apply)	☒ BASDAI<br>☒ BASFI<br>☒ pain<br>☒ global disease<br>☒ HAQ
Are any of the PROs registered in either axSpA or PsA only?	⊗ yes
Which PROs are registered uniquely in either axSpA or PsA?	BASDAI and BASFI are mandatory only for axSpA

Mode of registration

What are the options for registration of PROs? (Tick all that apply)	☒ paper forms

Time points for registration

	at inclusion in registry	at start/change of treatment	at follow-up visits	other
BASDAI	☒	☒	☒	☐
BASFI	☒	☒	☒	☐
Pain	☒	☒	☒	☐
Global disease	☒	☒	☒	☐
HAQ	☒	☒	☒	☐

BASDAI

Are the individidual BASDAI components registered?	⊗ yes

BASFI

Are the individidual BASFI components registered?	⊗ yes

Pain, fatigue and global assessments

Please write the wording of the question relating to pain	How severe was the pain in the past week? (please translate into english if possible)
Please write the wording of the question relating to global assessment of disease	How does your disease affect you today? (please translate into english if possible)

HAQ

Are the individual HAQ items registered?	⊗ yes
Which HAQ versions may be used in your registry? (Tick all that apply)	☒ improved HAQ

Response was added on 2021-11-12 10:08:21.

Laboratory

Which laboratory test can be registered?
(Tick all that apply)

☒ ESR
☒ CRP
☒ hemoglobin
☒ ALAT
☒ creatinine
☒ HLA-B27

Time points for registration

	at inclusion in registry	at start/change of treatment	at follow-up visits	other
ESR	☒	☒	☒	☐
CRP	☒	☒	☒	☐
Hemoglobin	☒	☒	☒	☐
ALAT	☒	☒	☒	☐
Creatinine	☒	☒	☒	☐

How are laboratory test results registered?
(Tick all that apply)

☒ entered by health care staff

Response was added on 2021-11-12 10:09:48.

Imaging

Which imaging modalities can be registered? (Tick all that apply)	☒ Magnetic Resonance Imaging (MRI)<br>☒ X-ray

Information on each image

	date of examination	full image report	image file	scoring system	+/- progression	other
What information on each MRI exam is registered?	☒	☐	☐	☐	☐	☐
What information on each x-ray exam is registered?	☒	☐	☐	☐	☐	☐

MRI

Which anatomical regions can be registered? (Tick all that apply)	☒ sacroiliac joint MRI

X-ray

Which anatomical regions can be registered? (Tick all that apply)	☒ sacroiliac joint radiographs
General comments	latest imaging can be recorded at inclusion into the registry, there is no follow-up

Response was added on 2021-11-12 10:13:42.

Comorbidities

Which extraarticular manifestations and comorbid conditions are registered?<br>(Tick all that apply)	☒ uveitis<br>☒ psoriasis<br>☒ inflammatory bowel disease (IBD)<br>☒ ischemic heart disease (IHD)<br>☒ cerebrovascular disease (CVD)<br>☒ hypertension<br>☒ diabetes (DM)<br>☒ dyslipidemia<br>☒ osteoporosis<br>☒ chronic kidney insufficiency (CKI)<br>☒ chronic liver disease, eg. hepatitis, cirrhosis<br>☒ solid cancer<br>☒ hematological cancer<br>☒ depression<br>☒ tuberculosis (TB)<br>☒ other
Please specify	thyroid disease, demyelinating disease, epilepsy, asthma, COPD, peptic ulcer disease,

Time points for registration

	at inclusion in registry	at start/change of treatment	at follow-up visits	other
uveitis	☒	☐	☒	☐
psoriasis	☒	☐	☐	☐
IBD	☒	☐	☐	☐
IHD	☒	☐	☐	☐
CVD	☒	☐	☐	☐
hypertension	☒	☐	☐	☐
DM	☒	☐	☐	☐
dyslipidemia	☒	☐	☐	☐
osteoporosis	☒	☐	☐	☐
CKI	☒	☐	☐	☐
chronic liver disease	☒	☐	☐	☐
solid cancer	☒	☐	☐	☐
hematological cancer	☒	☐	☐	☐
depression	☒	☐	☐	☐
TB	☒	☐	☐	☐

other	☒	☐	☐	☐

Mode of registration - how are comorbid conditions registered?

	patient-reported	by health-staff	linkage from other registry
uveitis	☒	☒	☐
psoriasis	☒	☒	☐
IBD	☒	☒	☐
IHD	☒	☒	☐
CVD	☒	☒	☐
hypertension	☒	☒	☐
DM	☒	☒	☐
dyslipidemia	☒	☒	☐
osteoporosis	☒	☒	☐
CKI	☒	☒	☐
chronic liver disease	☒	☒	☐
solid cancer	☒	☒	☐
hematological cancer	☒	☒	☐
depression	☒	☒	☐
TB	☒	☒	☐
other	☒	☒	☐

Do you use ICD-10 codes for registration?	⊗ no
General comments	patient reported comorbidities are checked against available medical records.

Response was added on 2021-11-12 10:15:48.

Lifestyle

Which lifestyle parameters are registered? (Tick all that apply)
☒ smoking

Time points for registration

	at inclusion in registry	at start/change of treatment	at follow-up visits	other
Smoking	☒	☐	☐	☐

Smoking

How is smoking status characterised? (Tick all that apply)
☒ current
☒ former
☒ never

Do you register a start date? (Tick all that apply)
☒ no date is registered

Do you register a stop date for former smokers?
⊗ No

How is average number of smoked cigarettes registered? (Tick all that apply)
☒ number of daily cigarettes

General comments
We record
years of smoking
cigs smoked per day
years abstaining from smoking at inclusion into the registry, so we can deduce the year of starting and stopping in smokers and ex-smokers

Response was added on 2021-11-12 10:17:22.

Safety

Can you register adverse events in your registry? (Tick all that apply)	☒ yes, directly into registry
Is it mandatory to register adverse events through your registry?	⊗ yes
Which adverse events are registered in your registry? (Tick all that apply)	☒ non-serious adverse events<br>☒ serious adverse events?

Information on adverse events

	date of event	MeddRA	ICD10-code	outcome	other
Non serious AE	☒	☐	☐	☒	☒
Serious AE	☒	☐	☐	☒	☒

Please specify (non serious AE)	picked from a list, entered as free text
Please specify (serious AE)	picked from a list, entered as free text

Participant information

Record ID	2
Name	Karin Laas
Registry	ESRBTR (Estonia)
E-mail	karin.laas@itk.ee
Deltager	⊗ ja

Response was added on 2021-11-04 14:55:21.

General registry information

What is the status of your registry?	⨂ running and including patients

Coverage

Please estimate how many (percentage) of the eligible spondyloarthritis patients in your country, that are registered	95
Please estimate how many (percentage) of the eligible psoriatic arthritis patients in your country, that are registered	95
How did you obtain the coverage estimate above? (Tick all that apply)	☒ by prior study of coverage
Which institutions/organisations can include patients in your registry? (Tick all that apply)	☒ departments of rheumatology at hospitals<br>☒ departments of rheumatology at university hospitals
Please give an estimate of how many departments of rheumatology at hospitals (not including university hospitals) that include patients in your registry	3
Please give an estimate of how many departments of rheumatology at university hospitals that include patients in your registry	1
When is the data registered? (Tick all that apply)	☒ at routine visits
Are all routine visits registered in your registry - or only some?	⨂ only some visits

Ethics

Is approval from a local ethics committee needed for a study on de-identified data (eg. a EuroSpA upload)? (Tick all that apply)	☒ no
Do patients need to sign informed consent to be included in your registry? (Tick all that apply)	☒ no
Are any additional local approvals needed for a study on de-identified data (eg. a EuroSpA upload)? (Tick all that apply)	☒ no

Funding

How is the registry funded?<br>(Tick all that apply)	☒ industry
Please estimate the percentage of funds coming from industry, eg. pharmaceutical company	100<br>((0-100%))
The percentages add correctly up to a 100%	
What is the basis of participation by a clinic/department/office in the registry?<br>(Tick all that apply)	☒ mandatory
Is the clinic/department/office financially compensated for registration?	⊗ yes

Inclusion criteria

What event triggers the inclusion of a patient into the registry?<br>(Tick all that apply)	☒ new treatment
Which criteria do you base the inclusion on?<br>(Tick all that apply)	☒ diagnosis<br>☒ treatment
Is a minimum age required for inclusion?	☒ yes, 18 years or above
Which diagnoses are included in your registry?<br><br>(Tick all that apply)	☒ ankylosing spondylitis (AS)<br>☒ non-radiographic axial spondyloarthritis (nr-axSpA)<br>☒ psoriatic arthritis (PsA)<br>☒ rheumatoid arthritis<br>☒ other
Which year did inclusion of AS patients begin?	2013
Which year did inclusion of nr-axSpA patients begin?	2013
Which year did inclusion of PsA patients begin?	2013
Which other diagnoses are included?	Juvenile idiopathic arthritis
Do patients need to be treated with biological DMARDs (including targeted synthetic DMARDs) to be included in the registry?	⊗ yes
Have the inclusion criteria changed over time?	⊗ no

Response was added on 2021-11-08 19:59:33.

Data management

What are the options for data entry?<br>(Tick all that apply)	☒ electronic
Are the data fields in your registry interactive, such that invalid or unprobable data is flagged when entered (edit checks)<br>(Tick all that apply)	☒ yes
Since when (year) has the data fields been interactive?	2013
How does your registry retain data management services?<br>(Tick all that apply)	☒ person employed as data manager
Which is the background of your data manager(s)?<br>(Tick all that apply)	☒ data science/biostatistician/technical
Which are the most commonly used data analysis software/programming languages in your organization?<br>(Tick all that apply)	☒ stata
How is the data stored in the registry?<br>(Tick all that apply)	☒ a relational database framework (like SQL)
Which are the main data formats used for raw data extractions?<br>(Tick all that apply)	☒ simple text files, eg. csv, tsv
Where is your data collection platform hosted?<br>(Tick all that apply)	☒ by a hospital IT-administration
Who maintains your data collection platform?<br>(Tick all that apply)	☒ hospital IT-administrator
How frequently is the database updated with the latest information?<br>(Tick all that apply)	☒ real-time
Is linkage to other databases or registries possible?	⊗ yes
Which registries can be linked to?<br>(Tick all that apply)	☒ mortality registry<br>☒ electronic medical records

Response was added on 2021-11-08 20:01:21.

Demography

Please indicate which of the following variables are collected in your registry
(Tick all that apply)

☒ age (year of birth)
☒ sex
☒ weight
☒ height
☒ death

Time points for registration

	at inclusion in registry	at start/change of treatment	at follow-up visits	other
Weight	☒	☐	☒	☐
Height	☒	☐	☐	☐

How is vital status registered
(Tick all that apply)

☒ by healthstaff, manually

Diagnosis

How is a diagnosis registered?
(Tick all that apply)

☒ through ICD-10 codes

Do you register
(Tick all that apply)

☒ year of diagnosis

Do you register
(Tick all that apply)

☒ not registered

Time points for registration

	at inclusion in registry	at start/change of treatment	at follow-up visits	other
Day/month/year of diagnosis	☒	☐	☐	☐

Which classification criteria are registered?
(Tick all that apply)

☒ ASAS
☒ CASPAR

Time points for registration

	at inclusion in registry	at start/change of treatment	at follow-up visits	other
ASAS	☒	☐	☐	☐
CASPAR	☒	☐	☐	☐

In which patients can you register ASAS?	⊗ AxSpA
Do you register individual ASAS classification items? (Tick all that apply)	☒ no
Do you register individual CASPAR classification items? (Tick all that apply)	☒ no

Response was added on 2021-11-08 20:01:26.

Axial spondyloarthritis

Which disease status characteristics can be registered in axSpA patients? (Tick all that apply)	☒ swollen joint count<br>☒ tender joint count<br>☒ physician global

Time points for registration

	at inclusion in registry	at start/change of treatment	at follow-up visits	other
Swollen joint count	☒	☒	☒	☐
Tender joint count	☒	☒	☒	☐
Physician global	☒	☒	☒	☐

Swollen joints

How many swollen joint counts can be registered? (Tick all that apply)	☒ 66
Do you register specific location of swollen joints? (Tick all that apply)	☒ no, just joint counts

Tender joints

How many tender joint counts can be registered? (Tick all that apply)	☒ 68
Do you register specific location of tender joints? (Tick all that apply)	☒ no, just joint counts

Physician Global

Please write the wording of the question regarding physician global	Physicians assessment of disease activity (translate into english if possible)

Coxitis

Do you assess coxitis? If yes, please indicate how it is assessed	No

Response was added on 2021-11-08 20:01:30.

Psoriatic arthritis

Which disease status characteristics can be registered in PsA patients?
(Tick all that apply)

☒ swollen joint count
☒ tender joint count
☒ physician Global

Time points for registration

	at inclusion in registry	at start/change of treatment	at follow-up visits	other
Swollen joint count	☒	☒	☒	☐
Tender joint count	☒	☒	☒	☐
Physician Global	☒	☒	☒	☐

Swollen joints

How many swollen joint counts can be registered?
(Tick all that apply)

☒ 28
☒ 66

Do you register specific location of swollen joints?
(Tick all that apply)

☒ no, just joint counts

Tender joints

How many tender joint counts can be registered?
(Tick all that apply)

☒ 28
☒ 68

Do you register specific location of tender joints?
(Tick all that apply)

☒ no, just joint counts

Physician global

Please write the wording of the question regarding physician global

Physicians assessment of disease activity
(Translate into english if possible)

Coxitis

Do you assess coxitis? If yes, please indicate how it is assessed

No

Response was added on 2021-11-08 20:01:35.

Medication

Which therapies are registered in your registry? (Tick all that apply)
☒ biological dmards (bDMARDs), including targeted synthetic dmards (JAK)
☒ conventional synthetic dmards (csDMARDs)
☒ glucocorticoids

bDMARDs

Which year did bDMARD registration begin? 2013

Is it mandatory to register bDMARD therapy? ⊗ yes

csDMARDs

Which year did csDMARD registration begin? 2013

Is it mandatory to register csDMARD therapy? ⊗ yes

Glucocorticoids

Which year did glucocorticoid registration begin? 2013

Is it mandatory to register glucocorticoid therapy? ⊗ yes

Is the mode of administration registered? (Tick all that apply)
☒ oral
☒ intraarticular

Are specific locations of injected joints registered? ⊗ no

What information regarding ONGOING medications is registered?

	start date	stop date	temporary start and stop dates	discontinuation reasons	dosage	frequency	administration mode
bDMARDs	☒	☒	☒	☒	☒	☒	☒
csDMARDs	☐	☐	☐	☐	☒	☐	☒
glucocorticoids	☐	☐	☐	☐	☒	☐	☒

Time points for registration (ONGOING medications)

	at inclusion in registry	at start/change of treatment	at follow-up visits	other
bDMARDs	☒	☒	☒	☐
csDMARDs	☒	☒	☒	☐
glucocorticoids	☒	☒	☒	☐

What information regarding PAST medications is registered at inclusion in registry?

	start date	stop date	temporary start and stop dates	discontinuation reasons	dosage	name of drug
bDMARDs	☐	☐	☐	☒	☒	☒
csDMARDs	☐	☐	☐	☒	☒	☐
glucocorticoids	☐	☐	☐	☐	☒	☒

Discontinuation reasons

What are the possible reasons for discontinuation of a bDMARD?
(Tick all that apply)

☒ lack of efficacy
☒ adverse events
☒ remission
☒ pregnancy wish
☒ infection
☒ lost to follow-up
☒ death

Is it possible to register multiple reasons for discontinuation?

⊗ no

Does your registry link to prescription database or other external data sources on prescriptions on a regular basis as input to registry?
(Tick all that apply)

☒ no

Response was added on 2021-11-08 20:01:39.

Patient reported outcomes

Which patient reported outcomes (PROs) are registered in axSpA and/or PsA patients? (Tick all that apply)	☒ BASDAI<br>☒ pain<br>☒ global disease<br>☒ HAQ
Are any of the PROs registered in either axSpA or PsA only?	⊗ no, all PROs are registered in both diagnoses

Mode of registration

What are the options for registration of PROs? (Tick all that apply)	☒ paper forms

Time points for registration

	at inclusion in registry	at start/change of treatment	at follow-up visits	other
BASDAI	☒	☒	☒	☐
Pain	☒	☒	☒	☐
Global disease	☒	☒	☒	☐
HAQ	☒	☐	☒	☐

BASDAI

Are the individidual BASDAI components registered?	⊗ no, only the composite score

Pain, fatigue and global assessments

Please write the wording of the question relating to pain	patients globals assessment of pain<br>(please translate into english if possible)
Please write the wording of the question relating to global assessment of disease	patients global assessment of disease activity<br>(please translate into english if possible)

HAQ

Are the individual HAQ items registered?	⊗ no
Which HAQ versions may be used in your registry? (Tick all that apply)	☒ stanford HAQ DI without adjustment for use of aids and devices

Response was added on 2021-11-08 20:01:43.

Laboratory

Which laboratory test can be registered? (Tick all that apply)	☒ ESR<br>☒ CRP<br>☒ HLA-B27<br>☒ IgM-RF

Time points for registration

	at inclusion in registry	at start/change of treatment	at follow-up visits	other
ESR	☒	☒	☒	☐
CRP	☒	☒	☒	☐

How are laboratory test results registered? (Tick all that apply)	☒ entered by health care staff

Response was added on 2021-11-08 20:01:57.

Comorbidities

Which extraarticular manifestations and comorbid conditions are registered?
(Tick all that apply)

☒ uveitis
☒ psoriasis
☒ inflammatory bowel disease (IBD)
☒ ischemic heart disease (IHD)
☒ cerebrovascular disease (CVD)
☒ hypertension
☒ diabetes (DM)
☒ dyslipidemia
☒ osteoporosis
☒ chronic kidney insufficiency (CKI)
☒ chronic liver disease, eg. hepatitis, cirrhosis
☒ solid cancer
☒ hematological cancer
☒ depression
☒ tuberculosis (TB)
☒ fibromyalgia

Time points for registration

	at inclusion in registry	at start/change of treatment	at follow-up visits	other
uveitis	☒	☐	☐	☐
psoriasis	☒	☐	☐	☐
IBD	☒	☐	☐	☐
IHD	☒	☐	☐	☐
CVD	☒	☐	☐	☐
hypertension	☒	☐	☐	☐
DM	☒	☐	☐	☐
dyslipidemia	☒	☐	☐	☐
osteoporosis	☒	☐	☐	☐
CKI	☒	☐	☐	☐
chronic liver disease	☒	☐	☐	☐
solid cancer	☒	☐	☐	☐
hematological cancer	☒	☐	☐	☐
depression	☒	☐	☐	☐
TB	☒	☒	☒	☐
fibromyalgia	☒	☐	☐	☐

Mode of registration - how are comorbid conditions registered?

	patient-reported	by health-staff	linkage from other registry
uveitis	☐	☒	☐
psoriasis	☐	☒	☐
IBD	☐	☒	☐
IHD	☐	☒	☐
CVD	☐	☒	☐
hypertension	☐	☒	☐
DM	☐	☒	☐
dyslipidemia	☐	☒	☐
osteoporosis	☐	☒	☐
CKI	☐	☒	☐
chronic liver disease	☐	☒	☐
solid cancer	☐	☒	☐
hematological cancer	☐	☒	☐
depression	☐	☒	☐
TB	☐	☒	☐
fibromyalgia	☐	☒	☐

Do you use ICD-10 codes for registration? ⊗ no

Response was added on 2021-11-08 20:02:07.

Safety

Can you register adverse events in your registry? (Tick all that apply)	☒ yes, directly into registry
Is it mandatory to register adverse events through your registry?	⊗ yes
Which adverse events are registered in your registry? (Tick all that apply)	☒ non-serious adverse events<br>☒ serious adverse events?

Information on adverse events

	date of event	MeddRA	ICD10-code	outcome	other
Non serious AE	☒	☐	☐	☐	☒
Serious AE	☒	☐	☐	☒	☒

Please specify (non serious AE)	drop down menu and free text.
Please specify (serious AE)	drop down menu same as for nSAE

Participant information

Record ID	4
Name	Almut Scherer
Registry	SCQM
E-mail	almut.scherer@scqm.ch
Deltager	⊗ ja

Response was added on 2021-12-17 14:06:20.

General registry information	
What is the status of your registry?	⊗ running and including patients

Coverage	
Please estimate how many (percentage) of the eligible spondyloarthritis patients in your country, that are registered	10
Please estimate how many (percentage) of the eligible psoriatic arthritis patients in your country, that are registered	10
How did you obtain the coverage estimate above? (Tick all that apply)	☒ other
Please specify	These are crude estimates. Based on https://www.ncbi.nlm.nih.gov/pmc/articles/PMC4470267/pdf/nihms407401.pdf we assume prevalence of 1%. With a population of 8.7Mio we expect 87'000 cases. We have recorded 5'000 axSpA patients since 2005, which is about 5%. With incidence, our estimates are higher.
Which institutions/organisations can include patients in your registry? (Tick all that apply)	☒ private rheumatology practices<br>☒ departments of rheumatology at hospitals<br>☒ departments of rheumatology at university hospitals
Please give an estimate of how many private rheumatology practices that include patients in your registry	50
Please give an estimate of how many departments of rheumatology at hospitals (not including university hospitals) that include patients in your registry	15
Please give an estimate of how many departments of rheumatology at university hospitals that include patients in your registry	5
When is the data registered? (Tick all that apply)	☒ at routine visits
Are all routine visits registered in your registry - or only some?	⊗ only some visits

Ethics

Is approval from a local ethics committee needed for a study on de-identified data (eg. a EuroSpA upload)? (Tick all that apply)	☒ yes<br>☒ no<br>☒ comment
Comment	For anonymized data, it should not be needed, but it depends a bit on the cantonal ethics committee, how they handle this.
Do patients need to sign informed consent to be included in your registry? (Tick all that apply)	☒ yes
Are any additional local approvals needed for a study on de-identified data (eg. a EuroSpA upload)? (Tick all that apply)	☒ yes
Please specify which additional approvals are needed	Approval from SCQM for research use of data

Funding

How is the registry funded? (Tick all that apply)	☒ industry<br>☒ other
Please estimate the percentage of funds coming from industry, eg. pharmaceutical company	90<br>((0-100%))
Please specify which other sources	Other foundations, Swiss Society of Rheumatology
Please estimate the percentage of funds coming from other sources	10<br>((0-100%))
The percentages add correctly up to a 100%	
What is the basis of participation by a clinic/department/office in the registry? (Tick all that apply)	☒ voluntary
Is the clinic/department/office financially compensated for registration?	⊗ no

Inclusion criteria

What event triggers the inclusion of a patient into the registry? (Tick all that apply)	☒ new diagnosis<br>☒ new treatment<br>☒ other
Please specify	A patient can be included at any time-point during the patient journey
Which criteria do you base the inclusion on? (Tick all that apply)	☒ diagnosis<br>☒ other
Is a minimum age required for inclusion?	☒ no

Which diagnoses are included in your registry?<br><br>(Tick all that apply)	☒ ankylosing spondylitis (AS)<br>☒ non-radiographic axial spondyloarthritis (nr-axSpA)<br>☒ psoriatic arthritis (PsA)<br>☒ rheumatoid arthritis
Which year did inclusion of AS patients begin?	2005
Which year did inclusion of nr-axSpA patients begin?	2005
Which year did inclusion of PsA patients begin?	2006
Please specify which additional inclusion criteria	Patient needs to speak one of the official country languages (DE, FR or IT)
Have the inclusion criteria changed over time?	⊗ no

Response was added on 2021-12-17 14:06:24.

Data management	
What are the options for data entry?<br>(Tick all that apply)	☒ paper based<br>☒ electronic
Are the data fields in your registry interactive, such that invalid or unprobable data is flagged when entered (edit checks)<br>(Tick all that apply)	☒ yes
Since when (year) has the data fields been interactive?	2009
Please describe any other data validation procedures that you may use	obligatory fields exist, such as start dates of drugs, with a minimal level of information of YYYY
How does your registry retain data management services?<br>(Tick all that apply)	☒ a researcher/administrative personal does data management beside other duties
Which is the background of your data manager(s)?<br>(Tick all that apply)	☒ data science/biostatistician/technical
Which are the most commonly used data analysis software/programming languages in your organization?<br>(Tick all that apply)	☒ R<br>☒ python
How is the data stored in the registry?<br>(Tick all that apply)	☒ a relational database framework (like SQL)
Which are the main data formats used for raw data extractions?<br>(Tick all that apply)	☒ simple text files, eg. csv, tsv
Where is your data collection platform hosted?<br>(Tick all that apply)	☒ external company
Who maintains your data collection platform?<br>(Tick all that apply)	☒ external company
How frequently is the database updated with the latest information?<br>(Tick all that apply)	☒ monthly<br>☒ other
Please specify	daily snapshot that are not saved for maintenance, monthly permanent snapshot
Is linkage to other databases or registries possible?	⊗ no

General comments	Linkage to other registries is, in principle possible, but it there is no unique identifier that is available across registries, so one has to do linkage e.g. with privacy preserving linkage or similarity linkage based on names or dob. Linkage has to be done for each research project separately and ethics approval is needed for this.

Response was added on 2021-12-17 14:06:29.

Demography

Please indicate which of the following variables are collected in your registry
(Tick all that apply)

☒ age (year of birth)
☒ sex
☒ weight
☒ height
☒ death

Time points for registration

	at inclusion in registry	at start/change of treatment	at follow-up visits	other
Weight	☒	☐	☒	☐
Height	☒	☐	☒	☐

How is vital status registered
(Tick all that apply)

☒ by healthstaff, manually
☒ other

Please specify

There is no fixed time interval for vital status registration. Therefore, death is an outcome that is very difficult to study with our data.

Diagnosis

How is a diagnosis registered?
(Tick all that apply)

☒ classification criteria
☒ other diagnostic categories

Specify which diagnostic categories

clinical diagnosis by the rheumatologist

Do you register
(Tick all that apply)

☒ date for diagnosis
☒ month of diagnosis
☒ year of diagnosis

Do you register
(Tick all that apply)

☒ date for symptom onset
☒ month of symptom onset
☒ year of symptom onset

Time points for registration

	at inclusion in registry	at start/change of treatment	at follow-up visits	other
Day/month/year of diagnosis	☒	☐	☒	☐

Day/month/year of symptom onset	☒	☐	☒	☐

Which classification criteria are registered?
(Tick all that apply)

☒ ASAS
☒ New York
☒ CASPAR

Time points for registration

	at inclusion in registry	at start/change of treatment	at follow-up visits	other
ASAS	☒	☐	☒	☐
New York	☒	☐	☒	☐
CASPAR	☒	☐	☒	☐

In which patients can you register ASAS? ⊗ AxSpA

Do you register individual ASAS classification items?
(Tick all that apply)

☒ yes

Do you register individual New York classification items?
(Tick all that apply)

☒ yes

Do you register individual CASPAR classification items?
(Tick all that apply)

☒ yes

Response was added on 2021-12-17 14:06:32.

Axial spondyloarthritis

Which disease status characteristics can be registered in axSpA patients?<br>(Tick all that apply)	☒ swollen joint count<br>☒ tender joint count<br>☒ enthesitis<br>☒ dactylitis<br>☒ physician global<br>☒ BASMI

Time points for registration

	at inclusion in registry	at start/change of treatment	at follow-up visits	other
Swollen joint count	☒	☒	☒	☐
Tender joint count	☒	☒	☒	☐
Enthesitis	☒	☒	☒	☐
Dactylitis	☒	☐	☒	☐
Physician global	☒	☒	☒	☐
BASMI	☒	☐	☒	☐

Swollen joints

How many swollen joint counts can be registered?<br>(Tick all that apply)	☒ 28<br>☒ 44<br>☒ 66
Do you register specific location of swollen joints?<br>(Tick all that apply)	☒ yes<br>☒ comments
Please specify	44 is recommended but doctor can switch to 28 or 66/68

Tender joints

How many tender joint counts can be registered?<br>(Tick all that apply)	☒ 28<br>☒ 44<br>☒ 68
Do you register specific location of tender joints?<br>(Tick all that apply)	☒ yes<br>☒ comments
Comments	tender joints were not collected in DB2 btw 2005 and 2020. From Jan 2021 onwards, it is part of the questionnaire

Enthesitis

Do you register specific location of enthesitis? (Tick all that apply)	☒ yes
Which locations are registered? (Tick all that apply)	☒ achilles tendon insertion<br>☒ plantar fascia insertion<br>☒ 1st costochondral joint<br>☒ 7th costochondral joint<br>☒ posterior superior iliac spine<br>☒ anterior superior iliac spine<br>☒ iliac crests<br>☒ 5th lumbar spinous process
Please indicate which specific indices that are used, if applicable	MASES +

Dactylitis

How is dactylitis assessed? (Tick all that apply)	☒ other
Other	dactylisit in the past 12 months? If so, was it detected clinically, in the MRI or in the Xray/CT or via sonography?

Physician Global

Please write the wording of the question regarding physician global	Global estimate of the disease activity by the physician (from context it is clear that it is about day of visit)<br>(translate into english if possible)

BASMI

Are the individidual BASMI components registered?	⊗ yes
Do you register the individual BASMI measurements (cm, degrees)?	⊗ yes
Which BASMI scale is used?	⊗ 3-point

Coxitis

Do you assess coxitis? If yes, please indicate how it is assessed	yes, coxitis in the past 12 months? If so, was it detected clinically, in the MRI or in the Xray/CT or via sonography?

General comment	About the visit structure:<br>We used the column: Follow-up visit to indicate what we collect at yearly controls and the column "at treatment change" to indicate what we collect in intermediate controls.<br><br>Yearly controls are very broad and cover many subjects. Intermediate controls focus on medication, disease activity, safety.<br><br>Visits at treatment changes are recommended by the swiss society of rheumatology but the system (database) does not trigger reminders for e.g. a 3-6 months follow-up visit after start of a b/tsDMARD.<br><br>Regarding BASMI: Our BASMI is three point. This means that we apply two thresholds per variable to split them into low, middle and hight and only calculate the score on the three.<br>We collect tragus to wall rather than occiput to wall and adjust for the tragus vs occiput by just using different thresholds. Ours are 3 and 18. The literature for the tragus shows stuff like 19 and 31 for that

Response was added on 2021-12-17 14:06:36.

Psoriatic arthritis

Which disease status characteristics can be registered in PsA patients? (Tick all that apply)	☒ swollen joint count<br>☒ tender joint count<br>☒ enthesitis<br>☒ dactylitis<br>☒ skin<br>☒ nails<br>☒ physician Global<br>☒ BASMI<br>☒ other
Please specify	HAQ

Time points for registration

	at inclusion in registry	at start/change of treatment	at follow-up visits	other
Swollen joint count	☒	☒	☒	☐
Tender joint count	☒	☒	☒	☐
Enthesitis	☒	☒	☒	☐
Dactylitis	☒	☒	☒	☐
Skin	☒	☒	☒	☐
Nails	☒	☒	☒	☐
Physician Global	☒	☒	☒	☐
BASMI	☒	☐	☒	☐

Swollen joints

How many swollen joint counts can be registered? (Tick all that apply)	☒ 28<br>☒ 44<br>☒ 66
Do you register specific location of swollen joints? (Tick all that apply)	☒ yes<br>☒ comments
Commont	66 is recommended

Tender joints

How many tender joint counts can be registered?<br>(Tick all that apply)	☒ 28<br>☒ 44<br>☒ 68
Do you register specific location of tender joints?<br>(Tick all that apply)	☒ yes<br>☒ comments
comments	68 is recommended

Entesitis

Do you register specific location of enthesitis?<br>(Tick all that apply)	☒ yes
Which locations are registered?<br>(Tick all that apply)	☒ achilles tendon insertion<br>☒ plantar fascia insertion<br>☒ 1st costochondral joint<br>☒ 7th costochondral joint<br>☒ posterior superior iliac spine<br>☒ anterior superior iliac spine<br>☒ iliac crests<br>☒ 5th lumbar spinous process
Please indicate which specific indices that are used, if applicable	MASES +

Dactylitis

How is dactylitis assessed?<br>(Tick all that apply)	☒ other
Other	dactylisit in the past 12 months? If so, was it detected clinically, in the MRI or in the Xray/CT or via sonography?

Skin

Which instruments are used for registering skin involvment in PsA?<br>(Tick all that apply)	☒ other
Other	7 scale question: none, almost none, light, light to moderate, moderate, moderate to severe, severe

Nails

Which instruments are used for registering nail involvment in PsA? (Tick all that apply)	☒ other
Other	3 types of nail problems: (none), oil points, pitted nails, onycholysis

Physician global

Please write the wording of the question regarding physician global	Global estimate of the disease activity by the physician (from context clear that it is at the visit)<br>(Translate into english if possible)

BASMI

Are the individidual BASMI components registered?	⊗ yes
Do you register the individual BASMI measurements (cm, degrees)?	⊗ yes
Which BASMI scale is used?	⊗ 3-point

Coxitis

Do you assess coxitis? If yes, please indicate how it is assessed	yes, coxitis in the past 12 months? If so, was it detected clinically, in the MRI or in the Xray/CT or via sonography?

Response was added on 2021-12-17 14:06:38.

Medication

Which therapies are registered in your registry? (Tick all that apply)	☒ biological dmards (bDMARDs), including targeted synthetic dmards (JAK)<br>☒ conventional synthetic dmards (csDMARDs)<br>☒ glucocorticoids<br>☒ NSAIDs

bDMARDs

Which year did bDMARD registration begin?	2005
Is it mandatory to register bDMARD therapy?	⊗ no

csDMARDs

Which year did csDMARD registration begin?	2005
Is it mandatory to register csDMARD therapy?	⊗ no

Glucocorticoids

Which year did glucocorticoid registration begin?	2005
Is it mandatory to register glucocorticoid therapy?	⊗ no
Is the mode of administration registered? (Tick all that apply)	☒ oral<br>☒ intraarticular
Are specific locations of injected joints registered?	⊗ no

NSAIDs

Which year did NSAID registration begin?	2005
Is it mandatory to register NSAID therapy?	⊗ no

What information regarding ONGOING medications is registered?

	start date	stop date	temporary start and stop dates	discontinuation reasons	dosage	frequency	administration mode
bDMARDs	☒	☒	☐	☒	☒	☒	☒
csDMARDs	☒	☒	☐	☒	☒	☒	☒
glucocorticoids	☒	☒	☐	☒	☒	☒	☒

Time points for registration (ONGOING medications)

	at inclusion in registry	at start/change of treatment	at follow-up visits	other
bDMARDs	☒	☒	☒	☐
csDMARDs	☒	☒	☒	☐
glucocorticoids	☒	☒	☒	☐
NSAIDs	☒	☒	☒	☐

What information regarding PAST medications is registered at inclusion in registry?

	start date	stop date	temporary start and stop dates	discontinuation reasons	dosage	name of drug
bDMARDs	☒	☒	☐	☒	☒	☒
csDMARDs	☒	☒	☐	☒	☒	☒

Discontinuation reasons

What are the possible reasons for discontinuation of a bDMARD?<br>(Tick all that apply)	☒ lack of efficacy<br>☒ adverse events<br>☒ remission<br>☒ other
Please specify	from 2021 on, there is more detailed reasons for discontinuation
Is it possible to register multiple reasons for discontinuation?	⊗ yes
How is it decided which is the primary reason for discontinuation?	up to the investigator
Does your registry link to prescription database or other external data sources on prescriptions on a regular basis as input to registry?<br>(Tick all that apply)	☒ no

Response was added on 2021-12-17 14:06:41.

Patient reported outcomes

Which patient reported outcomes (PROs) are registered in axSpA and/or PsA patients? (Tick all that apply)	☒ BASDAI<br>☒ BASFI<br>☒ pain<br>☒ nightly pain<br>☒ fatigue<br>☒ global disease<br>☒ HAQ<br>☒ EQ-5D<br>☒ other
Please specify	SF12, DLQI skin quality of life, HAQ, ASAS-HI since Nov 2018, GPAQ exercise score since Oct 2021
Are any of the PROs registered in either axSpA or PsA only?	⊗ yes
Which PROs are registered uniquely in either axSpA or PsA?	BASDAI and BASFI in axSpA only, DLQI and HAQ in PsA only

Mode of registration

What are the options for registration of PROs? (Tick all that apply)	☒ paper forms<br>☒ on screen in waiting room<br>☒ through website/app

Time points for registration

	at inclusion in registry	at start/change of treatment	at follow-up visits	other
BASDAI	☒	☒	☒	☐
BASFI	☒	☒	☒	☐
Pain	☒	☒	☒	☐
Nightly pain	☒	☒	☒	☐
Fatigue	☒	☒	☒	☐
Global disease	☒	☒	☒	☐
HAQ	☒	☒	☒	☐
EQ-5D	☒	☒	☒	☐

BASDAI

Are the individidual BASDAI components registered?	⊗ yes

BASFI

Are the individidual BASFI components registered?	⊗ yes

Pain, fatigue and global assessments

Please write the wording of the question relating to pain	Wie würden Sie die Stärke Ihrer Schmerzen insgesamt in den letzten 7 Tagen einschätzen? How would you rate your overall pain in the past 7 days?<br>(please translate into english if possible)
Please write the wording of the question relating to nightly pain	Wie würden Sie die Stärke Ihrer Schmerzen in der Nacht insgesamt in den letzten 7 Tagen einschätzen? How would you rate your overall pain in the night in the past 7 days?<br>(please translate into english if possible)
Please write the wording of the question relating to fatigue	BASDAI-1 Wie würden Sie Ihre allgemeine Müdigkeit und Erschöpfung durchschnittlich in den letzten 7 Tagen beschreiben? How would you rate your overall tiredness and fatigue on average in the past 7 days?<br>(please translate into english if possible)
Please write the wording of the question relating to global assessment of disease	Wie aktiv ist Ihre Krankheit heute? How active is your disease today?<br>(please translate into english if possible)

HAQ

Are the individual HAQ items registered?	⊗ yes
Which HAQ versions may be used in your registry? (Tick all that apply)	☒ stanford HAQ DI with adjustment for use of aids and devices

EQ-5D

Are the individual EQ-5D items registered?	⊗ yes
Which EQ-5D version is used?	3L
Which algorithm is used?	'European index', see DOI 10.1007/s10198-003-0182-5

Response was added on 2021-12-17 14:06:43.

Laboratory

Which laboratory test can be registered? (Tick all that apply)

- ☒ ESR
- ☒ CRP
- ☒ hemoglobin
- ☒ ALAT
- ☒ creatinine
- ☒ HLA-B27
- ☒ IgM-RF
- ☒ stored samples available for later analyses (biobank)

Time points for registration

	at inclusion in registry	at start/change of treatment	at follow-up visits	other
ESR	☒	☒	☒	☐
CRP	☒	☒	☒	☐
Hemoglobin	☒	☒	☒	☐
ALAT	☒	☒	☒	☐
Creatinine	☒	☒	☒	☐
Stored samples available for later analyses (biobank)	☒	☒	☒	☐

Biobank

Which sample types are collected? (Tick all that apply)

- ☒ serum
- ☒ other

Please specify: Blood for DNA

How are laboratory test results registered? (Tick all that apply)

- ☒ entered by health care staff

Response was added on 2021-12-17 14:06:45.

Imaging

Which imaging modalities can be registered? (Tick all that apply)	☒ X-ray

	date of examination	full image report	image file	scoring system	+/- progression	other
What information on each x-ray exam is registered?	☒	☐	☒	☒	☐	☐

X-ray

Which anatomical regions can be registered? (Tick all that apply)	☒ spine radiographs<br>☒ sacroiliac joint radiographs<br>☒ hands and feet
Which scoring system?	We perform mNY ISG scoring and (in context of research project) MSASSS spine score

Frequency of image registration

Please comment on the frequency of registration of the various image modalities, eg. at specified time points, as needed, in connection with research projects or other.	Images are recommended in time intervals of two years

Response was added on 2021-12-17 14:06:48.

Comorbidities

Which extraarticular manifestations and comorbid conditions are registered?
(Tick all that apply)

☒ uveitis
☒ psoriasis
☒ inflammatory bowel disease (IBD)
☒ ischemic heart disease (IHD)
☒ cerebrovascular disease (CVD)
☒ hypertension
☒ diabetes (DM)
☒ dyslipidemia
☒ osteoporosis
☒ chronic kidney insufficiency (CKI)
☒ chronic liver disease, eg. hepatitis, cirrhosis
☒ solid cancer
☒ hematological cancer
☒ depression
☒ tuberculosis (TB)
☒ fibromyalgia
☒ other

Time points for registration

	at inclusion in registry	at start/change of treatment	at follow-up visits	other
uveitis	☒	☒	☒	☐
psoriasis	☒	☒	☒	☐
IBD	☒	☒	☒	☐
IHD	☒	☒	☒	☐
CVD	☒	☒	☒	☐
hypertension	☒	☒	☒	☐
DM	☒	☒	☒	☐
dyslipidemia	☒	☒	☒	☐
osteoporosis	☒	☒	☒	☐
CKI	☒	☒	☒	☐
chronic liver disease	☒	☒	☒	☐
solid cancer	☒	☒	☒	☐
hematological cancer	☒	☒	☒	☐
depression	☒	☒	☒	☐
TB	☒	☒	☒	☐
fibromyalgia	☒	☒	☒	☐
other	☒	☒	☒	☒

Please specify (other)	Up to 2016, we collected a list of comorbidities, that was shown and adapted at each visit. Since 2016, we have a health issue system with categories and sub-categories and within these, a text field for diagnosis. Events can be entered with full or partial date. All already known comorbs and events are shown at each visit with the question to confirm or adapt (add events).

Mode of registration - how are comorbid conditions registered?

	patient-reported	by health-staff	linkage from other registry
uveitis	☐	☒	☐
psoriasis	☐	☒	☐
IBD	☐	☒	☐
IHD	☐	☒	☐
CVD	☐	☒	☐
hypertension	☐	☒	☐
DM	☐	☒	☐
dyslipidemia	☐	☒	☐
osteoporosis	☐	☒	☐
CKI	☐	☒	☐
chronic liver disease	☐	☒	☐
solid cancer	☐	☒	☐
hematological cancer	☐	☒	☐
depression	☐	☒	☐
TB	☐	☒	☐
fibromyalgia	☐	☒	☐
other	☐	☒	☐

Do you use ICD-10 codes for registration?	⊗ no

Response was added on 2021-12-17 14:06:50.

Lifestyle

Which lifestyle parameters are registered? (Tick all that apply)	☒ smoking<br>☒ alcohol consumption<br>☒ physical activity<br>☒ other
Please specify	work status etc

Time points for registration

	at inclusion in registry	at start/change of treatment	at follow-up visits	other
Smoking	☒	☐	☒	☐
Alcohol	☒	☐	☒	☐
Physical activity	☒	☐	☒	☐

Smoking

How is smoking status characterised? (Tick all that apply)	☒ current<br>☒ former<br>☒ never
Do you register a start date? (Tick all that apply)	☒ no date is registered
Do you register a stop date for former smokers?	⊗ No
How is average number of smoked cigarettes registered? (Tick all that apply)	☒ other
Please specify	Psa has questions for the two questions to calculate pack-years. axSpa has no further details

Alcohol

How is alcohol consumption quantified? (Tick all that apply)	☒ other
Please specify	none, rarely, daily, more than daily

Physical activity

How is physical activity defined?<br>(Tick all that apply)	⊗ other definition
Please specify	Now full GPAQ. Old PSA version tracked none vs less than 1h/w vs 1 to 2 h/w vs more for activities with sweating or elevated heart rate, Old axSpa just ask for exercises per week
How is physical activity quantified?<br>(Tick all that apply)	☒ other
Please specify	See 'Please specify' above

Response was added on 2021-12-17 14:08:02.

Safety

Can you register adverse events in your registry? (Tick all that apply)	☒ yes, directly into registry
Is it mandatory to register adverse events through your registry?	⊗ no
Which adverse events are registered in your registry? (Tick all that apply)	☒ non-serious adverse events<br>☒ serious adverse events?

Information on adverse events

	date of event	MeddRA	ICD10-code	outcome	other
Non serious AE	☒	☐	☐	☒	☒
Serious AE	☒	☐	☐	☒	☒

Please specify (non serious AE)	diagnosis of AE, suspected drug, life threatening, hospitalization, and some other questions from the swissmedic adverse event reporting form
Please specify (serious AE)	as with non-serious.
General comments	Information on date of event can be in the format yyyy, mm/yyyy or full date OR is in some cases unspecified. Therefore, we often are dealing with an interval information on the occurence / start of the event.

Participant information

Record ID	5
Name	Ana Rodrigues
Registry	Reuma.pt
E-mail	anamfrodrigues@gmail.com
Deltager	⊗ ja

Response was added on 2022-01-19 18:14:16.

General registry information

What is the status of your registry?	⊗ running and including patients

Coverage

Please estimate how many (percentage) of the eligible spondyloarthritis patients in your country, that are registered	10
Please estimate how many (percentage) of the eligible psoriatic arthritis patients in your country, that are registered	15
How did you obtain the coverage estimate above? (Tick all that apply)	☒ other
Please specify	comparison of the estemated prevalence of these disease in Portugal with the patient registered
Which institutions/organisations can include patients in your registry? (Tick all that apply)	☒ private rheumatology practices<br>☒ departments of rheumatology at hospitals<br>☒ departments of rheumatology at university hospitals
Please give an estimate of how many private rheumatology practices that include patients in your registry	75%
Please give an estimate of how many departments of rheumatology at hospitals (not including university hospitals) that include patients in your registry	98%
Please give an estimate of how many departments of rheumatology at university hospitals that include patients in your registry	100%
When is the data registered? (Tick all that apply)	☒ at routine visits
Are all routine visits registered in your registry - or only some?	⊗ all visits

Ethics

Is approval from a local ethics committee needed for a study on de-identified data (eg. a EuroSpA upload)? (Tick all that apply)	☒ no
Do patients need to sign informed consent to be included in your registry? (Tick all that apply)	☒ yes
Are any additional local approvals needed for a study on de-identified data (eg. a EuroSpA upload)? (Tick all that apply)	☒ no

Funding

How is the registry funded? (Tick all that apply)	☒ from research grants<br>☒ industry
Please estimate the percentage of funds coming from research grants	15<br>((0-100%))
Please estimate the percentage of funds coming from industry, eg. pharmaceutical company	85<br>((0-100%))
The percentages add correctly up to a 100%	
What is the basis of participation by a clinic/department/office in the registry? (Tick all that apply)	☒ voluntary
Is the clinic/department/office financially compensated for registration?	⊗ no

Inclusion criteria

What event triggers the inclusion of a patient into the registry? (Tick all that apply)	☒ new diagnosis<br>☒ new treatment
Which criteria do you base the inclusion on? (Tick all that apply)	☒ diagnosis
Is a minimum age required for inclusion?	☒ no
Which diagnoses are included in your registry?<br><br>(Tick all that apply)	☒ ankylosing spondylitis (AS)<br>☒ non-radiographic axial spondyloarthritis (nr-axSpA)<br>☒ psoriatic arthritis (PsA)<br>☒ rheumatoid arthritis<br>☒ other
Which year did inclusion of AS patients begin?	2009
Which year did inclusion of nr-axSpA patients begin?	NA

Which year did inclusion of PsA patients begin?	2009
Which other diagnoses are included?	systemic vasculitis; Idiopatic juvenil arthritis; myositis; systemic Lupus erythematosus;s sjogren
Have the inclusion criteria changed over time?	⊗ no

Response was added on 2022-01-19 18:40:55.

**Data management**	
What are the options for data entry?<br>(Tick all that apply)	☒ electronic
Are the data fields in your registry interactive, such that invalid or unprobable data is flagged when entered (edit checks)<br>(Tick all that apply)	☒ yes
Since when (year) has the data fields been interactive?	2012
Please describe any other data validation procedures that you may use	manually
How does your registry retain data management services?<br>(Tick all that apply)	☒ person employed as data manager
Which is the background of your data manager(s)?<br>(Tick all that apply)	☒ data science/biostatistician/technical
How is the data stored in the registry?<br>(Tick all that apply)	☒ a relational database framework (like SQL)
Which are the main data formats used for raw data extractions?<br>(Tick all that apply)	☒ excel
Where is your data collection platform hosted?<br>(Tick all that apply)	☒ external company
Who maintains your data collection platform?<br>(Tick all that apply)	☒ data manager
How frequently is the database updated with the latest information?<br>(Tick all that apply)	☒ real-time
Is linkage to other databases or registries possible?	⊗ no

Response was added on 2022-01-19 18:55:36.

Demography

Please indicate which of the following variables are collected in your registry (Tick all that apply)	☒ age (year of birth)<br>☒ sex<br>☒ ethnicity<br>☒ weight<br>☒ height<br>☒ death

Time points for registration

	at inclusion in registry	at start/change of treatment	at follow-up visits	other
Weight	☒	☐	☒	☐
Height	☒	☐	☒	☐

How is vital status registered (Tick all that apply)	☒ by healthstaff, manually

Diagnosis

How is a diagnosis registered? (Tick all that apply)	☒ classification criteria<br>☒ other diagnostic categories
Specify which diagnostic categories	expert opinion
Do you register (Tick all that apply)	☒ date for diagnosis<br>☒ month of diagnosis<br>☒ year of diagnosis
Do you register (Tick all that apply)	☒ date for symptom onset<br>☒ month of symptom onset<br>☒ year of symptom onset

Time points for registration

	at inclusion in registry	at start/change of treatment	at follow-up visits	other
Day/month/year of diagnosis	☒	☐	☐	☒
Day/month/year of symptom onset	☒	☐	☐	☒

Please specify (diagnosis)	we can always registry this information at any time of the follow-up
Please specify (symptom onset)	we can always registry this information at any time of the follow-up

Which classification criteria are registered?<br>(Tick all that apply)	☒ ASAS<br>☒ New York<br>☒ CASPAR

Time points for registration

	at inclusion in registry	at start/change of treatment	at follow-up visits	other
ASAS	☒	☐	☐	☒
New York	☒	☐	☐	☒
CASPAR	☒	☐	☐	☒

Please specify (ASAS)	we can always registry this information at any time of the follow-up
Please specify (New York)	we can always registry this information at any time of the follow-up
Please specify (CASPAR)	we can always registry this information at any time of the follow-up
In which patients can you register ASAS?	⊗ AxSpA
Do you register individual ASAS classification items?<br>(Tick all that apply)	☒ yes
Do you register individual New York classification items?<br>(Tick all that apply)	☒ yes
Do you register individual CASPAR classification items?<br>(Tick all that apply)	☒ yes

Response was added on 2022-01-19 19:17:46.

Axial spondyloarthritis

Which disease status characteristics can be registered in axSpA patients?
(Tick all that apply)

☒ swollen joint count
☒ tender joint count
☒ enthesitis
☒ physician global
☒ BASMI
☒ other

Please specify: ASDAS

Time points for registration

	at inclusion in registry	at start/change of treatment	at follow-up visits	other
Swollen joint count	☒	☒	☒	☐
Tender joint count	☒	☒	☒	☐
Enthesitis	☒	☒	☒	☐
Physician global	☒	☒	☒	☐
BASMI	☒	☒	☒	☐

Swollen joints

How many swollen joint counts can be registered?
(Tick all that apply)

☒ 66

Do you register specific location of swollen joints?
(Tick all that apply)

☒ yes

Tender joints

How many tender joint counts can be registered?
(Tick all that apply)

☒ 68

Do you register specific location of tender joints?
(Tick all that apply)

☒ yes

Enthesitis

Do you register specific location of enthesitis? (Tick all that apply)	☒ yes
Which locations are registered? (Tick all that apply)	☒ all of the below
Please indicate which specific indices that are used, if applicable	SPARCC and MACES

Physician Global

Please write the wording of the question regarding physician global	Disease activity according to physician opinion (0-100 mm)<br>(translate into english if possible)

BASMI

Are the individidual BASMI components registered?	⊗ yes
Do you register the individual BASMI measurements (cm, degrees)?	⊗ yes
Which BASMI scale is used?	⊗ 11-point

Coxitis

Do you assess coxitis? If yes, please indicate how it is assessed	no

Response was added on 2022-01-19 19:23:00.

Psoriatic arthritis

Which disease status characteristics can be registered in PsA patients? (Tick all that apply)	☒ swollen joint count<br>☒ tender joint count<br>☒ enthesitis<br>☒ dactylitis<br>☒ skin

Time points for registration

	at inclusion in registry	at start/change of treatment	at follow-up visits	other
Swollen joint count	☒	☒	☒	☐
Tender joint count	☒	☒	☒	☐
Enthesitis	☒	☒	☒	☐
Dactylitis	☒	☒	☒	☐
Skin	☒	☒	☒	☐

Swollen joints

How many swollen joint counts can be registered? (Tick all that apply)	☒ 66
Do you register specific location of swollen joints? (Tick all that apply)	☒ yes

Tender joints

How many tender joint counts can be registered? (Tick all that apply)	☒ 68
Do you register specific location of tender joints? (Tick all that apply)	☒ yes

Entesitis

Do you register specific location of enthesitis? (Tick all that apply)	☒ yes
Which locations are registered? (Tick all that apply)	☒ all of the below
Please indicate which specific indices that are used, if applicable	SPARCC ; MASES

Dactylitis

How is dactylitis assessed?<br>(Tick all that apply)	☒ as part of classification criteria<br>☒ as a count

Skin

Which instruments are used for registering skin involvment in PsA?<br>(Tick all that apply)	☒ PASI

Coxitis

Do you assess coxitis? If yes, please indicate how it is assessed	no

Response was added on 2022-01-19 20:04:03.

Medication

Which therapies are registered in your registry? (Tick all that apply)	☒ biological dmards (bDMARDs), including targeted synthetic dmards (JAK)<br>☒ conventional synthetic dmards (csDMARDs)<br>☒ glucocorticoids<br>☒ NSAIDs<br>☒ medication for comorbidity

bDMARDs

Which year did bDMARD registration begin?	2009
Is it mandatory to register bDMARD therapy?	⊗ no

csDMARDs

Which year did csDMARD registration begin?	2009
Is it mandatory to register csDMARD therapy?	⊗ no

Glucocorticoids

Which year did glucocorticoid registration begin?	2009
Is it mandatory to register glucocorticoid therapy?	⊗ no
Is the mode of administration registered? (Tick all that apply)	☒ oral<br>☒ intramuscular<br>☒ intraarticular
Are specific locations of injected joints registered?	⊗ no

NSAIDs

Which year did NSAID registration begin?	2009
Is it mandatory to register NSAID therapy?	⊗ no

Medicaton for comorbidities

What types of medical therapy for comorbidities are registered?

it is possible to register any treatment for chronic non communicable disease but is underreported

What information regarding ONGOING medications is registered?

	start date	stop date	temporary start and stop dates	discontinuation reasons	dosage	frequency	administration mode
bDMARDs	☒	☒	☒	☒	☒	☒	☒
csDMARDs	☒	☒	☒	☒	☒	☒	☒
glucocorticoids	☒	☒	☒	☐	☒	☒	☒
NSAIDs	☒	☐	☐	☐	☒	☒	☒
medication for comorbidity	☒	☐	☐	☐	☒	☒	☒

Time points for registration (ONGOING medications)

	at inclusion in registry	at start/change of treatment	at follow-up visits	other
bDMARDs	☒	☒	☒	☐
csDMARDs	☒	☒	☒	☐
glucocorticoids	☒	☒	☒	☐
NSAIDs	☒	☒	☒	☐
medication for comorbidity	☒	☐	☐	☐

What information regarding PAST medications is registered at inclusion in registry?

	start date	stop date	temporary start and stop dates	discontinuation reasons	dosage	name of drug
bDMARDs	☒	☒	☐	☒	☒	☒
csDMARDs	☒	☒	☐	☒	☒	☒
glucocorticoids	☒	☒	☐	☒	☒	☒
NSAIDs	☒	☒	☐	☐	☐	☐

Discontinuation reasons

What are the possible reasons for discontinuation of a bDMARD?
(Tick all that apply)

☒ lack of efficacy
☒ adverse events
☒ remission
☒ pregnancy wish
☒ infection
☒ surgery
☒ death

Is it possible to register multiple reasons for discontinuation?

⊗ no

Does your registry link to prescription database or other external data sources on prescriptions on a regular basis as input to registry?<br>(Tick all that apply)	☒ no

Response was added on 2022-01-20 12:45:29.

Patient reported outcomes

Which patient reported outcomes (PROs) are registered in axSpA and/or PsA patients?
(Tick all that apply)

☒ BASDAI
☒ BASFI
☒ pain
☒ nightly pain
☒ fatigue
☒ global disease
☒ HAQ
☒ EQ-5D

Are any of the PROs registered in either axSpA or PsA only?

⊗ no, all PROs are registered in both diagnoses

Mode of registration

What are the options for registration of PROs?
(Tick all that apply)

☒ through interview with health-staff
☒ on screen in waiting room
☒ through website/app

Time points for registration

	at inclusion in registry	at start/change of treatment	at follow-up visits	other
BASDAI	☒	☒	☒	☐
BASFI	☒	☒	☒	☐
Pain	☒	☒	☒	☐
Nightly pain	☒	☒	☒	☐
Fatigue	☒	☒	☒	☐
Global disease	☒	☒	☒	☐
HAQ	☒	☒	☒	☐
EQ-5D	☒	☒	☒	☐

BASDAI

Are the individidual BASDAI components registered?

⊗ yes

BASFI

Are the individidual BASFI components registered?	⊗ yes

Pain, fatigue and global assessments

Please write the wording of the question relating to pain	Please indicate the level of pain that you felt in your spine at any moment (day or nigth) during last week<br>(please translate into english if possible)
Please write the wording of the question relating to nightly pain	Please indicate the level of pain that you felt in your spine during the night during last week<br>(please translate into english if possible)
Please write the wording of the question relating to fatigue	FACIT questionnaire<br>(please translate into english if possible)
Please write the wording of the question relating to global assessment of disease	considering the way the disease disturbs you, how do you feel during the last week?<br>(please translate into english if possible)

HAQ

Are the individual HAQ items registered?	⊗ yes
Which HAQ versions may be used in your registry?<br>(Tick all that apply)	☒ stanford HAQ DI with adjustment for use of aids and devices

EQ-5D

Are the individual EQ-5D items registered?	⊗ yes
Which EQ-5D version is used?	eq-5d-3l
Which algorithm is used?	the portuguese validated algorithm
General comments	EQ-5D Portuguese population norms.<br>Ferreira LN, Ferreira PL, Pereira LN, Oppe M.<br>Qual Life Res. 2014 Mar;23(2):425-30. doi:<br>10.1007/s11136-013-0488-4. Epub 2013 Aug 3.<br>PMID: 23912856

Response was added on 2022-01-20 12:46:39.

Laboratory

Which laboratory test can be registered?
(Tick all that apply)

- ☒ ESR
- ☒ CRP
- ☒ hemoglobin
- ☒ ALAT
- ☒ creatinine
- ☒ Hba1c
- ☒ cholesterol
- ☒ HLA-B27
- ☒ IgM-RF
- ☒ stored samples available for later analyses (biobank)

Time points for registration

	at inclusion in registry	at start/change of treatment	at follow-up visits	other
ESR	☒	☒	☒	☐
CRP	☒	☒	☒	☐
Hemoglobin	☒	☒	☒	☐
ALAT	☒	☒	☒	☐
Creatinine	☒	☒	☒	☐
Hba1c	☒	☒	☒	☐
Cholesterol	☒	☒	☒	☐
Stored samples available for later analyses (biobank)	☒	☒	☒	☐

Cholesterol

Which specific cholesterol types are registered?
(Tick all that apply)

- ☒ total cholesterol
- ☒ LDL
- ☒ HDL
- ☒ triglycerid

Biobank

Which sample types are collected?
(Tick all that apply)

- ☒ whole-blood
- ☒ serum
- ☒ plasma
- ☒ joint fluid

How are laboratory test results registered?
(Tick all that apply)

- ☒ electronically tranfered from lab system
- ☒ entered by health care staff

Response was added on 2022-01-20 12:55:55.

Imaging

Which imaging modalities can be registered? (Tick all that apply)	☒ Magnetic Resonance Imaging (MRI)<br>☒ X-ray<br>☒ DXA<br>☒ Ultrasound (US)<br>☒ Computer Tomography (CT)

Information on each image

	date of examination	full image report	image file	scoring system	+/- progression	other
What information on each MRI exam is registered?	☒	☐	☒	☐	☐	☐
What information on each x-ray exam is registered?	☒	☐	☒	☐	☐	☐
What information on each DXA exam is registered?	☒	☐	☒	☐	☐	☐
What information on each US exam is registered?	☒	☐	☒	☐	☐	☐
What information on each CT exam is registered?	☒	☐	☒	☐	☐	☐

MRI

Which anatomical regions can be registered? (Tick all that apply)	☒ spine MRI<br>☒ other
Please specify	peripheral joints

X-ray

Which anatomical regions can be registered? (Tick all that apply)	☒ spine radiographs<br>☒ hands and feet

DEXA

Which anatomical regions can be registered? (Tick all that apply)	☒ lumbar spine<br>☒ femoral neck<br>☒ wrist

Ultrasound

Please indicate which anatomical regions that can be registered	all

CT

Please indicate which anatomical regions that can be registered?	joints, spine, pelvic

Frequency of image registration

Please comment on the frequency of registration of the various image modalities, eg. at specified time points, as needed, in connection with research projects or other.	Although Reuma.pt has the ability to register all imaging, the researchers do not register this information on reuma.pt
General comments	Imaging is not regularly register in Reuma.pt

Response was added on 2022-01-20 13:26:17.

Comorbidities

Which extraarticular manifestations and comorbid conditions are registered?
(Tick all that apply)

☒ uveitis
☒ psoriasis
☒ inflammatory bowel disease (IBD)
☒ ischemic heart disease (IHD)
☒ cerebrovascular disease (CVD)
☒ hypertension
☒ diabetes (DM)
☒ dyslipidemia
☒ osteoporosis
☒ chronic kidney insufficiency (CKI)
☒ chronic liver disease, eg. hepatitis, cirrhosis
☒ solid cancer
☒ hematological cancer
☒ depression
☒ tuberculosis (TB)
☒ fibromyalgia

Time points for registration

	at inclusion in registry	at start/change of treatment	at follow-up visits	other
uveitis	☒	☐	☒	☐
psoriasis	☒	☐	☒	☐
IBD	☒	☐	☒	☐
IHD	☒	☐	☒	☐
CVD	☒	☐	☒	☐
hypertension	☒	☐	☒	☐
DM	☒	☐	☒	☐
dyslipidemia	☒	☐	☒	☐
osteoporosis	☒	☐	☒	☐
CKI	☒	☐	☒	☐
chronic liver disease	☒	☐	☒	☐
solid cancer	☒	☐	☒	☐
hematological cancer	☒	☐	☒	☐
depression	☒	☐	☒	☐
TB	☒	☐	☒	☐
fibromyalgia	☒	☐	☒	☐

Mode of registration - how are comorbid conditions registered?

	patient-reported	by health-staff	linkage from other registry
uveitis	☐	☒	☐
psoriasis	☐	☒	☐
IBD	☐	☒	☐
IHD	☐	☒	☐
CVD	☐	☒	☐
hypertension	☐	☒	☐
DM	☐	☒	☐
dyslipidemia	☐	☒	☐
osteoporosis	☐	☒	☐
CKI	☐	☒	☐
chronic liver disease	☐	☒	☐
solid cancer	☐	☒	☐
hematological cancer	☐	☒	☐
depression	☐	☒	☐
TB	☐	☒	☐
fibromyalgia	☐	☒	☐

Do you use ICD-10 codes for registration? ⊗ no

Response was added on 2022-01-20 13:29:51.

Lifestyle

Which lifestyle parameters are registered? (Tick all that apply)	☒ smoking<br>☒ alcohol consumption

Time points for registration

	at inclusion in registry	at start/change of treatment	at follow-up visits	other
Smoking	☒	☐	☐	☐
Alcohol	☒	☐	☐	☐

Smoking

How is smoking status characterised? (Tick all that apply)	☒ current<br>☒ former<br>☒ never
Do you register a start date? (Tick all that apply)	☒ no date is registered
Do you register a stop date for former smokers?	⊗ yes
How is average number of smoked cigarettes registered? (Tick all that apply)	☒ other
Please specify	pack-day and number of years

Alcohol

How is alcohol consumption quantified? (Tick all that apply)	☒ other
Please specify	number of units/day

Response was added on 2022-01-20 13:30:12.

Safety

Can you register adverse events in your registry? (Tick all that apply)	☒ yes, directly into registry
Is it mandatory to register adverse events through your registry?	⊗ no
Which adverse events are registered in your registry? (Tick all that apply)	☒ non-serious adverse events<br>☒ serious adverse events?

Information on adverse events

	date of event	MeddRA	ICD10-code	outcome	other
Non serious AE	☒	☒	☐	☒	☐
Serious AE	☒	☒	☐	☒	☐

Participant information

Record ID	6
Name	Dan Nordstrøm
Registry	ROB-FIN
E-mail	Dan.Nordstrom@hus.fi
Deltager	⊗ ja

Response was added on 2021-11-24 13:09:13.

General registry information

What is the status of your registry?	⊗ running and including patients

Coverage

Please estimate how many (percentage) of the eligible spondyloarthritis patients in your country, that are registered	60
Please estimate how many (percentage) of the eligible psoriatic arthritis patients in your country, that are registered	60
How did you obtain the coverage estimate above? (Tick all that apply)	☒ by prior study of coverage
Which institutions/organisations can include patients in your registry? (Tick all that apply)	☒ departments of rheumatology at hospitals<br>☒ departments of rheumatology at university hospitals
Please give an estimate of how many departments of rheumatology at hospitals (not including university hospitals) that include patients in your registry	10
Please give an estimate of how many departments of rheumatology at university hospitals that include patients in your registry	5
When is the data registered? (Tick all that apply)	☒ at routine visits
Are all routine visits registered in your registry - or only some?	⊗ all visits

Ethics

Is approval from a local ethics committee needed for a study on de-identified data (eg. a EuroSpA upload)? (Tick all that apply)	☒ no<br>☒ comment
Comment	Based on permit from Finnish Institute for Health and Welfare
Do patients need to sign informed consent to be included in your registry? (Tick all that apply)	☒ no

Are any additional local approvals needed for a study on de-identified data (eg. a EuroSpA upload)?<br>(Tick all that apply)	☒ yes
Please specify which additional approvals are needed	Permit from Social and Health Data permit Authority FINDATA

Funding

How is the registry funded?<br>(Tick all that apply)	☒ from research grants<br>☒ industry
Please estimate the percentage of funds coming from research grants	80<br>((0-100%))
Please estimate the percentage of funds coming from industry, eg. pharmaceutical company	20<br>((0-100%))
The percentages add correctly up to a 100%	
What is the basis of participation by a clinic/department/office in the registry?<br>(Tick all that apply)	☒ voluntary
Is the clinic/department/office financially compensated for registration?	⊗ no

Inclusion criteria

What event triggers the inclusion of a patient into the registry?<br>(Tick all that apply)	☒ new diagnosis<br>☒ new treatment
Which criteria do you base the inclusion on?<br>(Tick all that apply)	☒ diagnosis
Is a minimum age required for inclusion?	☒ yes, 18 years or above
Which diagnoses are included in your registry?<br><br>(Tick all that apply)	☒ ankylosing spondylitis (AS)<br>☒ non-radiographic axial spondyloarthritis (nr-axSpA)<br>☒ psoriatic arthritis (PsA)<br>☒ rheumatoid arthritis
Which year did inclusion of AS patients begin?	2000
Which year did inclusion of nr-axSpA patients begin?	2013
Which year did inclusion of PsA patients begin?	2000
Have the inclusion criteria changed over time?	⊗ yes
How and when did inclusion criteria change?	ASAS and CAspar criteria entered in 2020

Response was added on 2021-11-24 13:09:21.

**Data management**	
What are the options for data entry?<br>(Tick all that apply)	☒ electronic
Are the data fields in your registry interactive, such that invalid or unprobable data is flagged when entered (edit checks)<br>(Tick all that apply)	☒ no
How does your registry retain data management services?<br>(Tick all that apply)	☒ person employed as data manager<br>☒ a researcher/administrative personal does data management beside other duties
Which is the background of your data manager(s)?<br>(Tick all that apply)	☒ data science/biostatistician/technical
Which are the most commonly used data analysis software/programming languages in your organization?<br>(Tick all that apply)	☒ R
How is the data stored in the registry?<br>(Tick all that apply)	☒ other
Please specify	R-derived format
Which are the main data formats used for raw data extractions?<br>(Tick all that apply)	☒ r-derived format
Where is your data collection platform hosted?<br>(Tick all that apply)	☒ at a hopital in a rheumatology department
Who maintains your data collection platform?<br>(Tick all that apply)	☒ data manager
How frequently is the database updated with the latest information?<br>(Tick all that apply)	☒ other
Please specify	On demand from each center.
Is linkage to other databases or registries possible?	⊗ yes
Which registries can be linked to?<br>(Tick all that apply)	☒ mortality registry<br>☒ prescription registry<br>☒ comorbidity<br>☒ electronic medical records

Response was added on 2021-11-24 13:10:32.

Demography

Please indicate which of the following variables are collected in your registry
(Tick all that apply)

☒ age (year of birth)
☒ sex
☒ weight
☒ height

Time points for registration

	at inclusion in registry	at start/change of treatment	at follow-up visits	other
Weight	☒	☐	☐	☐
Height	☒	☐	☐	☐

Diagnosis

How is a diagnosis registered?
(Tick all that apply)

☒ through ICD-10 codes
☒ classification criteria

Do you register
(Tick all that apply)

☒ month of diagnosis

Do you register
(Tick all that apply)

☒ month of symptom onset

Time points for registration

	at inclusion in registry	at start/change of treatment	at follow-up visits	other
Day/month/year of diagnosis	☒	☐	☐	☐
Day/month/year of symptom onset	☒	☐	☐	☐

Which classification criteria are registered?
(Tick all that apply)

☒ ASAS
☒ New York
☒ CASPAR

Time points for registration

	at inclusion in registry	at start/change of treatment	at follow-up visits	other
ASAS	☒	☐	☐	☐
New York	☒	☐	☐	☐
CASPAR	☒	☐	☐	☐

In which patients can you register ASAS?	⊗ AxSpA
Do you register individual ASAS classification items? (Tick all that apply)	☒ yes
Do you register individual New York classification items? (Tick all that apply)	☒ yes
Do you register individual CASPAR classification items? (Tick all that apply)	☒ yes

Response was added on 2021-12-14 17:04:39.

Axial spondyloarthritis

Which disease status characteristics can be registered in axSpA patients?
(Tick all that apply)

☒ swollen joint count
☒ tender joint count
☒ enthesitis
☒ physician global
☒ BASMI
☒ other

Please specify: BASDAI, BASFI

Time points for registration

	at inclusion in registry	at start/change of treatment	at follow-up visits	other
Swollen joint count	☒	☒	☒	☐
Tender joint count	☒	☒	☒	☐
Enthesitis	☒	☒	☒	☐
Physician global	☒	☒	☒	☐

Swollen joints

How many swollen joint counts can be registered?
(Tick all that apply)

☒ 66
☒ other

Please specify: 79 optional

Do you register specific location of swollen joints?
(Tick all that apply)

☒ yes

Tender joints

How many tender joint counts can be registered?
(Tick all that apply)

☒ 68
☒ other

Please specify: 79 optional

Do you register specific location of tender joints?
(Tick all that apply)

☒ yes

Enthesitis

Do you register specific location of enthesitis? (Tick all that apply)	☒ yes
Which locations are registered? (Tick all that apply)	☒ medial femoral condyle<br>☒ achilles tendon insertion<br>☒ lateral epicondyle<br>☒ 1st costochondral joint<br>☒ 7th costochondral joint<br>☒ posterior superior iliac spine<br>☒ anterior superior iliac spine<br>☒ iliac crests<br>☒ 5th lumbar spinous process
Please indicate which specific indices that are used, if applicable	MASES, LEI

Physician Global

Please write the wording of the question regarding physician global	physicians evaluatation of activity of disease (translate into english if possible)

BASMI

Are the individidual BASMI components registered?	⊗ yes
Do you register the individual BASMI measurements (cm, degrees)?	⊗ yes

Coxitis

Do you assess coxitis? If yes, please indicate how it is assessed	no
General comment	Schober, chest-expansion, forward-lateral bends, head to wall measures, head rotations

Response was added on 2021-12-14 17:10:25.

Psoriatic arthritis

Which disease status characteristics can be registered in PsA patients? (Tick all that apply)	☒ swollen joint count<br>☒ tender joint count<br>☒ enthesitis<br>☒ skin<br>☒ physician Global

Time points for registration

	at inclusion in registry	at start/change of treatment	at follow-up visits	other
Swollen joint count	☒	☒	☒	☐
Tender joint count	☒	☒	☒	☐
Enthesitis	☒	☒	☒	☐
Skin	☒	☒	☒	☐
Physician Global	☒	☒	☒	☐

Swollen joints

How many swollen joint counts can be registered? (Tick all that apply)	☒ 66<br>☒ Other
Please specify	79 optional
Do you register specific location of swollen joints? (Tick all that apply)	☒ yes

Tender joints

How many tender joint counts can be registered? (Tick all that apply)	☒ 68<br>☒ Other
Please specify	79 optional
Do you register specific location of tender joints? (Tick all that apply)	☒ yes

Entesitis

Do you register specific location of enthesitis? (Tick all that apply)	☒ yes
Which locations are registered? (Tick all that apply)	☒ medial femoral condyle<br>☒ achilles tendon insertion<br>☒ lateral epicondyle<br>☒ greater tochanter<br>☒ 1st costochondral joint<br>☒ 7th costochondral joint<br>☒ posterior superior iliac spine<br>☒ anterior superior iliac spine<br>☒ iliac crests<br>☒ 5th lumbar spinous process
Please indicate which specific indices that are used, if applicable	MASES, LEI

Skin

Which instruments are used for registering skin involvment in PsA? (Tick all that apply)	☒ body surface area %

Physician global

Please write the wording of the question regarding physician global	physicians evaluation of disease activity (Translate into english if possible)

Coxitis

Do you assess coxitis? If yes, please indicate how it is assessed	no
General comment	head to wall, forward-lateral bends, chest expansion, Schober, if applicable/symphtoms

Response was added on 2021-12-14 17:18:11.

Medication

Which therapies are registered in your registry? (Tick all that apply)	☒ biological dmards (bDMARDs), including targeted synthetic dmards (JAK)<br>☒ conventional synthetic dmards (csDMARDs)<br>☒ glucocorticoids

bDMARDs

Which year did bDMARD registration begin?	2000
Is it mandatory to register bDMARD therapy?	⊗ no

csDMARDs

Which year did csDMARD registration begin?	2008
Is it mandatory to register csDMARD therapy?	⊗ no

Glucocorticoids

Which year did glucocorticoid registration begin?	2000
Is it mandatory to register glucocorticoid therapy?	⊗ no
Is the mode of administration registered? (Tick all that apply)	☒ oral

What information regarding ONGOING medications is registered?

	start date	stop date	temporary start and stop dates	discontinuation reasons	dosage	frequency	administration mode
bDMARDs	☒	☒	☐	☒	☒	☒	☐
csDMARDs	☒	☒	☐	☒	☒	☒	☐
glucocorticoids	☒	☒	☐	☐	☒	☒	☐

Time points for registration (ONGOING medications)

	at inclusion in registry	at start/change of treatment	at follow-up visits	other
bDMARDs	☒	☒	☒	☐
csDMARDs	☒	☒	☒	☐
glucocorticoids	☒	☒	☒	☐

What information regarding PAST medications is registered at inclusion in registry?

	start date	stop date	temporary start and stop dates	discontinuation reasons	dosage	name of drug
bDMARDs	☒	☒	☐	☒	☒	☒
csDMARDs	☒	☒	☐	☒	☒	☒
glucocorticoids	☒	☒	☐	☐	☒	☒

Discontinuation reasons

What are the possible reasons for discontinuation of a bDMARD?<br>(Tick all that apply)	☒ lack of efficacy<br>☒ adverse events<br>☒ remission<br>☒ other
Please specify	SAE, skinreaction, bloodreaction
Is it possible to register multiple reasons for discontinuation?	⊗ no
Does your registry link to prescription database or other external data sources on prescriptions on a regular basis as input to registry?<br>(Tick all that apply)	☒ yes, for bDMARDS<br>☒ yes, for csDMARDS<br>☒ yes, for glucocorticoids

Response was added on 2021-12-14 17:32:19.

Patient reported outcomes

Which patient reported outcomes (PROs) are registered in axSpA and/or PsA patients? (Tick all that apply)	☒ BASDAI<br>☒ BASFI<br>☒ pain<br>☒ fatigue<br>☒ global disease<br>☒ HAQ<br>☒ other
Please specify	15D equaling EQ-5D
Are any of the PROs registered in either axSpA or PsA only?	⊗ no, all PROs are registered in both diagnoses

Mode of registration

What are the options for registration of PROs? (Tick all that apply)	☒ on screen in waiting room<br>☒ through website/app

Time points for registration

	at inclusion in registry	at start/change of treatment	at follow-up visits	other
BASDAI	☒	☒	☒	☐
BASFI	☒	☒	☒	☐
Pain	☒	☒	☒	☐
Fatigue	☒	☒	☒	☐
Global disease	☒	☒	☒	☐
HAQ	☒	☒	☒	☐

BASDAI

Are the individidual BASDAI components registered?	⊗ yes

BASFI

Are the individidual BASFI components registered?	⊗ yes

Pain, fatigue and global assessments

Please write the wording of the question relating to pain	how much pain have you been experiencing<br>(please translate into english if possible)
Please write the wording of the question relating to fatigue	how much fatigue have you been experiencing during last week<br>(please translate into english if possible)
Please write the wording of the question relating to global assessment of disease	how are you doing regaring your joint disease<br>(please translate into english if possible)

HAQ

Are the individual HAQ items registered?	⊗ yes
Which HAQ versions may be used in your registry?<br>(Tick all that apply)	☒ stanford HAQ DI with adjustment for use of aids and devices

Response was added on 2021-12-14 17:34:18.

Laboratory

Which laboratory test can be registered? (Tick all that apply)	☒ CRP<br>☒ HLA-B27<br>☒ IgM-RF

	at inclusion in registry	at start/change of treatment	at follow-up visits	other
CRP	☒	☒	☒	☐

How are laboratory test results registered? (Tick all that apply)	☒ electronically tranfered from lab system<br>☒ entered by health care staff
General comments	CCP-ab

Response was added on 2021-12-14 17:35:16.

General comments	mention of SI.tis either on X-ray or MRI

Response was added on 2021-12-14 17:38:03.

Comorbidities

Which extraarticular manifestations and comorbid conditions are registered? (Tick all that apply)

☒ uveitis
☒ psoriasis
☒ inflammatory bowel disease (IBD)
☒ chronic liver disease, eg. hepatitis, cirrhosis
☒ tuberculosis (TB)

Time points for registration

	at inclusion in registry	at start/change of treatment	at follow-up visits	other
uveitis	☒	☐	☐	☐
psoriasis	☒	☐	☐	☐
IBD	☒	☐	☐	☐
chronic liver disease	☒	☐	☐	☒
TB	☒	☐	☐	☒

Please specify (chronic liver disease): evaluated on demand

Please specify (TB): evaluated on demand

Mode of registration - how are comorbid conditions registered?

	patient-reported	by health-staff	linkage from other registry
uveitis	☐	☒	☐
psoriasis	☐	☒	☐
IBD	☐	☒	☐
chronic liver disease	☐	☒	☐
TB	☐	☒	☐

Do you use ICD-10 codes for registration? ⊗ yes

General comments: other co-morbidities might be manually entered if relevant

Response was added on 2021-12-14 17:41:23.

Lifestyle

Which lifestyle parameters are registered? (Tick all that apply)	☒ smoking

Time points for registration

	at inclusion in registry	at start/change of treatment	at follow-up visits	other
Smoking	☒	☒	☐	☐

Smoking

How is smoking status characterised? (Tick all that apply)	☒ current<br>☒ former<br>☒ never
Do you register a start date? (Tick all that apply)	☒ for current smokers<br>☒ for former smokers
Do you register a stop date for former smokers?	⊗ yes
How is average number of smoked cigarettes registered? (Tick all that apply)	☒ not registered

Response was added on 2021-12-14 17:43:28.

Safety

Can you register adverse events in your registry? (Tick all that apply)	☒ yes, directly into registry
Is it mandatory to register adverse events through your registry?	⊗ no
Which adverse events are registered in your registry? (Tick all that apply)	☒ non-serious adverse events<br>☒ serious adverse events?<br>☒ other
Other	all relevant

Information on adverse events

	date of event	MeddRA	ICD10-code	outcome	other
Non serious AE	☒	☐	☐	☒	☐
Serious AE	☒	☐	☐	☒	☐

Participant information

Record ID	7
Name	Tore Kvien
Registry	NOR-DMARD
E-mail	t.k.a.kvien@medisin.uio.no
Deltager	⊗ ja

Response was added on 2021-12-08 14:06:08.

General registry information

What is the status of your registry?	⊗ running and including patients

Coverage

Please estimate how many (percentage) of the eligible spondyloarthritis patients in your country, that are registered	25
Please estimate how many (percentage) of the eligible psoriatic arthritis patients in your country, that are registered	25
How did you obtain the coverage estimate above?<br>(Tick all that apply)	☒ other
Please specify	Based on population
Which institutions/organisations can include patients in your registry?<br>(Tick all that apply)	☒ departments of rheumatology at hospitals
Please give an estimate of how many departments of rheumatology at hospitals (not including university hospitals) that include patients in your registry	4
When is the data registered?<br>(Tick all that apply)	☒ at pre-specified registry visits
What is the schedule for pre-specified registry visits?	0, 3, 6, 9, 12, 18, 24 - then every 6 months
Is it possible to register visits outside of the pre-specified visit schedule, eg. if a patient has a flare?	⊗ no

Ethics

Is approval from a local ethics committee needed for a study on de-identified data (eg. a EuroSpA upload)?<br>(Tick all that apply)	☒ comment
Comment	The informed consent includes information about data sharing

Do patients need to sign informed consent to be included in your registry? (Tick all that apply)	☒ yes
Are any additional local approvals needed for a study on de-identified data (eg. a EuroSpA upload)? (Tick all that apply)	☒ no

Funding

How is the registry funded? (Tick all that apply)	☒ from research grants<br>☒ industry
Please estimate the percentage of funds coming from research grants	20<br>((0-100%))
Please estimate the percentage of funds coming from industry, eg. pharmaceutical company	80<br>((0-100%))
The percentages add correctly up to a 100%	
What is the basis of participation by a clinic/department/office in the registry? (Tick all that apply)	☒ voluntary
Is the clinic/department/office financially compensated for registration?	⊗ no

Inclusion criteria

What event triggers the inclusion of a patient into the registry? (Tick all that apply)	☒ new treatment
Which criteria do you base the inclusion on? (Tick all that apply)	☒ diagnosis<br>☒ treatment
Is a minimum age required for inclusion?	☒ yes, 18 years or above
Which diagnoses are included in your registry?<br><br>(Tick all that apply)	☒ ankylosing spondylitis (AS)<br>☒ non-radiographic axial spondyloarthritis (nr-axSpA)<br>☒ psoriatic arthritis (PsA)<br>☒ rheumatoid arthritis<br>☒ other
Which year did inclusion of AS patients begin?	2000
Which year did inclusion of nr-axSpA patients begin?	2010
Which year did inclusion of PsA patients begin?	2000
Which other diagnoses are included?	All arthritides receiving csDMARDs (until 2012) and bDMARDs (from 2000)ed

Do patients need to be treated with biological DMARDs (including targeted synthetic DMARDs) to be included in the registry?	⊗ yes
Have the inclusion criteria changed over time?	⊗ yes
How and when did inclusion criteria change?	All DMARDs from 2000, only bDMARDs and tsDMARDs from 2012

Response was added on 2021-12-08 14:09:57.

Data management

What are the options for data entry?<br>(Tick all that apply)	☒ electronic
Are the data fields in your registry interactive, such that invalid or unprobable data is flagged when entered (edit checks)<br>(Tick all that apply)	☒ comment
Comment	Interactive to some extent
Please describe any other data validation procedures that you may use	Summary statistics
How does your registry retain data management services?<br>(Tick all that apply)	☒ person employed as data manager
Which is the background of your data manager(s)?<br>(Tick all that apply)	☒ data science/biostatistician/technical
Which are the most commonly used data analysis software/programming languages in your organization?<br>(Tick all that apply)	☒ R<br>☒ stata<br>☒ SPSS
How is the data stored in the registry?<br>(Tick all that apply)	☒ a relational database framework (like SQL)
Which are the main data formats used for raw data extractions?<br>(Tick all that apply)	☒ excel
Where is your data collection platform hosted?<br>(Tick all that apply)	☒ at a hopital in a rheumatology department
Who maintains your data collection platform?<br>(Tick all that apply)	☒ data manager
How frequently is the database updated with the latest information?<br>(Tick all that apply)	☒ monthly
Is linkage to other databases or registries possible?	⊗ yes
Which registries can be linked to?<br>(Tick all that apply)	☒ mortality registry<br>☒ prescription registry<br>☒ comorbidity

Response was added on 2021-12-08 14:12:34.

Demography

Please indicate which of the following variables are collected in your registry (Tick all that apply)	☒ age (year of birth)<br>☒ sex<br>☒ weight<br>☒ height

Time points for registration

	at inclusion in registry	at start/change of treatment	at follow-up visits	other
Weight	☐	☐	☐	☒
Height	☐	☐	☐	☒

Please specify (weight)	To be included with next update in protocol
Please specify (height)	To be included with next update in protocol

Diagnosis

How is a diagnosis registered? (Tick all that apply)	☒ through ICD-10 codes
Do you register (Tick all that apply)	☒ date for diagnosis
Do you register (Tick all that apply)	☒ not registered

Time points for registration

	at inclusion in registry	at start/change of treatment	at follow-up visits	other
Day/month/year of diagnosis	☒	☐	☐	☐

Which classification criteria are registered? (Tick all that apply)	☒ ASAS<br>☒ CASPAR

Time points for registration

	at inclusion in registry	at start/change of treatment	at follow-up visits	other
ASAS	☒	☒	☐	☐
CASPAR	☒	☒	☐	☐

In which patients can you register ASAS?	⊗ AxSpA
Do you register individual ASAS classification items?<br>(Tick all that apply)	☒ yes
Do you register individual CASPAR classification items?<br>(Tick all that apply)	☒ yes

Response was added on 2021-12-08 14:20:30.

Axial spondyloarthritis

Which disease status characteristics can be registered in axSpA patients?<br>(Tick all that apply)	☒ swollen joint count<br>☒ tender joint count<br>☒ physician global<br>☒ other
Please specify	BASDAI and ASDAS

Time points for registration

	at inclusion in registry	at start/change of treatment	at follow-up visits	other
Swollen joint count	☒	☒	☒	☐
Tender joint count	☒	☒	☒	☐
Physician global	☒	☒	☒	☐

Swollen joints

How many swollen joint counts can be registered?<br>(Tick all that apply)	☒ other
Please specify	28 plus 4 (foot and ankle)
Do you register specific location of swollen joints?<br>(Tick all that apply)	☒ yes

Tender joints

How many tender joint counts can be registered?<br>(Tick all that apply)	☒ other
Please specify	same as for swollen joints
Do you register specific location of tender joints?<br>(Tick all that apply)	☒ yes

Physician Global

Please write the wording of the question regarding physician global	Hvordan vil du beskrive pasientens sykdomsaktivitet? VAS 0-100 fra "ingen aktivitet" til "svært aktiv"<br>(translate into english if possible)

Coxitis

Do you assess coxitis? If yes, please indicate how it is assessed	No

Response was added on 2021-12-08 14:22:10.

Psoriatic arthritis

Which disease status characteristics can be registered in PsA patients? (Tick all that apply)	☒ swollen joint count<br>☒ tender joint count<br>☒ physician Global<br>☒ other
Please specify	DAPSA based on 28 joints

Time points for registration

	at inclusion in registry	at start/change of treatment	at follow-up visits	other
Swollen joint count	☒	☒	☒	☐
Tender joint count	☒	☒	☒	☐
Physician Global	☒	☒	☒	☐

Swollen joints

How many swollen joint counts can be registered? (Tick all that apply)	☒ Other
Please specify	28 + 4 as for AS
Do you register specific location of swollen joints? (Tick all that apply)	☒ yes

Tender joints

How many tender joint counts can be registered? (Tick all that apply)	☒ Other
Please specify	28 + 4
Do you register specific location of tender joints? (Tick all that apply)	☒ yes

Physician global

Please write the wording of the question regarding physician global	Hvordan vil du beskrive pasientens sykdomsaktivitet? VAS 0-100 fra "ingen aktivitet" til "svært aktiv"<br>(Translate into english if possible)

Coxitis

Do you assess coxitis? If yes, please indicate how it is assessed	No

Response was added on 2021-12-08 14:25:47.

Medication

Which therapies are registered in your registry? (Tick all that apply)	☒ biological dmards (bDMARDs), including targeted synthetic dmards (JAK)<br>☒ conventional synthetic dmards (csDMARDs)<br>☒ glucocorticoids

bDMARDs

Which year did bDMARD registration begin?	2000
Is it mandatory to register bDMARD therapy?	⊗ other
Please specify	In principle yes, but some patients do not approve participation and some clinicians forget

csDMARDs

Which year did csDMARD registration begin?	2000
Is it mandatory to register csDMARD therapy?	⊗ other
Please specify	since 2012 only registered when in combination with bdmard
Is it mandatory to register glucocorticoid therapy?	⊗ no
Is the mode of administration registered? (Tick all that apply)	☒ oral<br>☒ intramuscular<br>☒ intraarticular
Are specific locations of injected joints registered?	⊗ yes

What information regarding ONGOING medications is registered?

	start date	stop date	temporary start and stop dates	discontinuation reasons	dosage	frequency	administration mode
bDMARDs	☒	☒	☐	☒	☒	☒	☐
csDMARDs	☒	☒	☐	☐	☒	☒	☐
glucocorticoids	☒	☒	☐	☐	☒	☒	☒

Time points for registration (ONGOING medications)

	at inclusion in registry	at start/change of treatment	at follow-up visits	other
bDMARDs	☒	☒	☒	☐
csDMARDs	☒	☒	☒	☐
glucocorticoids	☒	☒	☒	☐

What information regarding PAST medications is registered at inclusion in registry?

	start date	stop date	temporary start and stop dates	discontinuation reasons	dosage	name of drug
bDMARDs	☒	☒	☐	☐	☐	☒
csDMARDs	☐	☐	☐	☐	☐	☒
glucocorticoids	☐	☐	☐	☐	☐	☒

Discontinuation reasons

What are the possible reasons for discontinuation of a bDMARD?<br>(Tick all that apply)	☒ lack of efficacy<br>☒ adverse events<br>☒ remission<br>☒ lost to follow-up<br>☒ death<br>☒ other
Please specify	Specified as text
Is it possible to register multiple reasons for discontinuation?	⊗ yes
How is it decided which is the primary reason for discontinuation?	Uncertain
Does your registry link to prescription database or other external data sources on prescriptions on a regular basis as input to registry?<br>(Tick all that apply)	☒ no
General comments	Linkage to prescriptions can be done for validation purposes

Response was added on 2021-12-08 14:28:39.

Patient reported outcomes

Which patient reported outcomes (PROs) are registered in axSpA and/or PsA patients? (Tick all that apply)	☒ BASDAI<br>☒ pain<br>☒ fatigue<br>☒ global disease<br>☒ EQ-5D<br>☒ patient acceptable symptom state (PASS)<br>☒ other
Please specify	MHAQ
Are any of the PROs registered in either axSpA or PsA only?	⊗ no, all PROs are registered in both diagnoses

Mode of registration

What are the options for registration of PROs? (Tick all that apply)	☒ on screen in waiting room<br>☒ through website/app

Time points for registration

	at inclusion in registry	at start/change of treatment	at follow-up visits	other
BASDAI	☒	☒	☒	☐
Pain	☒	☒	☒	☐
Fatigue	☒	☒	☒	☐
Global disease	☒	☒	☒	☐
EQ-5D	☒	☒	☒	☐
PASS	☒	☒	☒	☐

BASDAI

Are the individidual BASDAI components registered?	⊗ yes

Pain, fatigue and global assessments

Please write the wording of the question relating to pain	"Hvor mye smerte har du hatt den siste uken?" VAS fra "Ingen smerter" til "Uutholdelige smerter"<br>(please translate into english if possible)
Please write the wording of the question relating to fatigue	"I hvilken grad har en følelse av uvanlig tretthet eller utmattelse vært et problem for deg den siste uken?" VAS fra "Tretthet er ikke noe problem" til "Tretthet er et stort problem"<br>(please translate into english if possible)

Please write the wording of the question relating to global assessment of disease	"Vi ber deg vennligst vurdere aktiviteten i din revmatiske sykdom i løpet av den siste uken. Når du tar alle symptomene med i betraktning, hvordan synes du tilstanden er?" VAS fra "Bra, ingen symptomer" til "Svært dårlig"<br>(please translate into english if possible)

EQ-5D

Are the individual EQ-5D items registered?	⊗ yes
Which EQ-5D version is used?	3 response options
Which algorithm is used?	I think it is the British

Patient acceptable symptom state (PASS)

Please write the wording of the question relating to PASS	Tenk gjennom hvordan din revmatiske sykdom har påvirket deg den siste uken. Hvis du de neste månedene vil forbli som du var i løpet av den siste uken, vil det være akseptabelt eller uakseptabelt for deg? (Akseptabelt - Uakseptabelt)<br>(please translate into english if possible)
General comments	PASS udgår i forbindelse med næste protokol opdatering.

Response was added on 2021-12-08 14:29:23.

Laboratory

Which laboratory test can be registered? (Tick all that apply)

☒ ESR
☒ CRP
☒ HLA-B27
☒ IgM-RF
☒ stored samples available for later analyses (biobank)

Time points for registration

	at inclusion in registry	at start/change of treatment	at follow-up visits	other
ESR	☒	☒	☒	☐
CRP	☒	☒	☒	☐
Stored samples available for later analyses (biobank)	☒	☒	☒	☐

Biobank

Which sample types are collected? (Tick all that apply)

☒ whole-blood
☒ serum

How are laboratory test results registered? (Tick all that apply)

☒ entered by health care staff

Response was added on 2021-12-08 14:30:05.

Frequency of image registration

Please comment on the frequency of registration of the various image modalities, eg. at specified time points, as needed, in connection with research projects or other.	Not entered into NOR-DMARD

Response was added on 2021-12-08 14:31:13.

Comorbidities

Which extraarticular manifestations and comorbid conditions are registered? (Tick all that apply)	☒ ischemic heart disease (IHD)<br>☒ cerebrovascular disease (CVD)<br>☒ hypertension<br>☒ diabetes (DM)<br>☒ chronic kidney insufficiency (CKI)<br>☒ chronic liver disease, eg. hepatitis, cirrhosis<br>☒ solid cancer<br>☒ hematological cancer<br>☒ depression<br>☒ other
Please specify	hypertension, angina pectoris, myocardial infarction, cardiac failure, previous percutaneous coronary intervention (PCI) or bypass operation, intermittent claudication, cerebral haemorrhage/ischemic stroke/transient ischemic attack (TIA), asthma/bronchitis/chronic lung disease, allergy/eczema, chronic back pain, cancer, neurologic disease, diabetes, hypothyroidism, mental illness, alcohol and/or narcotic substance abuse, renal disease, liver disease, gastrointestinal disease including ulcer, anaemia or other haematological disorder, and about occurrence of early coronary vascular disease (CVD)

	at inclusion in registry	at start/change of treatment	at follow-up visits	other
IHD	☒	☐	☒	☐
CVD	☒	☐	☒	☐
hypertension	☒	☐	☒	☐
DM	☒	☐	☒	☐
CKI	☒	☐	☒	☐
chronic liver disease	☒	☐	☒	☐
solid cancer	☒	☐	☒	☐
hematological cancer	☒	☐	☒	☐
depression	☒	☐	☒	☐
other	☒	☐	☒	☐

	patient-reported	by health-staff	linkage from other registry
IHD	☒	☐	☐
CVD	☒	☐	☐
hypertension	☒	☐	☐

DM	☒	☐	☐
CKI	☒	☐	☐
chronic liver disease	☒	☐	☐
solid cancer	☒	☐	☐
hematological cancer	☒	☐	☐
depression	☒	☐	☐
other	☒	☐	☐

Do you use ICD-10 codes for registration?	⊗ no
General comments	Via go-treat it

Response was added on 2021-12-08 14:31:47.

Lifestyle

Which lifestyle parameters are registered?
(Tick all that apply)

☒ smoking

Time points for registration

	at inclusion in registry	at start/change of treatment	at follow-up visits	other
Smoking	☒	☐	☐	☐

Smoking

How is smoking status characterised?
(Tick all that apply)

☒ current
☒ former
☒ never

Do you register a start date?
(Tick all that apply)

☒ no date is registered

Do you register a stop date for former smokers?

⊗ No

How is average number of smoked cigarettes registered?
(Tick all that apply)

☒ not registered

Response was added on 2021-12-08 14:32:28.

Safety

Can you register adverse events in your registry? (Tick all that apply)	☒ yes, directly into registry
Is it mandatory to register adverse events through your registry?	⊗ yes
Which adverse events are registered in your registry? (Tick all that apply)	☒ non-serious adverse events<br>☒ serious adverse events?

Information on adverse events

	date of event	MeddRA	ICD10-code	outcome	other
Non serious AE	☒	☒	☐	☐	☐
Serious AE	☒	☒	☐	☐	☐

General comments	With next protocol update, only SAEs will be recorded.

Participant information

Record ID	8
Name	Pasoon Hellamand
Registry	ARC
E-mail	p.hellamand@amsterdamumc.nl
Deltager	⊗ ja

Response was added on 2022-01-11 13:08:22.

General registry information

What is the status of your registry?	⊗ running, but closed for inclusion

Coverage

Please estimate how many (percentage) of the eligible spondyloarthritis patients in your country, that are registered	0
Please estimate how many (percentage) of the eligible psoriatic arthritis patients in your country, that are registered	0
How did you obtain the coverage estimate above? (Tick all that apply)	☒ other
Please specify	One study (van der linden, 1984) has found a prevalence of 0.10% for AS patients in the NL. Assuming 17.5 million population, this amounts to 17500 AS patients. Calculation ((100/17500)*100%) = 0.5%The amount of patients with AS were ~3000 (=2957).
Which institutions/organisations can include patients in your registry? (Tick all that apply)	☒ departments of rheumatology at university hospitals
Please give an estimate of how many departments of rheumatology at university hospitals that include patients in your registry	1
When is the data registered? (Tick all that apply)	☒ at routine visits
Are all routine visits registered in your registry - or only some?	⊗ all visits

Ethics

Is approval from a local ethics committee needed for a study on de-identified data (eg. a EuroSpA upload)? (Tick all that apply)	☒ yes<br>☒ comment
Comment	We added in the PIF an option to give permission to share data with researchers abroad. So only patients that have ticked this option are uploaded to EuroSpA.

Do patients need to sign informed consent to be included in your registry?<br>(Tick all that apply)	☒ yes
Are any additional local approvals needed for a study on de-identified data (eg. a EuroSpA upload)?<br>(Tick all that apply)	☒ no
What is the basis of participation by a clinic/department/office in the registry?<br>(Tick all that apply)	☒ voluntary
Is the clinic/department/office financially compensated for registration?	⊗ no

Inclusion criteria

What event triggers the inclusion of a patient into the registry?<br>(Tick all that apply)	☒ other
Please specify	In our registry any patient with axSpA regardless of treatment stage can be included in the registry. We have an outpatient clinic where all patients who had a visited were asked to join the study.
Which criteria do you base the inclusion on?<br>(Tick all that apply)	☒ diagnosis
Is a minimum age required for inclusion?	☒ yes, 18 years or above
Which diagnoses are included in your registry?<br><br>(Tick all that apply)	☒ ankylosing spondylitis (AS)<br>☒ non-radiographic axial spondyloarthritis (nr-axSpA)
Which year did inclusion of AS patients begin?	2019
Which year did inclusion of nr-axSpA patients begin?	2019
Have the inclusion criteria changed over time?	⊗ no
General comments	The answer to question one is 0.5% but it does not let me answer this.

Response was added on 2022-01-11 11:14:00.

Data management

What are the options for data entry? (Tick all that apply)	☒ paper based<br>☒ electronic
Are the data fields in your registry interactive, such that invalid or unprobable data is flagged when entered (edit checks) (Tick all that apply)	☒ yes
Since when (year) has the data fields been interactive?	2019
How does your registry retain data management services? (Tick all that apply)	☒ a researcher/administrative personal does data management beside other duties
Which is the background of your data manager(s)? (Tick all that apply)	☒ clinical
Which are the most commonly used data analysis software/programming languages in your organization? (Tick all that apply)	☒ R<br>☒ other
Please specify	Google spreadsheet, I don't use excel.
How is the data stored in the registry? (Tick all that apply)	☒ a relational database framework (like SQL)
Which are the main data formats used for raw data extractions? (Tick all that apply)	☒ simple text files, eg. csv, tsv
Where is your data collection platform hosted? (Tick all that apply)	☒ external company
Who maintains your data collection platform? (Tick all that apply)	☒ data manager<br>☒ researcher/epidemiologist
How frequently is the database updated with the latest information? (Tick all that apply)	☒ weekly<br>☒ monthly
Is linkage to other databases or registries possible?	⊗ no

Response was added on 2022-01-11 11:18:49.

Demography

Please indicate which of the following variables are collected in your registry
(Tick all that apply)

- ☒ age (year of birth)
- ☒ sex
- ☒ weight
- ☒ height
- ☒ death

Time points for registration

	at inclusion in registry	at start/change of treatment	at follow-up visits	other
Weight	☒	☒	☐	☐
Height	☒	☒	☐	☐

How is vital status registered
(Tick all that apply)

- ☒ by healthstaff, manually

Diagnosis

How is a diagnosis registered?
(Tick all that apply)

- ☒ other diagnostic categories

Specify which diagnostic categories

Diagnosis is made by the treating rheumatologist.
It does not always have to be ASAS+ and/or NY+, but usually it is.

Do you register
(Tick all that apply)

- ☒ date for diagnosis
- ☒ month of diagnosis
- ☒ year of diagnosis

Do you register
(Tick all that apply)

- ☒ year of symptom onset

Time points for registration

	at inclusion in registry	at start/change of treatment	at follow-up visits	other
Day/month/year of diagnosis	☒	☐	☐	☐
Day/month/year of symptom onset	☒	☐	☐	☐

Which classification criteria are registered?
(Tick all that apply)

- ☒ ASAS
- ☒ New York

Time points for registration

	at inclusion in registry	at start/change of treatment	at follow-up visits	other
ASAS	☒	☐	☐	☐
New York	☒	☐	☐	☐

In which patients can you register ASAS?	⊗ AxSpA
Do you register individual ASAS classification items? (Tick all that apply)	☒ yes
Do you register individual New York classification items? (Tick all that apply)	☒ no

Response was added on 2022-01-11 11:32:50.

Axial spondyloarthritis

Which disease status characteristics can be registered in axSpA patients?
(Tick all that apply)

☒ swollen joint count
☒ tender joint count
☒ enthesitis
☒ physician global
☒ BASMI

Time points for registration

	at inclusion in registry	at start/change of treatment	at follow-up visits	other
Swollen joint count	☒	☒	☒	☐
Tender joint count	☒	☒	☒	☐
Enthesitis	☒	☒	☒	☐
Physician global	☒	☒	☒	☐
BASMI	☒	☒	☒	☐

Swollen joints

How many swollen joint counts can be registered?
(Tick all that apply)

☒ 28
☒ 44

Do you register specific location of swollen joints?
(Tick all that apply)

☒ yes

Tender joints

How many tender joint counts can be registered?
(Tick all that apply)

☒ 28
☒ 44

Do you register specific location of tender joints?
(Tick all that apply)

☒ yes

Enthesitis

Do you register specific location of enthesitis?
(Tick all that apply)

☒ yes

Which locations are registered?
(Tick all that apply)

☒ achilles tendon insertion
☒ 1st costochondral joint
☒ 7th costochondral joint
☒ posterior superior iliac spine
☒ anterior superior iliac spine
☒ iliac crests
☒ 5th lumbar spinous process

Please indicate which specific indices that are used, if applicable	MASES criteria

Physician Global

Please write the wording of the question regarding physician global	How active was your disease on average during the last week (scale 0-10); 0 = not active; 10 = very active<br>(translate into english if possible)

BASMI

Are the individidual BASMI components registered?	⊗ yes
Do you register the individual BASMI measurements (cm, degrees)?	⊗ yes
Which BASMI scale is used?	⊗ 3-point

Coxitis

Do you assess coxitis? If yes, please indicate how it is assessed	Only if the patient has complaints, not regularly.
General comment	For the BASMI we have the continuous values (so we could calculate linear) and we have the 3-point scale.

Response was added on 2022-01-11 11:33:03.

General comment	We do not include PsA patients.

Response was added on 2022-01-11 11:36:37.

Medication

Which therapies are registered in your registry? (Tick all that apply)	☒ biological dmards (bDMARDs), including targeted synthetic dmards (JAK)<br>☒ conventional synthetic dmards (csDMARDs)<br>☒ glucocorticoids<br>☒ NSAIDs<br>☒ medication for comorbidity<br>☒ other
Please specify	all medications are registered

bDMARDs

Which year did bDMARD registration begin?	2019
Is it mandatory to register bDMARD therapy?	⊗ yes

csDMARDs

Which year did csDMARD registration begin?	2019
Is it mandatory to register csDMARD therapy?	⊗ no

Glucocorticoids

Which year did glucocorticoid registration begin?	2019
Is it mandatory to register glucocorticoid therapy?	⊗ no
Is the mode of administration registered? (Tick all that apply)	☒ oral

NSAIDs

Which year did NSAID registration begin?	2019
Is it mandatory to register NSAID therapy?	⊗ no

Medicaton for comorbidities

What types of medical therapy for comorbidities are registered?	all

What information regarding ONGOING medications is registered?

	start date	stop date	temporary start and stop dates	discontinuation reasons	dosage	frequency	administration mode
bDMARDs	☒	☒	☒	☒	☒	☒	☐
csDMARDs	☐	☐	☐	☐	☒	☒	☐
glucocorticoids	☐	☐	☐	☐	☒	☒	☐
NSAIDs	☐	☐	☐	☐	☒	☒	☐
medication for comorbidity	☐	☐	☐	☐	☒	☒	☐

Time points for registration (ONGOING medications)

	at inclusion in registry	at start/change of treatment	at follow-up visits	other
bDMARDs	☒	☒	☒	☐
csDMARDs	☒	☒	☒	☐
glucocorticoids	☒	☒	☒	☐
NSAIDs	☒	☒	☒	☐
medication for comorbidity	☒	☒	☒	☐

What information regarding PAST medications is registered at inclusion in registry?

	start date	stop date	temporary start and stop dates	discontinuation reasons	dosage	name of drug
bDMARDs	☒	☒	☒	☒	☒	☒

Discontinuation reasons

What are the possible reasons for discontinuation of a bDMARD?<br>(Tick all that apply)	☒ lack of efficacy<br>☒ adverse events<br>☒ remission<br>☒ pregnancy wish<br>☒ death
Is it possible to register multiple reasons for discontinuation?	⊗ no
Does your registry link to prescription database or other external data sources on prescriptions on a regular basis as input to registry?<br>(Tick all that apply)	☒ no
General comments	Theoretically speaking we could easily get start & stop dates for all patients, however, we do not register them in our EDBC. Just for bDMARDs.

Response was added on 2022-01-11 12:44:54.

Patient reported outcomes

Which patient reported outcomes (PROs) are registered in axSpA and/or PsA patients? (Tick all that apply)	☒ BASDAI<br>☒ BASFI<br>☒ nightly pain<br>☒ fatigue<br>☒ global disease<br>☒ EQ-5D<br>☒ other
Please specify	General health questionaaire, physical activity, intoxications,
Are any of the PROs registered in either axSpA or PsA only?	⊗ yes
Which PROs are registered uniquely in either axSpA or PsA?	all in axspa

Mode of registration

What are the options for registration of PROs? (Tick all that apply)	☒ paper forms

Time points for registration

	at inclusion in registry	at start/change of treatment	at follow-up visits	other
BASDAI	☒	☒	☒	☐
BASFI	☒	☒	☒	☐
Fatigue	☒	☒	☒	☐
Global disease	☒	☒	☒	☐
EQ-5D	☒	☒	☒	☐

BASDAI

Are the individidual BASDAI components registered?	⊗ yes

BASFI

Are the individidual BASFI components registered?	⊗ yes
Please write the wording of the question relating to nightly pain	Howmuch backpain did you have during the night the last week?<br>(please translate into english if possible)
Please write the wording of the question relating to fatigue	How tired were you the last week?<br>(please translate into english if possible)
Please write the wording of the question relating to global assessment of disease	How active was your disease the last weeks?<br>(please translate into english if possible)

EQ-5D

Are the individual EQ-5D items registered?	⊗ yes
Which EQ-5D version is used?	The one with 5 options
Which algorithm is used?	I haven't created a composite score yet
General comments	We also have a global assessment of health:<br><br>To what extent did your disease affect your global health during the last week (also available last 6 months).

Response was added on 2022-01-11 12:47:43.

Laboratory

Which laboratory test can be registered? (Tick all that apply)	☒ ESR<br>☒ CRP<br>☒ hemoglobin<br>☒ ALAT<br>☒ creatinine<br>☒ HLA-B27

Time points for registration

	at inclusion in registry	at start/change of treatment	at follow-up visits	other
ESR	☒	☒	☒	☐
CRP	☒	☒	☒	☐
Hemoglobin	☒	☒	☒	☐
ALAT	☒	☒	☒	☐
Creatinine	☒	☒	☒	☐

How are laboratory test results registered? (Tick all that apply)	☒ entered by health care staff
General comments	We also have AF, gGT, thrombocytes, leukocytes

Response was added on 2022-01-11 12:48:48.

Frequency of image registration

Please comment on the frequency of registration of the various image modalities, eg. at specified time points, as needed, in connection with research projects or other.	X-rays done every 2 years in clinical practice.

Response was added on 2022-01-11 12:50:58.

Comorbidities

Which extraarticular manifestations and comorbid conditions are registered? (Tick all that apply)	☒ uveitis<br>☒ psoriasis<br>☒ inflammatory bowel disease (IBD)<br>☒ ischemic heart disease (IHD)<br>☒ cerebrovascular disease (CVD)<br>☒ hypertension<br>☒ diabetes (DM)<br>☒ dyslipidemia<br>☒ osteoporosis<br>☒ chronic kidney insufficiency (CKI)<br>☒ chronic liver disease, eg. hepatitis, cirrhosis<br>☒ other
Please specify	We have something called "other" with blank field where researcher can enter any disease. E.g, surgery, cancer, etc.

Time points for registration

	at inclusion in registry	at start/change of treatment	at follow-up visits	other
uveitis	☒	☒	☒	☐
psoriasis	☒	☒	☒	☐
IBD	☒	☒	☒	☐
IHD	☒	☒	☒	☐
CVD	☒	☒	☒	☐
hypertension	☒	☒	☒	☐
DM	☒	☒	☒	☐
dyslipidemia	☒	☒	☒	☐
osteoporosis	☒	☒	☒	☐
CKI	☒	☒	☒	☐
chronic liver disease	☒	☒	☐	☐
other	☒	☒	☒	☐

Mode of registration - how are comorbid conditions registered?

	patient-reported	by health-staff	linkage from other registry
uveitis	☐	☒	☐
psoriasis	☐	☒	☐
IBD	☐	☒	☐

IHD	☐	☒	☐
CVD	☐	☒	☐
hypertension	☐	☒	☐
DM	☐	☒	☐
dyslipidemia	☐	☒	☐
osteoporosis	☐	☒	☐
CKI	☐	☒	☐
chronic liver disease	☐	☒	☐
other	☐	☒	☐

Do you use ICD-10 codes for registration? ⊗ no

Response was added on 2022-01-11 12:56:12.

Lifestyle

Which lifestyle parameters are registered?
(Tick all that apply)

☒ smoking
☒ alcohol consumption
☒ physical activity

Time points for registration

	at inclusion in registry	at start/change of treatment	at follow-up visits	other
Smoking	☒	☒	☒	☐
Alcohol	☒	☒	☒	☐
Physical activity	☒	☒	☒	☐

Smoking

How is smoking status characterised?
(Tick all that apply)

☒ current
☒ other

Please specify

If someone is "no" smoker (could be never or former, no distinction is made)

Do you register a start date?
(Tick all that apply)

☒ no date is registered

Do you register a stop date for former smokers?

⊗ No

How is average number of smoked cigarettes registered?
(Tick all that apply)

☒ number of daily cigarettes

Alcohol

How is alcohol consumption quantified?
(Tick all that apply)

☒ average number of units/week

Physical activity

How is physical activity defined?
(Tick all that apply)

⊗ not defined

How is physical activity quantified?
(Tick all that apply)

☒ sessions per week
☒ sessions per month

General comments	For physical activities, if a patient has had fysio, sport, individual exercises, cycling, walking, working in garden or has "other" physicial activity. We also report how many times per day or week. Howmany minutes every time.

Response was added on 2022-01-11 12:56:48.

Safety

Can you register adverse events in your registry? (Tick all that apply)	☒ yes, directly into registry
Is it mandatory to register adverse events through your registry?	⊗ no
Which adverse events are registered in your registry? (Tick all that apply)	☒ non-serious adverse events<br>☒ serious adverse events?

Information on adverse events

	date of event	MeddRA	ICD10-code	outcome	other
Non serious AE	☒	☐	☐	☒	☐
Serious AE	☒	☐	☐	☒	☐

General comments	I dont know what MeddRA is.

Participant information

Record ID	9
Name	Florenzo Iannone
Registry	GISEA
E-mail	florenzo.iannone@uniba.it
Deltager	⊗ ja

Response was added on 2022-01-16 08:55:31.

General registry information	
What is the status of your registry?	⊗ running and including patients

Coverage	
Please estimate how many (percentage) of the eligible spondyloarthritis patients in your country, that are registered	15
Please estimate how many (percentage) of the eligible psoriatic arthritis patients in your country, that are registered	10
How did you obtain the coverage estimate above? (Tick all that apply)	☒ other
Please specify	by the the estimated prevalance of the diseases
Which institutions/organisations can include patients in your registry? (Tick all that apply)	☒ departments of rheumatology at hospitals<br>☒ departments of rheumatology at university hospitals
Please give an estimate of how many departments of rheumatology at hospitals (not including university hospitals) that include patients in your registry	4
Please give an estimate of how many departments of rheumatology at university hospitals that include patients in your registry	13
When is the data registered? (Tick all that apply)	☒ at pre-specified registry visits
What is the schedule for pre-specified registry visits?	6 months
Is it possible to register visits outside of the pre-specified visit schedule, eg. if a patient has a flare?	⊗ yes

Ethics

Is approval from a local ethics committee needed for a study on de-identified data (eg. a EuroSpA upload)? (Tick all that apply)	☒ comment
Comment	GISEA has been approved as whole. Further submission to IRB are needed in case of change of the data set already approved
Do patients need to sign informed consent to be included in your registry? (Tick all that apply)	☒ yes
Are any additional local approvals needed for a study on de-identified data (eg. a EuroSpA upload)? (Tick all that apply)	☒ yes
Please specify which additional approvals are needed	In case of new data to collect

Funding

How is the registry funded? (Tick all that apply)	☒ other
Please specify which other sources	Industry sponsorships for events and meetings arranged by GISEA.
Please estimate the percentage of funds coming from other sources	100 ((0-100%))
The percentages add correctly up to a 100%	
What is the basis of participation by a clinic/department/office in the registry? (Tick all that apply)	☒ voluntary
Is the clinic/department/office financially compensated for registration?	⊗ no

Inclusion criteria

What event triggers the inclusion of a patient into the registry? (Tick all that apply)	☒ new treatment<br>☒ other
Please specify	new treatment with bDMARDs or tsDMARDs
Which criteria do you base the inclusion on? (Tick all that apply)	☒ treatment
Is a minimum age required for inclusion?	☒ no

Do patients need to be treated with biological DMARDs (including targeted synthetic DMARDs) to be included in the registry?	⊗ yes
Have the inclusion criteria changed over time?	⊗ no

Response was added on 2022-01-16 09:02:47.

Data management	
What are the options for data entry?<br>(Tick all that apply)	☒ electronic
Are the data fields in your registry interactive, such that invalid or unprobable data is flagged when entered (edit checks)<br>(Tick all that apply)	☒ comment
Comment	We are discussing about flagging the missin of some key data
Please describe any other data validation procedures that you may use	Checking the inconsitency of data
How does your registry retain data management services?<br>(Tick all that apply)	☒ external company
Which is the background of your data manager(s)?<br>(Tick all that apply)	☒ data science/biostatistician/technical
Which are the most commonly used data analysis software/programming languages in your organization?<br>(Tick all that apply)	☒ SPSS
How is the data stored in the registry?<br>(Tick all that apply)	☒ a relational database framework (like SQL)
Which are the main data formats used for raw data extractions?<br>(Tick all that apply)	☒ simple text files, eg. csv, tsv
Where is your data collection platform hosted?<br>(Tick all that apply)	☒ external company
Who maintains your data collection platform?<br>(Tick all that apply)	☒ external company<br>☒ data manager
How frequently is the database updated with the latest information?<br>(Tick all that apply)	☒ monthly
Is linkage to other databases or registries possible?	⊗ yes
Which registries can be linked to?<br>(Tick all that apply)	☒ prescription registry

Response was added on 2022-01-16 09:04:23.

Demography

Please indicate which of the following variables are collected in your registry
(Tick all that apply)

- ☒ age (year of birth)
- ☒ sex
- ☒ ethnicity
- ☒ weight
- ☒ height
- ☒ death

Time points for registration

	at inclusion in registry	at start/change of treatment	at follow-up visits	other
Weight	☒	☐	☐	☐
Height	☒	☐	☐	☐

How is vital status registered
(Tick all that apply)

- ☒ by healthstaff, manually

Diagnosis

How is a diagnosis registered?
(Tick all that apply)

- ☒ through ICD-10 codes
- ☒ classification criteria

Do you register
(Tick all that apply)

- ☒ date for diagnosis

Do you register
(Tick all that apply)

- ☒ date for symptom onset

Time points for registration

	at inclusion in registry	at start/change of treatment	at follow-up visits	other
Day/month/year of diagnosis	☒	☐	☐	☐
Day/month/year of symptom onset	☒	☐	☐	☐

Which classification criteria are registered?
(Tick all that apply)

- ☒ New York
- ☒ CASPAR

	at inclusion in registry	at start/change of treatment	at follow-up visits	other
New York	☒	☐	☐	☐
CASPAR	☒	☐	☐	☐

Do you register individual New York classification items?
(Tick all that apply)

☒ no

Do you register individual CASPAR classification items?
(Tick all that apply)

☒ no

Response was added on 2022-01-16 10:31:20.

Axial spondyloarthritis

Which disease status characteristics can be registered in axSpA patients?
(Tick all that apply)

☒ swollen joint count
☒ tender joint count
☒ enthesitis
☒ dactylitis
☒ physician global
☒ other

Please specify: HAQ, VAS pain, BASDAI

Time points for registration

	at inclusion in registry	at start/change of treatment	at follow-up visits	other
Swollen joint count	☒	☒	☒	☐
Tender joint count	☒	☒	☒	☐
Enthesitis	☒	☒	☒	☐
Dactylitis	☒	☒	☒	☐
Physician global	☒	☒	☒	☐

Swollen joints

How many swollen joint counts can be registered?
(Tick all that apply)

☒ 66

Do you register specific location of swollen joints?
(Tick all that apply)

☒ yes

Tender joints

How many tender joint counts can be registered?
(Tick all that apply)

☒ 68

Do you register specific location of tender joints?
(Tick all that apply)

☒ yes

Enthesitis

Do you register specific location of enthesitis? (Tick all that apply)	☒ no, only as a total count
Please indicate which specific indices that are used, if applicable	LEI

Dactylitis

How is dactylitis assessed? (Tick all that apply)	☒ as a count

Physician Global

Please write the wording of the question regarding physician global	The PhGA was collected on a 0-10 numerical rating scale by asking the physician's "overall assessment of the activity of the axSpA during the last 48 hours," with inactive disease and active disease as anchors (a higher score means higher disease activity)<br>(translate into english if possible)

Coxitis

Do you assess coxitis? If yes, please indicate how it is assessed	no

Response was added on 2022-01-16 10:33:21.

Psoriatic arthritis

Which disease status characteristics can be registered in PsA patients?<br>(Tick all that apply)	☒ swollen joint count<br>☒ tender joint count<br>☒ enthesitis<br>☒ dactylitis<br>☒ skin<br>☒ nails<br>☒ physician Global<br>☒ other
Please specify	VAS pain, HAQ

Time points for registration

	at inclusion in registry	at start/change of treatment	at follow-up visits	other
Swollen joint count	☒	☒	☒	☐
Tender joint count	☒	☒	☒	☐
Enthesitis	☒	☒	☒	☐
Dactylitis	☒	☒	☒	☐
Skin	☒	☒	☒	☐
Nails	☒	☐	☐	☐
Physician Global	☒	☒	☒	☐

Swollen joints

How many swollen joint counts can be registered?<br>(Tick all that apply)	☒ 66
Do you register specific location of swollen joints?<br>(Tick all that apply)	☒ yes

Tender joints

How many tender joint counts can be registered?<br>(Tick all that apply)	☒ 68
Do you register specific location of tender joints?<br>(Tick all that apply)	☒ yes

Entesitis

Do you register specific location of enthesitis? (Tick all that apply)	☒ no, only as a total count

Dactylitis

How is dactylitis assessed? (Tick all that apply)	☒ as a count

Skin

Which instruments are used for registering skin involvment in PsA? (Tick all that apply)	☒ PASI<br>☒ body surface area %

Nails

Which instruments are used for registering nail involvment in PsA? (Tick all that apply)	☒ free text description

Physician global

Please write the wording of the question regarding physician global	The PhGA was collected on a 0-10 numerical rating scale by asking the physician's "overall assessment of the activity of the axSpA during the last 48 hours," with inactive disease and active disease as anchors (a higher score means higher disease activity)<br>(Translate into english if possible)

Coxitis

Do you assess coxitis? If yes, please indicate how it is assessed	no

Response was added on 2022-01-16 10:37:52.

Medication

Which therapies are registered in your registry? (Tick all that apply)	☒ biological dmards (bDMARDs), including targeted synthetic dmards (JAK)<br>☒ conventional synthetic dmards (csDMARDs)<br>☒ glucocorticoids<br>☒ other
Please specify	csDMARDs are recorded only in pts taking bDMARD or tsDMARD

bDMARDs

Which year did bDMARD registration begin?	2010
Is it mandatory to register bDMARD therapy?	⊗ yes

csDMARDs

Which year did csDMARD registration begin?	2010
Is it mandatory to register csDMARD therapy?	⊗ yes

Glucocorticoids

Which year did glucocorticoid registration begin?	2010
Is it mandatory to register glucocorticoid therapy?	⊗ no
Is the mode of administration registered? (Tick all that apply)	☒ oral<br>☒ intramuscular

What information regarding ONGOING medications is registered?

	start date	stop date	temporary start and stop dates	discontinuation reasons	dosage	frequency	administration mode
bDMARDs	☒	☒	☐	☒	☒	☐	☒
csDMARDs	☒	☐	☐	☐	☒	☒	☒
glucocorticoids	☒	☐	☐	☐	☒	☒	☐

Time points for registration (ONGOING medications)

	at inclusion in registry	at start/change of treatment	at follow-up visits	other
bDMARDs	☒	☒	☒	☐
csDMARDs	☒	☒	☒	☐
glucocorticoids	☒	☐	☐	☐

What information regarding PAST medications is registered at inclusion in registry?

	start date	stop date	temporary start and stop dates	discontinuation reasons	dosage	name of drug
bDMARDs	☒	☒	☐	☒	☐	☐

Discontinuation reasons

What are the possible reasons for discontinuation of a bDMARD?
(Tick all that apply)

☒ lack of efficacy
☒ adverse events
☒ remission
☒ pregnancy wish
☒ infection
☒ lost to follow-up
☒ death

Is it possible to register multiple reasons for discontinuation?

⊗ no

Does your registry link to prescription database or other external data sources on prescriptions on a regular basis as input to registry?
(Tick all that apply)

☒ no

Response was added on 2022-01-17 00:08:40.

Patient reported outcomes

Which patient reported outcomes (PROs) are registered in axSpA and/or PsA patients? (Tick all that apply)	☒ BASDAI<br>☒ pain<br>☒ fatigue<br>☒ global disease<br>☒ HAQ
Are any of the PROs registered in either axSpA or PsA only?	⊗ no, all PROs are registered in both diagnoses

Mode of registration

What are the options for registration of PROs? (Tick all that apply)	☒ paper forms<br>☒ through interview with health-staff

Time points for registration

	at inclusion in registry	at start/change of treatment	at follow-up visits	other
BASDAI	☒	☒	☒	☐
Pain	☒	☒	☒	☐
Fatigue	☒	☒	☒	☐
Global disease	☒	☒	☒	☐
HAQ	☒	☒	☒	☐

BASDAI

Are the individidual BASDAI components registered?	⊗ yes

Pain, fatigue and global assessments

Please write the wording of the question relating to pain	"Numerical rating scale ranging from 0 (no pain) to 100 (worst imaginable pain) measuring actual back pain intensity<br>(please translate into english if possible)
Please write the wording of the question relating to fatigue	Numerical rating scale ranging from 0 (no tired at all) to 100 (extremely tired) measuring fatigue intensity during the last 24h"<br>(please translate into english if possible)
Please write the wording of the question relating to global assessment of disease	Considering all the ways your arthritis has affected you, how active do you feel your arthritis is today on a scale ranging from 0 to 100<br>(please translate into english if possible)

HAQ

Are the individual HAQ items registered?	⊗ yes
Which HAQ versions may be used in your registry? (Tick all that apply)	☒ stanford HAQ DI without adjustment for use of aids and devices

Response was added on 2022-01-17 00:09:28.

Laboratory

Which laboratory test can be registered?
(Tick all that apply)

☒ ESR
☒ CRP
☒ hemoglobin
☒ creatinine
☒ HLA-B27
☒ IgM-RF

Time points for registration

	at inclusion in registry	at start/change of treatment	at follow-up visits	other
ESR	☒	☒	☒	☐
CRP	☒	☒	☒	☐
Hemoglobin	☒	☐	☒	☐
Creatinine	☒	☐	☒	☐

How are laboratory test results registered?
(Tick all that apply)

☒ entered by health care staff

Response was added on 2022-01-17 00:13:27.

Frequency of image registration

Please comment on the frequency of registration of the various image modalities, eg. at specified time points, as needed, in connection with research projects or other.	The register allows to upoload imagines but it is not mandatory and therefore very few data are available

Response was added on 2022-01-17 00:14:51.

Comorbidities

Which extraarticular manifestations and comorbid conditions are registered?
(Tick all that apply)

- ☒ uveitis
- ☒ psoriasis
- ☒ inflammatory bowel disease (IBD)
- ☒ ischemic heart disease (IHD)
- ☒ cerebrovascular disease (CVD)
- ☒ hypertension
- ☒ diabetes (DM)
- ☒ dyslipidemia
- ☒ osteoporosis
- ☒ chronic kidney insufficiency (CKI)
- ☒ chronic liver disease, eg. hepatitis, cirrhosis
- ☒ solid cancer
- ☒ hematological cancer
- ☒ depression
- ☒ tuberculosis (TB)
- ☒ fibromyalgia

Time points for registration

	at inclusion in registry	at start/change of treatment	at follow-up visits	other
uveitis	☒	☐	☒	☐
psoriasis	☒	☐	☒	☐
IBD	☒	☐	☒	☐
IHD	☒	☐	☒	☐
CVD	☒	☐	☒	☐
hypertension	☒	☐	☒	☐
DM	☒	☐	☒	☐
dyslipidemia	☒	☐	☒	☐
osteoporosis	☒	☐	☒	☐
CKI	☒	☐	☒	☐
chronic liver disease	☒	☐	☒	☐
solid cancer	☒	☐	☒	☐
hematological cancer	☒	☐	☒	☐
depression	☒	☐	☒	☐
TB	☒	☐	☒	☐
fibromyalgia	☒	☐	☒	☐

Mode of registration - how are comorbid conditions registered?

	patient-reported	by health-staff	linkage from other registry
uveitis	☐	☒	☐
psoriasis	☐	☒	☐
IBD	☐	☒	☐
IHD	☐	☒	☐
CVD	☐	☒	☐
hypertension	☐	☒	☐
DM	☐	☒	☐
dyslipidemia	☐	☒	☐
osteoporosis	☐	☒	☐
CKI	☐	☒	☐
chronic liver disease	☐	☒	☐
solid cancer	☐	☒	☐
hematological cancer	☐	☒	☐
depression	☐	☒	☐
TB	☐	☒	☐
fibromyalgia	☐	☒	☐

Do you use ICD-10 codes for registration? ⊗ yes

Response was added on 2022-01-17 00:15:49.

Lifestyle

Which lifestyle parameters are registered? (Tick all that apply)
☒ smoking
☒ alcohol consumption

Time points for registration

	at inclusion in registry	at start/change of treatment	at follow-up visits	other
Smoking	☒	☐	☐	☐
Alcohol	☒	☐	☐	☐

Smoking

How is smoking status characterised? (Tick all that apply)
☒ current
☒ former
☒ never

Do you register a start date? (Tick all that apply)
☒ no date is registered

Do you register a stop date for former smokers?
⊗ No

How is average number of smoked cigarettes registered? (Tick all that apply)
☒ not registered

Alcohol

How is alcohol consumption quantified? (Tick all that apply)
☒ other

Please specify
it is not quantified

Response was added on 2022-01-17 00:16:17.

Safety

Can you register adverse events in your registry? (Tick all that apply)	☒ yes, directly into registry
Is it mandatory to register adverse events through your registry?	⊗ no
Which adverse events are registered in your registry? (Tick all that apply)	☒ non-serious adverse events<br>☒ serious adverse events?

Information on adverse events

	date of event	MeddRA	ICD10-code	outcome	other
Non serious AE	☒	☐	☒	☐	☐
Serious AE	☒	☐	☒	☐	☐

Participant information

Record ID	10
Name	Anne Gitte Loft
Registry	Danbio
E-mail	Anne.Gitte.Loft@auh.rm.dk
Deltager	⊗ ja

Response was added on 2021-11-23 13:45:44.

General registry information

What is the status of your registry?	⊗ running and including patients

Coverage

Please estimate how many (percentage) of the eligible spondyloarthritis patients in your country, that are registered	95
Please estimate how many (percentage) of the eligible psoriatic arthritis patients in your country, that are registered	85
How did you obtain the coverage estimate above? (Tick all that apply)	☒ other
Please specify	best guess
Which institutions/organisations can include patients in your registry? (Tick all that apply)	☒ private rheumatology practices<br>☒ departments of rheumatology at hospitals<br>☒ departments of rheumatology at university hospitals
Please give an estimate of how many private rheumatology practices that include patients in your registry	approx 25-30
Please give an estimate of how many departments of rheumatology at hospitals (not including university hospitals) that include patients in your registry	approx 22
Please give an estimate of how many departments of rheumatology at university hospitals that include patients in your registry	approx 3
When is the data registered? (Tick all that apply)	☒ at routine visits
Are all routine visits registered in your registry - or only some?	⊗ only some visits

Ethics

Is approval from a local ethics committee needed for a study on de-identified data (eg. a EuroSpA upload)? (Tick all that apply)	☒ no
Do patients need to sign informed consent to be included in your registry? (Tick all that apply)	☒ no
Are any additional local approvals needed for a study on de-identified data (eg. a EuroSpA upload)? (Tick all that apply)	☒ no

Funding

How is the registry funded? (Tick all that apply)	☒ from public sector, eg. state or other<br>☒ industry
Please estimate the percentage of funds coming from public sector, eg. state or other	15<br>((0-100%))
Please estimate the percentage of funds coming from industry, eg. pharmaceutical company	85<br>((0-100%))
The percentages add correctly up to a 100%	
What is the basis of participation by a clinic/department/office in the registry? (Tick all that apply)	☒ mandatory<br>☒ voluntary<br>☒ other
Please specify	mandatory for axspa og psa bdmard, voluntary for psa non bdmard
Is the clinic/department/office financially compensated for registration?	⊗ no

Inclusion criteria

What event triggers the inclusion of a patient into the registry? (Tick all that apply)	☒ new diagnosis
Which criteria do you base the inclusion on? (Tick all that apply)	☒ diagnosis
Is a minimum age required for inclusion?	☒ no
Which diagnoses are included in your registry?<br><br>(Tick all that apply)	☒ ankylosing spondylitis (AS)<br>☒ non-radiographic axial spondyloarthritis (nr-axSpA)<br>☒ psoriatic arthritis (PsA)<br>☒ rheumatoid arthritis<br>☒ other
Which year did inclusion of AS patients begin?	2006

Which year did inclusion of nr-axSpA patients begin?	2009
Which year did inclusion of PsA patients begin?	2006
Which other diagnoses are included?	SLE, juvenil artritis
Have the inclusion criteria changed over time?	⊗ yes
How and when did inclusion criteria change?	2010
General comments	Mandatory to include newly diagnosed patients with SpA since 2010

Response was added on 2021-11-23 13:09:00.

Data management	
What are the options for data entry?<br>(Tick all that apply)	☒ electronic
Are the data fields in your registry interactive, such that invalid or unprobable data is flagged when entered (edit checks)<br>(Tick all that apply)	☒ no<br>☒ comment
Comment	Most fields not interactive/limited, e.g. CRP
Please describe any other data validation procedures that you may use	It is not possible to start a new bdmard before the prior is stopped. Patients can only be registered once nationwide.
How does your registry retain data management services?<br>(Tick all that apply)	☒ external company
Which is the background of your data manager(s)?<br>(Tick all that apply)	☒ data science/biostatistician/technical
Which are the most commonly used data analysis software/programming languages in your organization?<br>(Tick all that apply)	☒ R<br>☒ excel<br>☒ python
How is the data stored in the registry?<br>(Tick all that apply)	☒ a relational database framework (like SQL)
Which are the main data formats used for raw data extractions?<br>(Tick all that apply)	☒ r-derived format<br>☒ SQL-output
Where is your data collection platform hosted?<br>(Tick all that apply)	☒ external company
Who maintains your data collection platform?<br>(Tick all that apply)	☒ external company
How frequently is the database updated with the latest information?<br>(Tick all that apply)	☒ real-time<br>☒ weekly<br>☒ other
Please specify	Real time for patient reported outcomes, daily for death, weekly for group level data
Is linkage to other databases or registries possible?	⊗ yes

Which registries can be linked to?<br>(Tick all that apply)	☒ mortality registry<br>☒ prescription registry<br>☒ comorbidity<br>☒ electronic medical records<br>☒ other
Please specify	soon real time integration with laboratory values eg CRP becomes possible
General comments	LInkages requires approval from authorities and is done on external datasets.<br>Mortality is by linkage daily in registry

Response was added on 2021-11-23 13:10:26.

Demography

Please indicate which of the following variables are collected in your registry
(Tick all that apply)

☒ age (year of birth)
☒ sex
☒ weight
☒ height
☒ death

Time points for registration

	at inclusion in registry	at start/change of treatment	at follow-up visits	other
Weight	☐	☐	☐	☒
Height	☐	☐	☐	☒

Please specify (weight)	Not done routinely
Please specify (height)	Not done routinely

How is vital status registered
(Tick all that apply)

☒ through regular linkage to national register

Diagnosis

How is a diagnosis registered?
(Tick all that apply)

☒ through ICD-10 codes

Do you register
(Tick all that apply)

☒ month of diagnosis
☒ year of diagnosis

Do you register
(Tick all that apply)

☒ month of symptom onset
☒ year of symptom onset

Time points for registration

	at inclusion in registry	at start/change of treatment	at follow-up visits	other
Day/month/year of diagnosis	☒	☒	☒	☐
Day/month/year of symptom onset	☒	☒	☒	☐

Which classification criteria are registered?
(Tick all that apply)

☒ ASAS
☒ New York
☒ CASPAR

Time points for registration

	at inclusion in registry	at start/change of treatment	at follow-up visits	other
ASAS	☐	☐	☐	☒
New York	☐	☐	☐	☒
CASPAR	☐	☐	☐	☒

Please specify (ASAS)	When it is considered relevant
Please specify (New York)	When it is considered relevant
Please specify (CASPAR)	When it is considered relevant
In which patients can you register ASAS?	⊗ both
Do you register individual ASAS classification items? (Tick all that apply)	☒ yes
Do you register individual New York classification items? (Tick all that apply)	☒ yes
Do you register individual CASPAR classification items? (Tick all that apply)	☒ yes
General comments	Concerning registering of classifacation criteria this is not always done - it depends on the clinician

Response was added on 2021-11-23 13:11:10.

Axial spondyloarthritis

Which disease status characteristics can be registered in axSpA patients?
(Tick all that apply)

☒ swollen joint count
☒ tender joint count
☒ enthesitis
☒ physician global
☒ BASMI

Time points for registration

	at inclusion in registry	at start/change of treatment	at follow-up visits	other
Swollen joint count	☒	☒	☒	☐
Tender joint count	☒	☒	☒	☐
Enthesitis	☒	☒	☒	☐
Physician global	☒	☒	☒	☐
BASMI	☒	☒	☒	☐

Swollen joints

How many swollen joint counts can be registered?
(Tick all that apply)

☒ 28
☒ 66

Do you register specific location of swollen joints?
(Tick all that apply)

☒ yes

Tender joints

How many tender joint counts can be registered?
(Tick all that apply)

☒ 28
☒ 68

Do you register specific location of tender joints?
(Tick all that apply)

☒ yes

Enthesitis

Do you register specific location of enthesitis?
(Tick all that apply)

☒ yes

Which locations are registered?<br>(Tick all that apply)	☒ achilles tendon insertion<br>☒ plantar fascia insertion<br>☒ patellar tendon insertion<br>☒ quadriceps insertion<br>☒ supraspinatus insertion<br>☒ medial epicondyle<br>☒ lateral epicondyle<br>☒ greater tochanter
Please indicate which specific indices that are used, if applicable	SPARCC

Physician Global

Please write the wording of the question regarding physician global	Physician evaluation of disease activity (translate into english if possible)

BASMI

Are the individidual BASMI components registered?	⊗ yes
Do you register the individual BASMI measurements (cm, degrees)?	⊗ yes
Which BASMI scale is used?	⊗ 3-point

Coxitis

Do you assess coxitis? If yes, please indicate how it is assessed	No - only pain in the hip joint is registered
General comment	Epsecially enthesitis scores is not always registered - it depends on the physician.<br>BASMI is measured at selected visits.

Response was added on 2021-11-09 08:52:42.

Psoriatic arthritis

Which disease status characteristics can be registered in PsA patients?
(Tick all that apply)

☒ swollen joint count
☒ tender joint count
☒ enthesitis
☒ physician Global
☒ BASMI

Time points for registration

	at inclusion in registry	at start/change of treatment	at follow-up visits	other
Swollen joint count	☒	☒	☒	☐
Tender joint count	☒	☒	☒	☐
Enthesitis	☒	☒	☒	☐
Physician Global	☒	☒	☒	☐
BASMI	☒	☒	☒	☐

Swollen joints

How many swollen joint counts can be registered?
(Tick all that apply)

☒ 28
☒ 66

Do you register specific location of swollen joints?
(Tick all that apply)

☒ yes

Tender joints

How many tender joint counts can be registered?
(Tick all that apply)

☒ 28
☒ 68

Do you register specific location of tender joints?
(Tick all that apply)

☒ yes

Entesitis

Do you register specific location of enthesitis?
(Tick all that apply)

☒ yes

Which locations are registered?<br>(Tick all that apply)	☒ achilles tendon insertion<br>☒ plantar fascia insertion<br>☒ patellar tendon insertion<br>☒ quadriceps insertion<br>☒ supraspinatus insertion<br>☒ medial epicondyle<br>☒ lateral epicondyle<br>☒ greater tochanter
Please indicate which specific indices that are used, if applicable	SPARCC

Physician global

Please write the wording of the question regarding physician global	Physician evaluation of disease activity<br>(Translate into english if possible)

BASMI

Are the individidual BASMI components registered?	⊗ yes
Do you register the individual BASMI measurements (cm, degrees)?	⊗ yes
Which BASMI scale is used?	⊗ 3-point

Coxitis

Do you assess coxitis? If yes, please indicate how it is assessed	No - only tender/not tender hip joint is registered.
General comment	BASMI is normally only registered for patient with axial involvement.<br>In many cases only 28 joints are registered - depending on the physoician

Response was added on 2021-11-23 13:13:12.

Medication

Which therapies are registered in your registry? (Tick all that apply)	☒ biological dmards (bDMARDs), including targeted synthetic dmards (JAK)<br>☒ conventional synthetic dmards (csDMARDs)<br>☒ glucocorticoids<br>☒ NSAIDs

bDMARDs

Which year did bDMARD registration begin?	2009
Is it mandatory to register bDMARD therapy?	⊗ yes

csDMARDs

Which year did csDMARD registration begin?	2009
Is it mandatory to register csDMARD therapy?	⊗ yes

Glucocorticoids

Which year did glucocorticoid registration begin?	2009
Is it mandatory to register glucocorticoid therapy?	⊗ no
Is the mode of administration registered? (Tick all that apply)	☒ oral<br>☒ intramuscular<br>☒ intraarticular
Are specific locations of injected joints registered?	⊗ yes

NSAIDs

Which year did NSAID registration begin?	2012
Is it mandatory to register NSAID therapy?	⊗ yes

What information regarding ONGOING medications is registered?

	start date	stop date	temporary start and stop dates	discontinuation reasons	dosage	frequency	administration mode
bDMARDs	☒	☒	☒	☒	☒	☒	☒
csDMARDs	☒	☒	☒	☒	☒	☒	☒
glucocorticoids	☒	☒	☒	☒	☒	☒	☒
NSAIDs	☒	☒	☒	☒	☒	☒	☒

Time points for registration (ONGOING medications)

	at inclusion in registry	at start/change of treatment	at follow-up visits	other
bDMARDs	☐	☒	☒	☐
csDMARDs	☐	☒	☒	☐
glucocorticoids	☐	☒	☒	☐
NSAIDs	☐	☒	☒	☐

What information regarding PAST medications is registered at inclusion in registry?

	start date	stop date	temporary start and stop dates	discontinuation reasons	dosage	name of drug
bDMARDs	☐	☐	☐	☐	☐	☒
csDMARDs	☐	☐	☐	☐	☐	☒
glucocorticoids	☐	☐	☐	☐	☐	☒
NSAIDs	☐	☐	☐	☐	☐	☒

Discontinuation reasons

What are the possible reasons for discontinuation of a bDMARD?
(Tick all that apply)

☒ lack of efficacy
☒ adverse events
☒ remission
☒ pregnancy wish
☒ infection
☒ surgery
☒ cancer
☒ lost to follow-up
☒ death
☒ other

Please specify: project participation and no information given

Is it possible to register multiple reasons for discontinuation?

⊗ yes

How is it decided which is the primary reason for discontinuation?

That is it not possible to register

Does your registry link to prescription database or other external data sources on prescriptions on a regular basis as input to registry?
(Tick all that apply)

☒ no

General comments	I general NSAIDs is registered for axSpA and axPsA. csDMARDs and prednisolone might not always be registered.

Response was added on 2021-11-23 13:44:37.

Patient reported outcomes

Which patient reported outcomes (PROs) are registered in axSpA and/or PsA patients? (Tick all that apply)	☒ BASDAI<br>☒ BASFI<br>☒ pain<br>☒ fatigue<br>☒ global disease<br>☒ HAQ<br>☒ EQ-5D<br>☒ patient acceptable symptom state (PASS)<br>☒ external anchor question
Are any of the PROs registered in either axSpA or PsA only?	⊗ no, all PROs are registered in both diagnoses

Mode of registration

What are the options for registration of PROs? (Tick all that apply)	☒ paper forms<br>☒ on screen in waiting room<br>☒ through website/app

Time points for registration

	at inclusion in registry	at start/change of treatment	at follow-up visits	other
BASDAI	☒	☒	☒	☐
BASFI	☒	☒	☒	☐
Pain	☒	☒	☒	☐
Fatigue	☒	☒	☒	☐
Global disease	☒	☒	☒	☐
HAQ	☒	☒	☒	☐
EQ-5D	☐	☐	☐	☒
PASS	☒	☒	☒	☐
Anchor	☒	☒	☒	☐

Please specify (EQ-5D)	annually

BASDAI

Are the individidual BASDAI components registered?	⊗ yes

BASFI

Are the individidual BASFI components registered?	⊗ yes

Pain, fatigue and global assessments

Please write the wording of the question relating to pain	Hvor mange gigtsmerter har du for tiden/ How much pain due to arthritis do you have at the moment<br>(please translate into english if possible)
Please write the wording of the question relating to fatigue	Hvor træt er du for tiden/ how tired are you at the moment<br>(please translate into english if possible)
Please write the wording of the question relating to global assessment of disease	Hvor meget påvirker gigten som helhed Deres tilværelse for tiden/How does the arthrits affect your overall life at the moment<br>(please translate into english if possible)

HAQ

Are the individual HAQ items registered?	⊗ yes
Which HAQ versions may be used in your registry? (Tick all that apply)	☒ MD-HAQ

EQ-5D

Are the individual EQ-5D items registered?	⊗ yes
Which EQ-5D version is used?	5L

Patient acceptable symptom state (PASS)

Please write the wording of the question relating to PASS	Tænk på alle de måder, som din gigt har påvirket dig de seneste 48 timer. Hvis du i de kommende måneder fortsatte med at have det, som du har haft det de seneste 48 timer, ville det så være acceptabelt for dig?<br>(please translate into english if possible)

External anchor question

Please write the wording of the external anchor question	Since your last visit, has your arthritis become (please translate into english if possible)
How many response categories are used?	7
General comments	IT is intended to get all patient to answer the questions regarding PROs. however, some patients forget to do it before the visit, promise to do it afterwards - but forget about it

Response was added on 2021-11-09 09:13:19.

Laboratory

Which laboratory test can be registered? (Tick all that apply)	☒ CRP<br>☒ Hba1c<br>☒ cholesterol<br>☒ HLA-B27<br>☒ IgM-RF<br>☒ stored samples available for later analyses (biobank)

	at inclusion in registry	at start/change of treatment	at follow-up visits	other
CRP	☒	☒	☒	☐
Hba1c	☐	☐	☐	☒
Cholesterol	☐	☐	☐	☒
Stored samples available for later analyses (biobank)	☐	☐	☐	☒

Please specify (Hba1c)	at yearly visits
Please specify (cholesterol)	at yearly visits
Please specify (biobank)	typically at project visits

Cholesterol

Which specific cholesterol types are registered? (Tick all that apply)	☒ total cholesterol<br>☒ LDL<br>☒ HDL<br>☒ triglycerid

Biobank

Which sample types are collected? (Tick all that apply)	☒ whole-blood<br>☒ serum<br>☒ plasma
How are laboratory test results registered? (Tick all that apply)	☒ entered by health care staff

Response was added on 2021-11-09 09:20:58.

Imaging

Which imaging modalities can be registered? (Tick all that apply)	☒ Magnetic Resonance Imaging (MRI)<br>☒ X-ray<br>☒ DXA<br>☒ Ultrasound (US)

Information on each image

	date of examination	full image report	image file	scoring system	+/- progression	other
What information on each MRI exam is registered?	☒	☐	☐	☒	☐	☒
What information on each x-ray exam is registered?	☒	☐	☐	☒	☒	☒
What information on each DXA exam is registered?	☒	☐	☐	☒	☒	☐
What information on each US exam is registered?	☒	☐	☐	☒	☐	☒

Please specify (MRI)	SI-led: 4 graderinger, Columna: +/- inflammation.
Please specify (x-ray)	SI:ledl: +/- NY kriterier opfyldt, columna: +/-inflammation, hænder/fødder: erosion/ikke erosion
Please specify (US)	68 joints, b-mode 0-3 and doppler 0-3

MRI

Which anatomical regions can be registered? (Tick all that apply)	☒ spine MRI<br>☒ sacroiliac joint MRI

X-ray

Which anatomical regions can be registered? (Tick all that apply)	☒ spine radiographs<br>☒ sacroiliac joint radiographs<br>☒ hands and feet

DEXA

Which anatomical regions can be registered? (Tick all that apply)	☒ lumbar spine<br>☒ femoral neck

Ultrasound

Please indicate which anatomical regions that can be registered	jaw, AC- and SC- shoulder, elbow, wrist, MCP, PIP, DIP hip, knee, foot, MTP and PIP

Frequency of image registration

Please comment on the frequency of registration of the various image modalities, eg. at specified time points, as needed, in connection with research projects or other.	MR at diagnosis, as needed and related to projects<br>X-ray SI-joints and spine at diagnosis, as needed and related to projects<br>X-ray hands and feet as needed
General comments	It is mandatory to registerer data of examination, precises results are not required

Response was added on 2021-11-23 13:16:23.

Comorbidities

Which extraarticular manifestations and comorbid conditions are registered?<br>(Tick all that apply)	☒ uveitis<br>☒ psoriasis<br>☒ inflammatory bowel disease (IBD)<br>☒ ischemic heart disease (IHD)<br>☒ cerebrovascular disease (CVD)<br>☒ hypertension<br>☒ diabetes (DM)<br>☒ dyslipidemia<br>☒ osteoporosis<br>☒ chronic liver disease, eg. hepatitis, cirrhosis<br>☒ depression<br>☒ tuberculosis (TB)<br>☒ other
Please specify	bronchitis, astma, metabolic disease, gastric ulcer, DS, depression

Time points for registration

	at inclusion in registry	at start/change of treatment	at follow-up visits	other
uveitis	☒	☐	☒	☐
psoriasis	☒	☒	☐	☐
IBD	☒	☒	☐	☐
IHD	☐	☐	☐	☒
CVD	☐	☐	☐	☒
hypertension	☐	☐	☐	☒
DM	☐	☐	☐	☒
dyslipidemia	☐	☐	☐	☒
osteoporosis	☐	☐	☐	☒
chronic liver disease	☐	☐	☐	☒
depression	☐	☐	☐	☒
TB	☐	☒	☐	☐
other	☐	☐	☐	☒

Please specify (IHD)	at annual status, given by the patient
Please specify (CVD)	at annual status, given by the patient
Please specify (hypertension)	at annual status, given by the patient
Please specify (DM)	at annual status, given by the patient

Please specify (dyslipidemia)	at annual status, given by the patient
Please specify (osteoporosis)	at annual status, given by the patient. DEXA scans are entered manually including BMD
Please specify (chronic liver disease)	at annual status, given by the patient
Please specify (depression)	EQ-5D
Please specify (other)	at annual status, given by the patient

Mode of registration - how are comorbid conditions registered?

	patient-reported	by health-staff	linkage from other registry
uveitis	☒	☒	☐
psoriasis	☒	☒	☐
IBD	☒	☒	☐
IHD	☒	☐	☐
CVD	☒	☐	☐
hypertension	☒	☐	☐
DM	☒	☐	☐
dyslipidemia	☒	☒	☐
osteoporosis	☒	☒	☐
chronic liver disease	☒	☐	☐
depression	☒	☐	☐
TB	☒	☒	☐
other	☒	☐	☐

Do you use ICD-10 codes for registration?	⊗ no
General comments	It is not mandatory to make annual registrations. Therefore, data are not collected for all patients

Response was added on 2021-11-23 13:17:44.

Lifestyle

Which lifestyle parameters are registered?
(Tick all that apply)

☒ smoking
☒ alcohol consumption
☒ physical activity

Time points for registration

	at inclusion in registry	at start/change of treatment	at follow-up visits	other
Smoking	☐	☐	☐	☒
Alcohol	☐	☐	☐	☒
Physical activity	☐	☐	☐	☒

Please specify (smoking): Annaul registration, by the patient

Please specify (alcohol): Annaul registration, by the patient

Please specify (physical activity): Annaul registration, by the patient

Smoking

How is smoking status characterised?
(Tick all that apply)

☒ current
☒ former
☒ never

Do you register a start date?
(Tick all that apply)

☒ for current smokers
☒ for former smokers

Do you register a stop date for former smokers?

⊗ yes

How is average number of smoked cigarettes registered?
(Tick all that apply)

☒ number of daily cigarettes
☒ other

Please specify: number of pipe stops per day and number og cigars and cerutter per day

Alcohol

How is alcohol consumption quantified?<br>(Tick all that apply)	☒ average number of units/week<br>☒ other
Please specify	Også muligt at vælge binær variabel: over/under sundhedsstyrelsens anbefaling.

Physical activity

How is physical activity defined?<br>(Tick all that apply)	⊗ not defined
How is physical activity quantified?<br>(Tick all that apply)	☒ sessions per week<br>☒ sessions per month

Response was added on 2021-11-09 09:29:28.

Safety

Can you register adverse events in your registry? (Tick all that apply)	☒ yes, directly into registry
Is it mandatory to register adverse events through your registry?	⊗ no
Which adverse events are registered in your registry? (Tick all that apply)	☒ non-serious adverse events<br>☒ serious adverse events?

Information on adverse events

	date of event	MeddRA	ICD10-code	outcome	other
Non serious AE	☒	☐	☐	☒	☒
Serious AE	☒	☐	☐	☒	☐

Please specify (non serious AE)	In new treatments recently marketed

Participant information

Record ID	11
Name	Gary Macfarlane
Registry	BSRBR-AS
E-mail	epidemiology@abdn.ac.uk
Deltager	⊗ ja

Response was added on 2021-11-23 15:12:39.

General registry information

What is the status of your registry?	⊗ closed

Coverage

Please estimate how many (percentage) of the eligible spondyloarthritis patients in your country, that are registered	0
How did you obtain the coverage estimate above? (Tick all that apply)	☒ other
Please specify	Our estimate is 0.5% This is based on the paper Hamilton et al (2015)
Which institutions/organisations can include patients in your registry? (Tick all that apply)	☒ departments of rheumatology at hospitals<br>☒ departments of rheumatology at university hospitals
Please give an estimate of how many departments of rheumatology at hospitals (not including university hospitals) that include patients in your registry	40
Please give an estimate of how many departments of rheumatology at university hospitals that include patients in your registry	40
When is the data registered? (Tick all that apply)	☒ at routine visits
Are all routine visits registered in your registry - or only some?	⊗ all visits

Ethics

Is approval from a local ethics committee needed for a study on de-identified data (eg. a EuroSpA upload)? (Tick all that apply)	☒ no
Do patients need to sign informed consent to be included in your registry? (Tick all that apply)	☒ yes
Are any additional local approvals needed for a study on de-identified data (eg. a EuroSpA upload)? (Tick all that apply)	☒ yes

Please specify which additional approvals are needed	We are required to seek approval from the British Society for Rheumatology

Funding

How is the registry funded?<br>(Tick all that apply)	☒ from research grants<br>☒ industry
Please estimate the percentage of funds coming from research grants	2<br>((0-100%))
Please estimate the percentage of funds coming from industry, eg. pharmaceutical company	98<br>((0-100%))
The percentages add correctly up to a 100%	
What is the basis of participation by a clinic/department/office in the registry?<br>(Tick all that apply)	☒ voluntary
Is the clinic/department/office financially compensated for registration?	⊗ other
Please specify	They are indirectly compensated: recruitment to research studies (such as registry) provides research nurse support

Inclusion criteria

What event triggers the inclusion of a patient into the registry?<br>(Tick all that apply)	☒ new treatment<br>☒ other
Please specify	The treatment group are starting biologic therapy for the first time, the comparison group are those biologic naive and not starting such therapy.
Which criteria do you base the inclusion on?<br>(Tick all that apply)	☒ age<br>☒ diagnosis<br>☒ treatment
Is a minimum age required for inclusion?	☒ other age limit
Please specify the age limit applied	16 years or above
Which diagnoses are included in your registry?<br><br>(Tick all that apply)	☒ ankylosing spondylitis (AS)<br>☒ non-radiographic axial spondyloarthritis (nr-axSpA)
Which year did inclusion of AS patients begin?	2012
Which year did inclusion of nr-axSpA patients begin?	2014

Do patients need to be treated with biological DMARDs (including targeted synthetic DMARDs) to be included in the registry?	⊗ no
Have the inclusion criteria changed over time?	⊗ yes
How and when did inclusion criteria change?	In 2014 patients who met clinical ASAS criteria only became eligible

Response was added on 2021-11-23 15:12:43.

Data management

What are the options for data entry?<br>(Tick all that apply)	☒ paper based<br>☒ electronic
Are the data fields in your registry interactive, such that invalid or unprobable data is flagged when entered (edit checks)<br>(Tick all that apply)	☒ comment
Comment	Some fields (e.g. dates)
Please describe any other data validation procedures that you may use	We did have double data entry for a small proportion of questionnaires
How does your registry retain data management services?<br>(Tick all that apply)	☒ a researcher/administrative personal does data management beside other duties
Which is the background of your data manager(s)?<br>(Tick all that apply)	☒ other
Please specify	Administrator
Which are the most commonly used data analysis software/programming languages in your organization?<br>(Tick all that apply)	☒ R<br>☒ stata
How is the data stored in the registry?<br>(Tick all that apply)	☒ a relational database framework (like SQL)<br>☒ other
Please specify	Stata
Which are the main data formats used for raw data extractions?<br>(Tick all that apply)	☒ other
Other specify	Stata
Where is your data collection platform hosted?<br>(Tick all that apply)	☒ other
Please specify	University
Who maintains your data collection platform?<br>(Tick all that apply)	☒ data manager

How frequently is the database updated with the latest information?<br>(Tick all that apply)	☒ other
Please specify	Data collection is complete
Is linkage to other databases or registries possible?	⊗ yes
Which registries can be linked to?<br>(Tick all that apply)	☒ mortality registry<br>☒ prescription registry<br>☒ comorbidity
General comments	Mortality linkage is possible; Prescription and other morbidity registers are only available in Scotland. We are not linked at present and this would be a considerable amount of work and considerable cost.

Response was added on 2021-11-23 15:12:46.

Demography

Please indicate which of the following variables are collected in your registry (Tick all that apply)	☒ age (year of birth)<br>☒ sex<br>☒ weight<br>☒ height

Time points for registration

	at inclusion in registry	at start/change of treatment	at follow-up visits	other
Weight	☒	☐	☒	☐
Height	☒	☐	☒	☐

Diagnosis

How is a diagnosis registered? (Tick all that apply)	☒ classification criteria
Do you register (Tick all that apply)	☒ other
Please specify	Year first seen by a rheumatologist
Do you register (Tick all that apply)	☒ year of symptom onset

Time points for registration

	at inclusion in registry	at start/change of treatment	at follow-up visits	other
Day/month/year of diagnosis	☒	☐	☐	☐
Day/month/year of symptom onset	☒	☐	☐	☐

Which classification criteria are registered? (Tick all that apply)	☒ ASAS<br>☒ New York

Time points for registration

	at inclusion in registry	at start/change of treatment	at follow-up visits	other
ASAS	☒	☐	☐	☐
New York	☒	☐	☐	☐

In which patients can you register ASAS?	⊗ AxSpA
Do you register individual ASAS classification items? (Tick all that apply)	☒ yes
Do you register individual New York classification items? (Tick all that apply)	☒ yes
General comments	Weight and Height are also available when commencing an eligible biologic drug

Response was added on 2021-11-23 15:12:53.

Axial spondyloarthritis

Which disease status characteristics can be registered in axSpA patients? (Tick all that apply)	☒ swollen joint count<br>☒ tender joint count<br>☒ enthesitis<br>☒ BASMI

Time points for registration

	at inclusion in registry	at start/change of treatment	at follow-up visits	other
Swollen joint count	☒	☐	☒	☐
Tender joint count	☒	☐	☒	☐
Enthesitis	☒	☐	☒	☐
BASMI	☒	☐	☒	☐

Swollen joints

How many swollen joint counts can be registered? (Tick all that apply)	☒ other
Please specify	40
Do you register specific location of swollen joints? (Tick all that apply)	☒ yes

Tender joints

How many tender joint counts can be registered? (Tick all that apply)	☒ 44
Do you register specific location of tender joints? (Tick all that apply)	☒ yes

Enthesitis

Do you register specific location of enthesitis? (Tick all that apply)	☒ no
Please indicate which specific indices that are used, if applicable	N/A

BASMI

Are the individidual BASMI components registered?	⊗ yes
Do you register the individual BASMI measurements (cm, degrees)?	⊗ no
Which BASMI scale is used?	⊗ 11-point

Coxitis

Do you assess coxitis? If yes, please indicate how it is assessed	No

Response was added on 2021-11-23 15:13:06.

Medication

Which therapies are registered in your registry? (Tick all that apply)	☒ biological dmards (bDMARDs), including targeted synthetic dmards (JAK)<br>☒ NSAIDs

bDMARDs

Which year did bDMARD registration begin?	2012
Is it mandatory to register bDMARD therapy?	⊗ no

NSAIDs

Which year did NSAID registration begin?	2012
Is it mandatory to register NSAID therapy?	⊗ no

What information regarding ONGOING medications is registered?

	start date	stop date	temporary start and stop dates	discontinuation reasons	dosage	frequency	administration mode
bDMARDs	☒	☒	☒	☒	☒	☒	☐

Time points for registration (ONGOING medications)

	at inclusion in registry	at start/change of treatment	at follow-up visits	other
bDMARDs	☒	☒	☒	☐

	start date	stop date	temporary start and stop dates	discontinuation reasons	dosage	name of drug
NSAIDs	☐	☐	☐	☐	☐	☒

Discontinuation reasons

What are the possible reasons for discontinuation of a bDMARD? (Tick all that apply)	☒ lack of efficacy<br>☒ adverse events<br>☒ remission<br>☒ pregnancy wish<br>☒ death<br>☒ other
Please specify	(1) Participant decision, (2) Financial (NHS level)

Is it possible to register multiple reasons for discontinuation?	⊗ no
Does your registry link to prescription database or other external data sources on prescriptions on a regular basis as input to registry? (Tick all that apply)	☒ no

Response was added on 2021-11-23 15:13:09.

Patient reported outcomes

Which patient reported outcomes (PROs) are registered in axSpA and/or PsA patients? (Tick all that apply)	☒ BASDAI<br>☒ BASFI<br>☒ pain<br>☒ fatigue<br>☒ global disease<br>☒ EQ-5D<br>☒ other
Please specify	ACR 2011 fibromyalgia criteria
Are any of the PROs registered in either axSpA or PsA only?	⊗ yes
Which PROs are registered uniquely in either axSpA or PsA?	All above = axSpA only. (Our PsA data is not yet in EuroSpA.)

Mode of registration

What are the options for registration of PROs? (Tick all that apply)	☒ paper forms

Time points for registration

	at inclusion in registry	at start/change of treatment	at follow-up visits	other
BASDAI	☒	☒	☒	☐
BASFI	☒	☒	☒	☐
Pain	☒	☒	☒	☐
Fatigue	☒	☒	☒	☐
Global disease	☒	☒	☒	☐
EQ-5D	☒	☒	☒	☐

BASDAI

Are the individidual BASDAI components registered?	⊗ yes

BASFI

Are the individidual BASFI components registered?	⊗ yes

Pain, fatigue and global assessments

Please write the wording of the question relating to pain	Various, including pain question in SF12, EQ5D, 100mm VAS<br>(please translate into english if possible)
Please write the wording of the question relating to fatigue	Chalder Fatigue Scale<br>(please translate into english if possible)
Please write the wording of the question relating to global assessment of disease	BAS-G<br>(please translate into english if possible)

EQ-5D

Are the individual EQ-5D items registered?	⊗ yes
Which EQ-5D version is used?	5L
Which algorithm is used?	Crosswalk values available from: https://euroqol.org/eq-5d-instruments/eq-5d-5l-about/valuation-standard-value-sets/crosswalk-index-value-calculator/ Based on: van Hout et al. Interim scoring for the EQ-5D-5L: Mapping the EQ-5D-5L to EQ-5D-3L value sets. Value in Health 2012; 15(5): 708-15.

Response was added on 2021-11-23 15:13:12.

Laboratory

Which laboratory test can be registered?
(Tick all that apply)

☒ ESR
☒ CRP
☒ HLA-B27

Time points for registration

	at inclusion in registry	at start/change of treatment	at follow-up visits	other
ESR	☒	☒	☒	☐
CRP	☒	☒	☒	☐

How are laboratory test results registered?
(Tick all that apply)

☒ entered by health care staff

General comments

Protocol required CRP or ESR.
For some participants there are both.

Response was added on 2021-11-23 15:13:15.

General comments	We have no images.<br>We have data at registration re:<br>~ Bilateral sacroiliitis (Gr2) or unilateral (Gr3) on x-ray, and/or<br>~ Active inflammation on MRI suggestive of SpA; but we have no images as part of the registry.

Response was added on 2021-11-23 15:13:18.

Comorbidities

Which extraarticular manifestations and comorbid conditions are registered? (Tick all that apply)	☒ uveitis<br>☒ psoriasis<br>☒ inflammatory bowel disease (IBD)<br>☒ ischemic heart disease (IHD)<br>☒ cerebrovascular disease (CVD)<br>☒ hypertension<br>☒ diabetes (DM)<br>☒ osteoporosis<br>☒ chronic kidney insufficiency (CKI)<br>☒ chronic liver disease, eg. hepatitis, cirrhosis<br>☒ solid cancer<br>☒ hematological cancer<br>☒ depression<br>☒ tuberculosis (TB)<br>☒ fibromyalgia<br>☒ other
Please specify	Hip/Spine surgery

Time points for registration

	at inclusion in registry	at start/change of treatment	at follow-up visits	other
uveitis	☒	☒	☒	☐
psoriasis	☒	☒	☒	☐
IBD	☒	☒	☒	☐
IHD	☒	☒	☒	☐
CVD	☒	☒	☒	☐
hypertension	☒	☒	☒	☐
DM	☒	☒	☒	☐
osteoporosis	☒	☒	☒	☐
CKI	☒	☒	☒	☐
chronic liver disease	☒	☒	☒	☐
solid cancer	☒	☒	☒	☐
hematological cancer	☒	☒	☒	☐
depression	☒	☒	☒	☐
TB	☒	☒	☒	☐
fibromyalgia	☒	☒	☒	☐
other	☒	☒	☒	☐

Mode of registration - how are comorbid conditions registered?

	patient-reported	by health-staff	linkage from other registry
uveitis	☐	☒	☐
psoriasis	☐	☒	☐
IBD	☐	☒	☐
IHD	☐	☒	☐
CVD	☐	☒	☐
hypertension	☐	☒	☐
DM	☐	☒	☐
osteoporosis	☐	☒	☐
CKI	☐	☒	☐
chronic liver disease	☐	☒	☐
solid cancer	☐	☒	☐
hematological cancer	☐	☒	☐
depression	☐	☒	☐
TB	☐	☒	☐
fibromyalgia	☒	☒	☐
other	☐	☒	☐

Do you use ICD-10 codes for registration?	⊗ no
General comments	Comorbidities recorded at registration. If new event during following, may constitute an AE/SAE and addition data collection would take place.

Response was added on 2021-11-23 15:13:22.

Lifestyle

Which lifestyle parameters are registered? (Tick all that apply)	☒ smoking<br>☒ alcohol consumption<br>☒ other
Please specify	Work, including WPAI:SHP

Time points for registration

	at inclusion in registry	at start/change of treatment	at follow-up visits	other
Smoking	☒	☐	☒	☐
Alcohol	☒	☐	☒	☐

Smoking

How is smoking status characterised? (Tick all that apply)	☒ current<br>☒ former<br>☒ never
Do you register a start date? (Tick all that apply)	☒ no date is registered
Do you register a stop date for former smokers?	⊗ No
How is average number of smoked cigarettes registered? (Tick all that apply)	☒ number of daily cigarettes

Alcohol

How is alcohol consumption quantified? (Tick all that apply)	☒ average number of units/week
General comments	Daily cigarettes only recorded in those with "daily or almost daily" tobacco use.<br>Alcohol consumption only in those with "weekly, daily or almost daily" alcohol use.<br>Data collection is PROM (questionnaire) at registration and at questionnaire follow-up.

Response was added on 2021-11-23 15:13:25.

Safety

Can you register adverse events in your registry? (Tick all that apply)	☒ yes, directly into registry
Is it mandatory to register adverse events through your registry?	⊗ no
Which adverse events are registered in your registry? (Tick all that apply)	☒ non-serious adverse events<br>☒ serious adverse events?<br>☒ other
Other	Events of special interest (incident comorbidity (see previous list) and/or pregnancy)

Information on adverse events

	date of event	MeddRA	ICD10-code	outcome	other
Non serious AE	☒	☒	☐	☒	☐
Serious AE	☒	☒	☐	☒	☐

Participant information

Record ID	12
Name	Daniela Di Giuseppe
Registry	ARTIS
E-mail	daniela.digiuseppe@ki.se
Deltager	⊗ ja

Response was added on 2022-01-24 21:32:14.

General registry information

What is the status of your registry?	⊗ running and including patients

Coverage

Please estimate how many (percentage) of the eligible spondyloarthritis patients in your country, that are registered	82
Please estimate how many (percentage) of the eligible psoriatic arthritis patients in your country, that are registered	76
How did you obtain the coverage estimate above? (Tick all that apply)	☒ by comparison with other national registries of therapy, diagnoses etc.
Which institutions/organisations can include patients in your registry? (Tick all that apply)	☒ private rheumatology practices<br>☒ departments of rheumatology at hospitals<br>☒ departments of rheumatology at university hospitals
Please give an estimate of how many private rheumatology practices that include patients in your registry	10
Please give an estimate of how many departments of rheumatology at hospitals (not including university hospitals) that include patients in your registry	31
Please give an estimate of how many departments of rheumatology at university hospitals that include patients in your registry	7
When is the data registered? (Tick all that apply)	☒ at routine visits
Are all routine visits registered in your registry - or only some?	⊗ only some visits

Ethics

Is approval from a local ethics committee needed for a study on de-identified data (eg. a EuroSpA upload)?<br>(Tick all that apply)	☒ yes
Do patients need to sign informed consent to be included in your registry?<br>(Tick all that apply)	☒ no
Are any additional local approvals needed for a study on de-identified data (eg. a EuroSpA upload)?<br>(Tick all that apply)	☒ no

Funding

How is the registry funded?<br>(Tick all that apply)	☒ from public sector, eg. state or other<br>☒ industry
Please estimate the percentage of funds coming from public sector, eg. state or other	50<br>((0-100%))
Please estimate the percentage of funds coming from industry, eg. pharmaceutical company	50<br>((0-100%))
The percentages add correctly up to a 100%	
What is the basis of participation by a clinic/department/office in the registry?<br>(Tick all that apply)	☒ voluntary
Is the clinic/department/office financially compensated for registration?	⊗ no

Inclusion criteria

What event triggers the inclusion of a patient into the registry?<br>(Tick all that apply)	☒ new diagnosis<br>☒ new treatment
Which criteria do you base the inclusion on?<br>(Tick all that apply)	☒ diagnosis<br>☒ treatment<br>☒ disease activity
Is a minimum age required for inclusion?	☒ yes, 18 years or above
Which diagnoses are included in your registry?<br><br>(Tick all that apply)	☒ ankylosing spondylitis (AS)<br>☒ non-radiographic axial spondyloarthritis (nr-axSpA)<br>☒ psoriatic arthritis (PsA)<br>☒ rheumatoid arthritis<br>☒ other
Which year did inclusion of AS patients begin?	1999
Which year did inclusion of nr-axSpA patients begin?	1999

Which year did inclusion of PsA patients begin?	1999
Which other diagnoses are included?	All treated in rheumatology units
Do patients need to be treated with biological DMARDs (including targeted synthetic DMARDs) to be included in the registry?	⊗ no
Please specify any disease activity inclusion criteria applied for patients with AS or nr-axSpA, eg. ASDAS, BASDAI, ASAS or other.	none
Please specify any disease activity inclusion criteria applied for patients with PsA, eg. DAS28 ,DAPSA28 or other.	none
Have the inclusion criteria changed over time?	⊗ no
General comments	The coverage is only regarding b/tsDMARD patients, and it is based on a preliminary analyses done as part of a research project. The final numbers will probably be higher.

Response was added on 2022-01-24 21:32:19.

Data management

What are the options for data entry?<br>(Tick all that apply)	☒ electronic
Are the data fields in your registry interactive, such that invalid or unprobable data is flagged when entered (edit checks)<br>(Tick all that apply)	☒ yes
Since when (year) has the data fields been interactive?	2003
How does your registry retain data management services?<br>(Tick all that apply)	☒ person employed as data manager
Which is the background of your data manager(s)?<br>(Tick all that apply)	☒ data science/biostatistician/technical
Which are the most commonly used data analysis software/programming languages in your organization?<br>(Tick all that apply)	☒ R<br>☒ SAS<br>☒ stata<br>☒ excel<br>☒ SPSS
How is the data stored in the registry?<br>(Tick all that apply)	☒ a relational database framework (like SQL)
Which are the main data formats used for raw data extractions?<br>(Tick all that apply)	☒ excel<br>☒ SAS-derived format
Where is your data collection platform hosted?<br>(Tick all that apply)	☒ external company
Who maintains your data collection platform?<br>(Tick all that apply)	☒ external company
How frequently is the database updated with the latest information?<br>(Tick all that apply)	☒ real-time
Is linkage to other databases or registries possible?	⊗ yes
Which registries can be linked to?<br>(Tick all that apply)	☒ mortality registry<br>☒ prescription registry<br>☒ comorbidity

General comments	The linkage is possible, but only for researchers that have ethical permission. We at the register do not link our data to external registers, with the exception of the mortality register to update information on death date

Response was added on 2022-01-24 21:32:23.

Demography

Please indicate which of the following variables are collected in your registry<br>(Tick all that apply)	☒ age (year of birth)<br>☒ sex<br>☒ weight<br>☒ height<br>☒ death

Time points for registration

	at inclusion in registry	at start/change of treatment	at follow-up visits	other
Weight	☐	☐	☐	☒
Height	☐	☐	☐	☒

Please specify (weight)	rarely registered
Please specify (height)	rarely registered
How is vital status registered<br>(Tick all that apply)	☒ through regular linkage to national register

Diagnosis

How is a diagnosis registered?<br>(Tick all that apply)	☒ through ICD-10 codes<br>☒ classification criteria
Do you register<br>(Tick all that apply)	☒ date for diagnosis
Do you register<br>(Tick all that apply)	☒ date for symptom onset

Time points for registration

	at inclusion in registry	at start/change of treatment	at follow-up visits	other
Day/month/year of diagnosis	☒	☐	☐	☐
Day/month/year of symptom onset	☒	☐	☐	☐

Which classification criteria are registered?<br>(Tick all that apply)	☒ ASAS<br>☒ New York<br>☒ CASPAR

Time points for registration

	at inclusion in registry	at start/change of treatment	at follow-up visits	other
ASAS	☒	☐	☐	☒
New York	☒	☐	☐	☒
CASPAR	☒	☐	☐	☒

Please specify (ASAS)	complemented or registered later if changed or incomplete
Please specify (New York)	complemented or registered later if changed or incomplete
Please specify (CASPAR)	complemented or registered later if changed or incomplete
In which patients can you register ASAS?	⊗ both
Do you register individual ASAS classification items? (Tick all that apply)	☒ yes
Do you register individual New York classification items? (Tick all that apply)	☒ yes
Do you register individual CASPAR classification items? (Tick all that apply)	☒ yes
General comments	The use of classification criteria is not mandatory

Response was added on 2022-01-24 21:32:26.

Axial spondyloarthritis

Which disease status characteristics can be registered in axSpA patients? (Tick all that apply)	☒ swollen joint count<br>☒ tender joint count<br>☒ enthesitis<br>☒ dactylitis<br>☒ physician global<br>☒ BASMI<br>☒ other
Please specify	Basdai, basfi

Time points for registration

	at inclusion in registry	at start/change of treatment	at follow-up visits	other
Swollen joint count	☒	☒	☒	☐
Tender joint count	☒	☒	☒	☐
Enthesitis	☒	☒	☒	☐
Dactylitis	☒	☒	☒	☐
Physician global	☒	☒	☒	☐
BASMI	☒	☒	☒	☐

Swollen joints

How many swollen joint counts can be registered? (Tick all that apply)	☒ 28<br>☒ 66
Do you register specific location of swollen joints? (Tick all that apply)	☒ no, just joint counts

Tender joints

How many tender joint counts can be registered? (Tick all that apply)	☒ 28<br>☒ 68
Do you register specific location of tender joints? (Tick all that apply)	☒ no, just joint counts

Enthesitis

Do you register specific location of enthesitis? (Tick all that apply)	☒ no, only as a total count
Please indicate which specific indices that are used, if applicable	Leeds

Dactylitis

How is dactylitis assessed? (Tick all that apply)	☒ as a count

Physician Global

Please write the wording of the question regarding physician global	Disease activity: Integers (mm) on a scale between 0 and 100, where 0 means the absence of disease activity and 100 corresponds to the highest possible disease activity. (translate into english if possible)

BASMI

Are the individidual BASMI components registered?	⊗ yes
Do you register the individual BASMI measurements (cm, degrees)?	⊗ yes
Which BASMI scale is used?	⊗ 11-point

Coxitis

Do you assess coxitis? If yes, please indicate how it is assessed	no

Response was added on 2022-01-24 21:32:30.

Psoriatic arthritis

Which disease status characteristics can be registered in PsA patients?
(Tick all that apply)

- ☒ swollen joint count
- ☒ tender joint count
- ☒ enthesitis
- ☒ dactylitis
- ☒ skin
- ☒ nails
- ☒ physician Global
- ☒ BASMI
- ☒ other

Please specify: dapsa, mda. BASMI only for pts with axial disease

Time points for registration

	at inclusion in registry	at start/change of treatment	at follow-up visits	other
Swollen joint count	☒	☒	☒	☐
Tender joint count	☒	☒	☒	☐
Enthesitis	☒	☒	☒	☐
Dactylitis	☒	☒	☒	☐
Skin	☒	☒	☒	☐
Nails	☒	☒	☒	☐
Physician Global	☒	☒	☒	☐
BASMI	☒	☒	☒	☐

Swollen joints

How many swollen joint counts can be registered?
(Tick all that apply)

- ☒ 28
- ☒ 66

Do you register specific location of swollen joints?
(Tick all that apply)

- ☒ no, just joint counts

Tender joints

How many tender joint counts can be registered?
(Tick all that apply)

- ☒ 28
- ☒ 68

Do you register specific location of tender joints?
(Tick all that apply)

- ☒ no, just joint counts

Entesitis

Do you register specific location of enthesitis? (Tick all that apply)	☒ no, only as a total count
Please indicate which specific indices that are used, if applicable	Leeds

Dactylitis

How is dactylitis assessed? (Tick all that apply)	☒ as a count

Skin

Which instruments are used for registering skin involvment in PsA? (Tick all that apply)	☒ body surface area %

Nails

Which instruments are used for registering nail involvment in PsA? (Tick all that apply)	☒ other
Other	yes/no variable

Physician global

Please write the wording of the question regarding physician global	Disease activity: Integers (mm) on a scale between 0 and 100, where 0 means the absence of disease activity and 100 corresponds to the highest possible disease activity.<br>(Translate into english if possible)

BASMI

Are the individidual BASMI components registered?	⊗ yes
Do you register the individual BASMI measurements (cm, degrees)?	⊗ yes
Which BASMI scale is used?	⊗ 11-point

Coxitis

Do you assess coxitis? If yes, please indicate how it is assessed	no

Response was added on 2022-01-24 21:32:34.

Medication

Which therapies are registered in your registry? (Tick all that apply)	☒ biological dmards (bDMARDs), including targeted synthetic dmards (JAK)<br>☒ conventional synthetic dmards (csDMARDs)<br>☒ glucocorticoids<br>☒ NSAIDs

bDMARDs

Which year did bDMARD registration begin?	1995
Is it mandatory to register bDMARD therapy?	⊗ no

csDMARDs

Which year did csDMARD registration begin?	1995
Is it mandatory to register csDMARD therapy?	⊗ no

Glucocorticoids

Which year did glucocorticoid registration begin?	1995
Is it mandatory to register glucocorticoid therapy?	⊗ no
Is the mode of administration registered? (Tick all that apply)	☒ oral

NSAIDs

Which year did NSAID registration begin?	1995
Is it mandatory to register NSAID therapy?	⊗ no

What information regarding ONGOING medications is registered?

	start date	stop date	temporary start and stop dates	discontinuation reasons	dosage	frequency	administration mode
bDMARDs	☒	☒	☐	☒	☒	☒	☒
csDMARDs	☒	☒	☐	☒	☒	☒	☒

glucocorticoids	☒	☒	☐	☒	☒	☒	☒
NSAIDs	☒	☒	☐	☒	☒	☒	☒

Time points for registration (ONGOING medications)

	at inclusion in registry	at start/change of treatment	at follow-up visits	other
bDMARDs	☒	☒	☒	☐
csDMARDs	☒	☒	☒	☐
glucocorticoids	☒	☒	☒	☐
NSAIDs	☒	☒	☒	☐

What information regarding PAST medications is registered at inclusion in registry?

	start date	stop date	temporary start and stop dates	discontinuation reasons	dosage	name of drug
bDMARDs	☐	☐	☐	☐	☐	☒
csDMARDs	☐	☐	☐	☐	☐	☒
glucocorticoids	☐	☐	☐	☐	☐	☒
NSAIDs	☐	☐	☐	☐	☐	☒

Discontinuation reasons

What are the possible reasons for discontinuation of a bDMARD?
(Tick all that apply)

☒ lack of efficacy
☒ adverse events
☒ remission
☒ pregnancy wish
☒ surgery
☒ death
☒ other

Is it possible to register multiple reasons for discontinuation?

⊗ no

Does your registry link to prescription database or other external data sources on prescriptions on a regular basis as input to registry?
(Tick all that apply)

☒ no

General comments

the information on dosage and administration mode are not mandatory, and not always reported

Response was added on 2022-01-24 21:32:38.

Patient reported outcomes

Which patient reported outcomes (PROs) are registered in axSpA and/or PsA patients? (Tick all that apply)	☒ BASDAI<br>☒ BASFI<br>☒ pain<br>☒ fatigue<br>☒ global disease<br>☒ HAQ<br>☒ EQ-5D
Are any of the PROs registered in either axSpA or PsA only?	⊗ yes
Which PROs are registered uniquely in either axSpA or PsA?	basdai, basfi

Mode of registration

What are the options for registration of PROs? (Tick all that apply)	☒ paper forms<br>☒ through interview with health-staff<br>☒ on screen in waiting room<br>☒ through website/app

Time points for registration

	at inclusion in registry	at start/change of treatment	at follow-up visits	other
BASDAI	☒	☒	☒	☐
BASFI	☒	☒	☒	☐
Pain	☒	☒	☒	☐
Fatigue	☒	☒	☒	☐
Global disease	☒	☒	☒	☐
HAQ	☒	☒	☒	☐
EQ-5D	☒	☒	☒	☐

BASDAI

Are the individidual BASDAI components registered?	⊗ yes

BASFI

Are the individidual BASFI components registered?	⊗ yes

Pain, fatigue and global assessments

Please write the wording of the question relating to pain	How much pain have you had in the last week due to your rheumatic disease?<br>(please translate into english if possible)
Please write the wording of the question relating to fatigue	How tired have you been in the last week due to your rheumatic disease?<br>(please translate into english if possible)
Please write the wording of the question relating to global assessment of disease	How have you felt in the last week, in general, given your rheumatic disease?<br>(please translate into english if possible)

HAQ

Are the individual HAQ items registered?	⊗ yes
Which HAQ versions may be used in your registry? (Tick all that apply)	☒ stanford HAQ DI with adjustment for use of aids and devices

EQ-5D

Are the individual EQ-5D items registered?	⊗ yes
Which EQ-5D version is used?	We use EQ5D 3L
Which algorithm is used?	The algorithm used is the standard algorithm for UK

Response was added on 2022-01-24 21:32:41.

Laboratory

Which laboratory test can be registered?
(Tick all that apply)

☒ ESR
☒ CRP

Time points for registration

	at inclusion in registry	at start/change of treatment	at follow-up visits	other
ESR	☒	☒	☒	☐
CRP	☒	☒	☒	☐

How are laboratory test results registered?
(Tick all that apply)

☒ entered by health care staff

Response was added on 2022-01-24 21:32:46.

Comorbidities

Which extraarticular manifestations and comorbid conditions are registered?
(Tick all that apply)

☒ uveitis
☒ psoriasis
☒ inflammatory bowel disease (IBD)

Time points for registration

	at inclusion in registry	at start/change of treatment	at follow-up visits	other
uveitis	☐	☐	☒	☐
psoriasis	☐	☐	☒	☐
IBD	☐	☐	☒	☐

Mode of registration - how are comorbid conditions registered?

	patient-reported	by health-staff	linkage from other registry
uveitis	☐	☒	☐
psoriasis	☐	☒	☐
IBD	☐	☒	☐

Do you use ICD-10 codes for registration? ⊗ no

Response was added on 2022-01-24 21:32:49.

Lifestyle

Which lifestyle parameters are registered? (Tick all that apply)	☒ smoking<br>☒ physical activity

Time points for registration

	at inclusion in registry	at start/change of treatment	at follow-up visits	other
Smoking	☒	☒	☒	☐
Physical activity	☒	☒	☒	☐

Smoking

How is smoking status characterised? (Tick all that apply)	☒ current<br>☒ former<br>☒ never
Do you register a start date? (Tick all that apply)	☒ no date is registered
Do you register a stop date for former smokers?	⊗ No
How is average number of smoked cigarettes registered? (Tick all that apply)	☒ not registered

Physical activity

How is physical activity defined? (Tick all that apply)	⊗ other definition
Please specify	How much time do you spend a regular week on physical exercise that causes you to become short of breath, such as running, gymnastics or ball sports?
How is physical activity quantified? (Tick all that apply)	☒ other
Please specify	Time per week

General comments	We are using the national indicator questions decided by Socialstyrelsen (National Board of Health and Welfare). These include 3 questions; the one above + How much time do you spend a regular week doing everyday exercise, such as walking, cycling or gardening? Add up all the time (at least 10 minutes at a time). + How much do you sit during a normal day if you exclude sleep?. All answers as categorical time intervals

Response was added on 2022-01-24 21:32:52.

Safety

Can you register adverse events in your registry? (Tick all that apply)	☒ yes, directly into registry
Is it mandatory to register adverse events through your registry?	⊗ no
Which adverse events are registered in your registry? (Tick all that apply)	☒ non-serious adverse events<br>☒ serious adverse events?

Information on adverse events

	date of event	MeddRA	ICD10-code	outcome	other
Non serious AE	☒	☒	☐	☒	☐
Serious AE	☒	☒	☐	☒	☐

Participant information

Record ID	13
Name	Jacub Zàvada
Registry	ATTRA
E-mail	zavada@revma.cz
Deltager	⊗ ja

Response was added on 2021-12-11 15:59:08.

General registry information

What is the status of your registry?	⊗ running and including patients

Coverage

Please estimate how many (percentage) of the eligible spondyloarthritis patients in your country, that are registered	95
Please estimate how many (percentage) of the eligible psoriatic arthritis patients in your country, that are registered	95
How did you obtain the coverage estimate above?<br>(Tick all that apply)	☒ by prior study of coverage
Which institutions/organisations can include patients in your registry?<br>(Tick all that apply)	☒ private rheumatology practices<br>☒ departments of rheumatology at hospitals<br>☒ departments of rheumatology at university hospitals
Please give an estimate of how many private rheumatology practices that include patients in your registry	45
Please give an estimate of how many departments of rheumatology at hospitals (not including university hospitals) that include patients in your registry	9
Please give an estimate of how many departments of rheumatology at university hospitals that include patients in your registry	6
When is the data registered?<br>(Tick all that apply)	☒ at pre-specified registry visits
What is the schedule for pre-specified registry visits?	0, 3, 6 months, then every 6 months. After 3 years, anually
Is it possible to register visits outside of the pre-specified visit schedule, eg. if a patient has a flare?	⊗ yes

Ethics

Is approval from a local ethics committee needed for a study on de-identified data (eg. a EuroSpA upload)? (Tick all that apply)	☒ no
Do patients need to sign informed consent to be included in your registry? (Tick all that apply)	☒ yes
Are any additional local approvals needed for a study on de-identified data (eg. a EuroSpA upload)? (Tick all that apply)	☒ no

Funding

How is the registry funded? (Tick all that apply)	☒ from research grants<br>☒ industry
Please estimate the percentage of funds coming from research grants	5<br>((0-100%))
Please estimate the percentage of funds coming from industry, eg. pharmaceutical company	95<br>((0-100%))
The percentages add correctly up to a 100%	
What is the basis of participation by a clinic/department/office in the registry? (Tick all that apply)	☒ mandatory
Is the clinic/department/office financially compensated for registration?	⊗ yes

Inclusion criteria

What event triggers the inclusion of a patient into the registry? (Tick all that apply)	☒ new treatment
Which criteria do you base the inclusion on? (Tick all that apply)	☒ diagnosis<br>☒ treatment
Is a minimum age required for inclusion?	☒ no
Which diagnoses are included in your registry?<br><br>(Tick all that apply)	☒ ankylosing spondylitis (AS)<br>☒ non-radiographic axial spondyloarthritis (nr-axSpA)<br>☒ psoriatic arthritis (PsA)<br>☒ rheumatoid arthritis<br>☒ other
Which year did inclusion of AS patients begin?	2002
Which year did inclusion of nr-axSpA patients begin?	2012

Which year did inclusion of PsA patients begin?	2002
Which other diagnoses are included?	JIA
Do patients need to be treated with biological DMARDs (including targeted synthetic DMARDs) to be included in the registry?	⊗ yes
Have the inclusion criteria changed over time?	⊗ no

Response was added on 2021-12-11 16:10:34.

Data management	
What are the options for data entry?<br>(Tick all that apply)	☒ paper based<br>☒ electronic
Are the data fields in your registry interactive, such that invalid or unprobable data is flagged when entered (edit checks)<br>(Tick all that apply)	☒ no
Please describe any other data validation procedures that you may use	users are being reminded to fill missing core data
How does your registry retain data management services?<br>(Tick all that apply)	☒ person employed as data manager
Which is the background of your data manager(s)?<br>(Tick all that apply)	☒ data science/biostatistician/technical
Which are the most commonly used data analysis software/programming languages in your organization?<br>(Tick all that apply)	☒ excel<br>☒ SPSS
How is the data stored in the registry?<br>(Tick all that apply)	☒ a relational database framework (like SQL)
Which are the main data formats used for raw data extractions?<br>(Tick all that apply)	☒ excel<br>☒ SQL-output<br>☒ custom/composite format
Where is your data collection platform hosted?<br>(Tick all that apply)	☒ other
Please specify	Institute of Biostatistics and Analyses
Who maintains your data collection platform?<br>(Tick all that apply)	☒ data manager
How frequently is the database updated with the latest information?<br>(Tick all that apply)	☒ real-time
Is linkage to other databases or registries possible?	⊗ no

Response was added on 2021-12-11 16:22:14.

Demography

Please indicate which of the following variables are collected in your registry
(Tick all that apply)

- ☒ age (year of birth)
- ☒ sex
- ☒ weight
- ☒ height
- ☒ death

Time points for registration

	at inclusion in registry	at start/change of treatment	at follow-up visits	other
Weight	☒	☐	☐	☐
Height	☒	☐	☐	☐

How is vital status registered
(Tick all that apply)

- ☒ by healthstaff, manually

Diagnosis

How is a diagnosis registered?
(Tick all that apply)

- ☒ other diagnostic categories

Specify which diagnostic categories

physician diagnosis

Do you register
(Tick all that apply)

- ☒ month of diagnosis
- ☒ year of diagnosis

Do you register
(Tick all that apply)

- ☒ month of symptom onset
- ☒ year of symptom onset

Time points for registration

	at inclusion in registry	at start/change of treatment	at follow-up visits	other
Day/month/year of diagnosis	☒	☒	☐	☐
Day/month/year of symptom onset	☒	☒	☐	☐

Which classification criteria are registered?
(Tick all that apply)

- ☒ none

General comments

ATTRA collects most but not all items of classification criteria (ASAS/NY). In Psa we ask if pt fulfills some set of criteria (Moll Wright or CASPAR)

Response was added on 2021-12-11 16:29:15.

Axial spondyloarthritis

Which disease status characteristics can be registered in axSpA patients?
(Tick all that apply)

- ☒ swollen joint count
- ☒ enthesitis
- ☒ dactylitis
- ☒ physician global

Time points for registration

	at inclusion in registry	at start/change of treatment	at follow-up visits	other
Swollen joint count	☒	☒	☒	☐
Enthesitis	☒	☒	☒	☐
Dactylitis	☒	☒	☒	☐
Physician global	☒	☒	☒	☐

Swollen joints

How many swollen joint counts can be registered?
(Tick all that apply)

- ☒ 44

Do you register specific location of swollen joints?
(Tick all that apply)

- ☒ yes

Enthesitis

Do you register specific location of enthesitis?
(Tick all that apply)

- ☒ yes

Which locations are registered?
(Tick all that apply)

- ☒ achilles tendon insertion
- ☒ plantar fascia insertion
- ☒ 1st costochondral joint
- ☒ 7th costochondral joint
- ☒ posterior superior iliac spine
- ☒ anterior superior iliac spine
- ☒ iliac crests
- ☒ 5th lumbar spinous process

Please indicate which specific indices that are used, if applicable

MASES

Dactylitis

How is dactylitis assessed? (Tick all that apply)	☒ as a count

Physician Global

Please write the wording of the question regarding physician global	Global activity assessed by physician (VAS 0-100) (translate into english if possible)

Coxitis

Do you assess coxitis? If yes, please indicate how it is assessed	No

Response was added on 2021-12-11 16:37:05.

Psoriatic arthritis

Which disease status characteristics can be registered in PsA patients? (Tick all that apply)	☒ swollen joint count<br>☒ tender joint count<br>☒ enthesitis<br>☒ dactylitis<br>☒ skin<br>☒ nails<br>☒ physician Global

Time points for registration

	at inclusion in registry	at start/change of treatment	at follow-up visits	other
Swollen joint count	☒	☒	☒	☐
Tender joint count	☒	☒	☒	☐
Enthesitis	☒	☒	☒	☐
Dactylitis	☒	☒	☒	☐
Skin	☒	☒	☒	☐
Nails	☒	☒	☒	☐
Physician Global	☒	☒	☒	☐

Swollen joints

How many swollen joint counts can be registered? (Tick all that apply)	☒ 66
Do you register specific location of swollen joints? (Tick all that apply)	☒ yes

Tender joints

How many tender joint counts can be registered? (Tick all that apply)	☒ 68
Do you register specific location of tender joints? (Tick all that apply)	☒ yes

Entesitis

Do you register specific location of enthesitis? (Tick all that apply)	☒ yes
Which locations are registered? (Tick all that apply)	☒ achilles tendon insertion<br>☒ plantar fascia insertion

Dactylitis

How is dactylitis assessed? (Tick all that apply)	☒ as a count

Skin

Which instruments are used for registering skin involvment in PsA? (Tick all that apply)	☒ other
Other	psoriasis severity global assessment (0-5)

Nails

Which instruments are used for registering nail involvment in PsA? (Tick all that apply)	☒ other
Other	semiquantitive (none/mild/moderate/severe)

Physician global

Please write the wording of the question regarding physician global	"physian global activity assessement (VAS 0-100)<br>(Translate into english if possible)

Coxitis

Do you assess coxitis? If yes, please indicate how it is assessed	via 66/68 joint count

Response was added on 2021-12-11 16:45:47.

Medication

Which therapies are registered in your registry? (Tick all that apply)	☒ biological dmards (bDMARDs), including targeted synthetic dmards (JAK)<br>☒ conventional synthetic dmards (csDMARDs)<br>☒ glucocorticoids<br>☒ NSAIDs<br>☒ medication for comorbidity

bDMARDs

Which year did bDMARD registration begin?	2002
Is it mandatory to register bDMARD therapy?	⊗ yes

csDMARDs

Which year did csDMARD registration begin?	2012
Is it mandatory to register csDMARD therapy?	⊗ yes

Glucocorticoids

Which year did glucocorticoid registration begin?	2012
Is it mandatory to register glucocorticoid therapy?	⊗ yes
Is the mode of administration registered? (Tick all that apply)	☒ oral

NSAIDs

Which year did NSAID registration begin?	2012
Is it mandatory to register NSAID therapy?	⊗ no

Medicaton for comorbidities

What types of medical therapy for comorbidities are registered?	selected groups (e.g.medication for hypertnesion, diabetes, depression..) and free string

What information regarding ONGOING medications is registered?

	start date	stop date	temporary start and stop dates	discontinuation reasons	dosage	frequency	administration mode
bDMARDs	☒	☒	☐	☒	☒	☒	☐
csDMARDs	☒	☒	☐	☐	☒	☒	☐
glucocorticoids	☒	☒	☐	☐	☒	☒	☐

Time points for registration (ONGOING medications)

	at inclusion in registry	at start/change of treatment	at follow-up visits	other
bDMARDs	☒	☒	☒	☐
csDMARDs	☒	☐	☒	☐
glucocorticoids	☒	☐	☒	☐
NSAIDs	☒	☐	☒	☐
medication for comorbidity	☒	☐	☒	☐

What information regarding PAST medications is registered at inclusion in registry?

	start date	stop date	temporary start and stop dates	discontinuation reasons	dosage	name of drug
bDMARDs	☐	☐	☐	☐	☐	☒
csDMARDs	☐	☐	☐	☐	☐	☒
glucocorticoids	☐	☐	☐	☐	☐	☒
NSAIDs	☐	☐	☐	☐	☐	☒

Discontinuation reasons

What are the possible reasons for discontinuation of a bDMARD?
(Tick all that apply)

☒ lack of efficacy
☒ adverse events
☒ remission
☒ lost to follow-up
☒ death
☒ other

Is it possible to register multiple reasons for discontinuation?

⊗ no

Does your registry link to prescription database or other external data sources on prescriptions on a regular basis as input to registry?
(Tick all that apply)

☒ no

Response was added on 2021-12-11 16:54:18.

Patient reported outcomes

Which patient reported outcomes (PROs) are registered in axSpA and/or PsA patients? (Tick all that apply)	☒ BASDAI<br>☒ BASFI<br>☒ pain<br>☒ fatigue<br>☒ global disease<br>☒ HAQ<br>☒ EQ-5D<br>☒ other
Please specify	WPAI, SF36
Are any of the PROs registered in either axSpA or PsA only?	⨂ yes
Which PROs are registered uniquely in either axSpA or PsA?	BASDAI and BASFI only for AxSpA

Mode of registration

What are the options for registration of PROs? (Tick all that apply)	☒ paper forms<br>☒ on screen in waiting room<br>☒ through website/app

Time points for registration

	at inclusion in registry	at start/change of treatment	at follow-up visits	other
BASDAI	☒	☒	☒	☐
BASFI	☒	☒	☒	☐
Pain	☒	☒	☒	☐
Fatigue	☒	☒	☒	☐
Global disease	☒	☒	☒	☐
HAQ	☒	☒	☒	☐
EQ-5D	☒	☒	☒	☐

BASDAI

Are the individidual BASDAI components registered?	⊗ yes

BASFI

Are the individidual BASFI components registered?	⊗ yes

Pain, fatigue and global assessments

Please write the wording of the question relating to pain	How much pain has your illness caused you DURING THE PAST WEEK? Please indicate how unpleasant your pain was:<br>(please translate into english if possible)
Please write the wording of the question relating to fatigue	How much of a problem has unsual fatigue been for you DURING THE PAST WEEK?<br>(please translate into english if possible)
Please write the wording of the question relating to global assessment of disease	Please indicate below how you feel when you consider all the ways in which your illness now affects you<br>(please translate into english if possible)

HAQ

Are the individual HAQ items registered?	⊗ yes
Which HAQ versions may be used in your registry? (Tick all that apply)	☒ stanford HAQ DI with adjustment for use of aids and devices<br>☒ M-HAQ<br>☒ MD-HAQ

EQ-5D

Are the individual EQ-5D items registered?	⊗ yes
Which EQ-5D version is used?	3L

Response was added on 2021-12-11 16:55:22.

Laboratory

Which laboratory test can be registered?
(Tick all that apply)

☒ ESR
☒ CRP
☒ HLA-B27
☒ IgM-RF

Time points for registration

	at inclusion in registry	at start/change of treatment	at follow-up visits	other
ESR	☒	☒	☒	☐
CRP	☒	☒	☒	☐

How are laboratory test results registered?
(Tick all that apply)

☒ entered by health care staff

Response was added on 2021-12-11 16:57:23.

Imaging

Which imaging modalities can be registered? (Tick all that apply)	☒ Magnetic Resonance Imaging (MRI)<br>☒ X-ray

MRI

Which anatomical regions can be registered? (Tick all that apply)	☒ sacroiliac joint MRI

X-ray

Which anatomical regions can be registered? (Tick all that apply)	☒ spine radiographs<br>☒ sacroiliac joint radiographs

Frequency of image registration

Please comment on the frequency of registration of the various image modalities, eg. at specified time points, as needed, in connection with research projects or other.	at baseline we ask if MRI or XRAY was performed, and if erosive/osteoproductive changes have been present

Response was added on 2021-12-11 16:59:15.

Comorbidities

Which extraarticular manifestations and comorbid conditions are registered?
(Tick all that apply)

- ☒ uveitis
- ☒ psoriasis
- ☒ inflammatory bowel disease (IBD)
- ☒ ischemic heart disease (IHD)
- ☒ cerebrovascular disease (CVD)
- ☒ hypertension
- ☒ diabetes (DM)
- ☒ dyslipidemia
- ☒ osteoporosis
- ☒ chronic kidney insufficiency (CKI)
- ☒ chronic liver disease, eg. hepatitis, cirrhosis
- ☒ solid cancer
- ☒ hematological cancer
- ☒ depression
- ☒ tuberculosis (TB)

Time points for registration

	at inclusion in registry	at start/change of treatment	at follow-up visits	other
uveitis	☒	☐	☐	☐
psoriasis	☒	☐	☒	☐
IBD	☒	☐	☐	☐
IHD	☒	☐	☐	☐
CVD	☒	☐	☐	☐
hypertension	☒	☐	☐	☐
DM	☒	☐	☐	☐
dyslipidemia	☒	☐	☐	☐
osteoporosis	☒	☐	☐	☐
CKI	☒	☐	☐	☐
chronic liver disease	☒	☐	☐	☐
solid cancer	☒	☐	☐	☐
hematological cancer	☒	☐	☐	☐
depression	☒	☐	☐	☐
TB	☒	☐	☐	☐

Mode of registration - how are comorbid conditions registered?

	patient-reported	by health-staff	linkage from other registry
uveitis	☐	☒	☐
psoriasis	☐	☒	☐
IBD	☐	☒	☐
IHD	☐	☒	☐
CVD	☐	☒	☐
hypertension	☐	☒	☐
DM	☐	☒	☐
dyslipidemia	☐	☒	☐
osteoporosis	☐	☒	☐
CKI	☐	☒	☐
chronic liver disease	☐	☒	☐
solid cancer	☐	☒	☐
hematological cancer	☐	☒	☐
depression	☐	☒	☐
TB	☐	☒	☐

Do you use ICD-10 codes for registration? ⊗ no

Response was added on 2021-12-11 17:00:47.

Lifestyle

Which lifestyle parameters are registered? (Tick all that apply)	☒ smoking

Time points for registration

	at inclusion in registry	at start/change of treatment	at follow-up visits	other
Smoking	☒	☐	☒	☐

Smoking

How is smoking status characterised? (Tick all that apply)	☒ current<br>☒ former<br>☒ never
Do you register a start date? (Tick all that apply)	☒ no date is registered
Do you register a stop date for former smokers?	⊗ No
How is average number of smoked cigarettes registered? (Tick all that apply)	☒ number of daily cigarettes

Response was added on 2021-12-11 17:01:26.

Safety

Can you register adverse events in your registry? (Tick all that apply)	☒ yes, directly into registry
Is it mandatory to register adverse events through your registry?	⊗ no
Which adverse events are registered in your registry? (Tick all that apply)	☒ serious adverse events?

	date of event	MeddRA	ICD10-code	outcome	other
Serious AE	☒	☒	☐	☐	☐

Participant information

Record ID	14
Name	Gerður María Gröndal
Registry	ICEBIO
E-mail	gerdurgr@landspitali.is
Deltager	⊗ ja

Response was added on 2021-12-17 16:03:11.

General registry information

What is the status of your registry?	⊗ running and including patients

Coverage

Please estimate how many (percentage) of the eligible spondyloarthritis patients in your country, that are registered	95
Please estimate how many (percentage) of the eligible psoriatic arthritis patients in your country, that are registered	95
How did you obtain the coverage estimate above? (Tick all that apply)	☒ by comparison with other national registries of therapy, diagnoses etc.<br>☒ by prior study of coverage<br>☒ other
Please specify	The number is 95% of those on biologics
Which institutions/organisations can include patients in your registry? (Tick all that apply)	☒ private rheumatology practices<br>☒ departments of rheumatology at hospitals<br>☒ departments of rheumatology at university hospitals
Please give an estimate of how many private rheumatology practices that include patients in your registry	7
Please give an estimate of how many departments of rheumatology at hospitals (not including university hospitals) that include patients in your registry	0
Please give an estimate of how many departments of rheumatology at university hospitals that include patients in your registry	1
When is the data registered? (Tick all that apply)	☒ at routine visits
Are all routine visits registered in your registry - or only some?	⊗ only some visits

Ethics

Is approval from a local ethics committee needed for a study on de-identified data (eg. a EuroSpA upload)? (Tick all that apply)	☒ yes
Do patients need to sign informed consent to be included in your registry? (Tick all that apply)	☒ no<br>☒ comment
Comment	registry is part of their e-medical chart
Are any additional local approvals needed for a study on de-identified data (eg. a EuroSpA upload)? (Tick all that apply)	☒ yes<br>☒ comment
Please specify which additional approvals are needed	Data protection authorities, Research committee of the Landspitali
Comment	see above

Funding

How is the registry funded? (Tick all that apply)	☒ from public sector, eg. state or other
Please estimate the percentage of funds coming from public sector, eg. state or other	100<br>((0-100%))
The percentages add correctly up to a 100%	
What is the basis of participation by a clinic/department/office in the registry? (Tick all that apply)	☒ mandatory
Is the clinic/department/office financially compensated for registration?	⊗ no

Inclusion criteria

What event triggers the inclusion of a patient into the registry? (Tick all that apply)	☒ new treatment<br>☒ other
Please specify	when starting biological treatment
Which criteria do you base the inclusion on? (Tick all that apply)	☒ diagnosis<br>☒ treatment<br>☒ disease activity<br>☒ other
Is a minimum age required for inclusion?	☒ yes, 18 years or above

Which diagnoses are included in your registry?<br><br>(Tick all that apply)	☒ ankylosing spondylitis (AS)<br>☒ non-radiographic axial spondyloarthritis (nr-axSpA)<br>☒ psoriatic arthritis (PsA)<br>☒ rheumatoid arthritis<br>☒ other
Which year did inclusion of AS patients begin?	2008
Which year did inclusion of nr-axSpA patients begin?	unknown
Which year did inclusion of PsA patients begin?	2008
Which other diagnoses are included?	RA, Arthritis uns, reactive arthritis
Do patients need to be treated with biological DMARDs (including targeted synthetic DMARDs) to be included in the registry?	⊗ other
Which other treatment criteria are applied?	most are on biologics but not all
Please specify any disease activity inclusion criteria applied for patients with AS or nr-axSpA, eg. ASDAS, BASDAI, ASAS or other.	mostly for application of biological treatmen
Please specify any disease activity inclusion criteria applied for patients with PsA, eg. DAS28 ,DAPSA28 or other.	DAS28CRP, BASDAI and/or ASDAS
Please specify which additional inclusion criteria	see above, in need of biologics
Have the inclusion criteria changed over time?	⊗ no

Response was added on 2021-12-17 16:05:58.

Data management

What are the options for data entry? (Tick all that apply)	☒ paper based<br>☒ electronic
Are the data fields in your registry interactive, such that invalid or unprobable data is flagged when entered (edit checks) (Tick all that apply)	☒ no
How does your registry retain data management services? (Tick all that apply)	☒ external company
Which is the background of your data manager(s)? (Tick all that apply)	☒ data science/biostatistician/technical
Which are the most commonly used data analysis software/programming languages in your organization? (Tick all that apply)	☒ R
How is the data stored in the registry? (Tick all that apply)	☒ a relational database framework (like SQL)
Which are the main data formats used for raw data extractions? (Tick all that apply)	☒ excel<br>☒ r-derived format
Where is your data collection platform hosted? (Tick all that apply)	☒ external company
Who maintains your data collection platform? (Tick all that apply)	☒ external company
How frequently is the database updated with the latest information? (Tick all that apply)	☒ real-time
Is linkage to other databases or registries possible?	⊗ yes
Which registries can be linked to? (Tick all that apply)	☒ mortality registry<br>☒ prescription registry<br>☒ comorbidity<br>☒ electronic medical records

Response was added on 2021-12-17 16:09:02.

Demography

Please indicate which of the following variables are collected in your registry
(Tick all that apply)

☒ age (year of birth)
☒ sex
☒ weight
☒ height

Time points for registration

	at inclusion in registry	at start/change of treatment	at follow-up visits	other
Weight	☒	☐	☐	☐
Height	☒	☐	☐	☐

Diagnosis

How is a diagnosis registered?
(Tick all that apply)

☒ through ICD-10 codes

Do you register
(Tick all that apply)

☒ month of diagnosis
☒ year of diagnosis

Do you register
(Tick all that apply)

☒ month of symptom onset
☒ year of symptom onset

Time points for registration

	at inclusion in registry	at start/change of treatment	at follow-up visits	other
Day/month/year of diagnosis	☒	☐	☐	☐
Day/month/year of symptom onset	☒	☐	☐	☐

Which classification criteria are registered?
(Tick all that apply)

☒ ASAS
☒ New York
☒ CASPAR
☒ other

Please specify

diagnosis but not classification criteria is mandatory

Time points for registration

	at inclusion in registry	at start/change of treatment	at follow-up visits	other
ASAS	☒	☐	☐	☐
New York	☒	☐	☐	☐
CASPAR	☒	☐	☐	☐

In which patients can you register ASAS?	⊗ AxSpA
Do you register individual ASAS classification items? (Tick all that apply)	☒ no
Do you register individual New York classification items? (Tick all that apply)	☒ no
Do you register individual CASPAR classification items? (Tick all that apply)	☒ no

Response was added on 2021-12-17 16:12:50.

Axial spondyloarthritis

Which disease status characteristics can be registered in axSpA patients?<br>(Tick all that apply)	☒ swollen joint count<br>☒ tender joint count<br>☒ enthesitis<br>☒ dactylitis<br>☒ physician global<br>☒ BASMI

Time points for registration

	at inclusion in registry	at start/change of treatment	at follow-up visits	other
Swollen joint count	☒	☐	☒	☐
Tender joint count	☒	☐	☒	☐
Enthesitis	☐	☐	☒	☒
Dactylitis	☐	☐	☒	☒
Physician global	☒	☐	☒	☐
BASMI	☒	☐	☒	☐

Please specify (enthesitis)	not mandatory
Please specify (dactylitis)	not mandatory

Swollen joints

How many swollen joint counts can be registered?<br>(Tick all that apply)	☒ 28<br>☒ 66<br>☒ other
Please specify	66/68 is sometimes registrated
Do you register specific location of swollen joints?<br>(Tick all that apply)	☒ yes

Tender joints

How many tender joint counts can be registered?<br>(Tick all that apply)	☒ 28<br>☒ 68
Do you register specific location of tender joints?<br>(Tick all that apply)	☒ yes

Enthesitis

Do you register specific location of enthesitis? (Tick all that apply)	☒ other
Please specify	not mandatory

Dactylitis

How is dactylitis assessed? (Tick all that apply)	☒ other
Other	not mandatory

Physician Global

Please write the wording of the question regarding physician global	VAS scale<br>(translate into english if possible)

BASMI

Are the individidual BASMI components registered?	⊗ yes
Do you register the individual BASMI measurements (cm, degrees)?	⊗ yes
Which BASMI scale is used?	⊗ 3-point

Response was added on 2021-12-17 16:16:15.

Psoriatic arthritis

Which disease status characteristics can be registered in PsA patients? (Tick all that apply)	☒ swollen joint count<br>☒ tender joint count<br>☒ enthesitis<br>☒ dactylitis<br>☒ physician Global<br>☒ BASMI

Time points for registration

	at inclusion in registry	at start/change of treatment	at follow-up visits	other
Swollen joint count	☒	☐	☒	☐
Tender joint count	☒	☐	☒	☐
Enthesitis	☐	☐	☒	☒
Dactylitis	☐	☐	☒	☒
Physician Global	☒	☐	☒	☐
BASMI	☐	☐	☒	☒

Please specify (enthesitis)	not mandatory
Please specify (dactylitis)	not mandatory
Please specify (BASMI)	not mandatory

Swollen joints

How many swollen joint counts can be registered? (Tick all that apply)	☒ 28<br>☒ 66
Do you register specific location of swollen joints? (Tick all that apply)	☒ yes

Tender joints

How many tender joint counts can be registered? (Tick all that apply)	☒ 28<br>☒ 68
Do you register specific location of tender joints? (Tick all that apply)	☒ yes

Entesitis	
Do you register specific location of enthesitis? (Tick all that apply)	☒ no
Please indicate which specific indices that are used, if applicable	not mandatory

Dactylitis	
How is dactylitis assessed? (Tick all that apply)	☒ other
Other	not mandatory (LL)

Physician global	
Please write the wording of the question regarding physician global	VAS scale<br>(Translate into english if possible)

BASMI	
Are the individidual BASMI components registered?	⊗ yes
Do you register the individual BASMI measurements (cm, degrees)?	⊗ yes
Which BASMI scale is used?	⊗ 3-point

Coxitis	
Do you assess coxitis? If yes, please indicate how it is assessed	no

Response was added on 2021-12-17 16:19:22.

Medication

Which therapies are registered in your registry? (Tick all that apply)	☒ biological dmards (bDMARDs), including targeted synthetic dmards (JAK)<br>☒ conventional synthetic dmards (csDMARDs)

bDMARDs

Which year did bDMARD registration begin?	2008
Is it mandatory to register bDMARD therapy?	⊗ yes

csDMARDs

Which year did csDMARD registration begin?	2008
Is it mandatory to register csDMARD therapy?	⊗ no

What information regarding ONGOING medications is registered?

	start date	stop date	temporary start and stop dates	discontinuation reasons	dosage	frequency	administration mode
bDMARDs	☒	☒	☒	☒	☒	☒	☒
csDMARDs	☒	☒	☐	☐	☐	☐	☐

Time points for registration (ONGOING medications)

	at inclusion in registry	at start/change of treatment	at follow-up visits	other
bDMARDs	☒	☒	☒	☐
csDMARDs	☐	☐	☐	☒

Please specify (csDMARDs)	not mandatory to register csDMARDs

What information regarding PAST medications is registered at inclusion in registry?

	start date	stop date	temporary start and stop dates	discontinuation reasons	dosage	name of drug
bDMARDs	☒	☒	☒	☒	☒	☒

csDMARDs	☐	☐	☐	☐	☐	☒

Discontinuation reasons

What are the possible reasons for discontinuation of a bDMARD?
(Tick all that apply)

☒ lack of efficacy
☒ adverse events
☒ remission
☒ pregnancy wish
☒ infection
☒ surgery
☒ cancer
☒ lost to follow-up
☒ death

Is it possible to register multiple reasons for discontinuation?

⊗ yes

How is it decided which is the primary reason for discontinuation?

clinical judgement

Does your registry link to prescription database or other external data sources on prescriptions on a regular basis as input to registry?
(Tick all that apply)

☒ no

Response was added on 2021-12-17 16:23:01.

Patient reported outcomes

Which patient reported outcomes (PROs) are registered in axSpA and/or PsA patients? (Tick all that apply)	☒ BASDAI<br>☒ pain<br>☒ fatigue<br>☒ global disease<br>☒ HAQ
Are any of the PROs registered in either axSpA or PsA only?	⊗ no, all PROs are registered in both diagnoses

Mode of registration

What are the options for registration of PROs? (Tick all that apply)	☒ paper forms<br>☒ on screen in waiting room

Time points for registration

	at inclusion in registry	at start/change of treatment	at follow-up visits	other
BASDAI	☒	☒	☒	☐
Pain	☒	☒	☒	☐
Fatigue	☒	☒	☒	☐
Global disease	☒	☒	☒	☐
HAQ	☒	☒	☒	☐

BASDAI

Are the individidual BASDAI components registered?	⊗ yes

Pain, fatigue and global assessments

Please write the wording of the question relating to pain	Put a mark on the line below (see example) to illustrate the pain due to your disease during the last week. No pain 0______________________100 Unbearable pain<br>(please translate into english if possible)
Please write the wording of the question relating to fatigue	Put a mark on the line below (see example) to illustrate your fatigue due to your disease during the last week. No fatigue 0_____________________100 Unbearable fatigue<br>(please translate into english if possible)

Please write the wording of the question relating to global assessment of disease	Put a mark on the line below (see example) to illustrate the effect of disease activity on your health during the last week. No effect on health 0 __________________100 Unbearable effect on health (please translate into english if possible)

HAQ

Are the individual HAQ items registered?	⊗ yes
Which HAQ versions may be used in your registry? (Tick all that apply)	☒ stanford HAQ DI without adjustment for use of aids and devices

Response was added on 2021-12-17 16:23:28.

Laboratory

Which laboratory test can be registered? (Tick all that apply)	☒ CRP<br>☒ HLA-B27<br>☒ IgM-RF

	at inclusion in registry	at start/change of treatment	at follow-up visits	other
CRP	☒	☒	☒	☐

How are laboratory test results registered? (Tick all that apply)	☒ entered by health care staff

Response was added on 2021-12-17 16:27:46.

Imaging

Which imaging modalities can be registered? (Tick all that apply)	☒ Magnetic Resonance Imaging (MRI)<br>☒ X-ray<br>☒ Ultrasound (US)<br>☒ Computer Tomography (CT)

Information on each image

	date of examination	full image report	image file	scoring system	+/- progression	other
What information on each MRI exam is registered?	☐	☐	☐	☐	☐	☒
What information on each x-ray exam is registered?	☐	☐	☐	☐	☐	☒
What information on each US exam is registered?	☐	☐	☐	☐	☐	☒
What information on each CT exam is registered?	☐	☐	☐	☐	☐	☒

Please specify (MRI)	not mandatory
Please specify (x-ray)	not mandatory
Please specify (US)	not mandatory
Please specify (CT)	not mandatory

MRI

Which anatomical regions can be registered? (Tick all that apply)	☒ spine MRI<br>☒ sacroiliac joint MRI<br>☒ other
Please specify	not mandatory

X-ray

Which anatomical regions can be registered? (Tick all that apply)	☒ spine radiographs<br>☒ sacroiliac joint radiographs<br>☒ hands and feet<br>☒ other
Please specify	not mandatory

Ultrasound

Please indicate which anatomical regions that can be registered	all joints, same as DANBIO

CT

Please indicate which anatomical regions that can be registered?	not done

Frequency of image registration

Please comment on the frequency of registration of the various image modalities, eg. at specified time points, as needed, in connection with research projects or other.	Clinical judgement, not mandatory

Response was added on 2021-12-17 16:28:55.

Comorbidities

Which extraarticular manifestations and comorbid conditions are registered?<br>(Tick all that apply)	☒ none
Do you use ICD-10 codes for registration?	⊗ yes
General comments	Is it possible to make linkage with other registries incl comorbities

Response was added on 2021-12-17 16:30:28.

Lifestyle

Which lifestyle parameters are registered? (Tick all that apply)	☒ smoking<br>☒ other
Please specify	not mandatory

Time points for registration

	at inclusion in registry	at start/change of treatment	at follow-up visits	other
Smoking	☒	☐	☒	☐

Smoking

How is smoking status characterised? (Tick all that apply)	☒ current<br>☒ former<br>☒ never
Do you register a start date? (Tick all that apply)	☒ for current smokers<br>☒ for former smokers
Do you register a stop date for former smokers?	⊗ yes
How is average number of smoked cigarettes registered? (Tick all that apply)	☒ number of daily cigarettes
General comments	Smoking is registered as in DANBIO

Response was added on 2021-12-17 16:31:24.

Safety

Can you register adverse events in your registry? (Tick all that apply)	☒ yes, directly into registry<br>☒ only by linkage to relevant registries
Is it mandatory to register adverse events through your registry?	⊗ yes
Which adverse events are registered in your registry? (Tick all that apply)	☒ serious adverse events?

	date of event	MeddRA	ICD10-code	outcome	other
Serious AE	☒	☐	☒	☒	☐

Participant information

Record ID	15
Name	Catalin Codreanu
Registry	RRBR
E-mail	ccodreanu01@gmail.com
Deltager	⊗ ja

Response was added on 2021-12-29 10:20:03.

General registry information	
What is the status of your registry?	⊗ running and including patients

Coverage	
Please estimate how many (percentage) of the eligible spondyloarthritis patients in your country, that are registered	100
Please estimate how many (percentage) of the eligible psoriatic arthritis patients in your country, that are registered	100
How did you obtain the coverage estimate above? (Tick all that apply)	☒ other
Please specify	In Romania, inclusion of patients treated with biologics in the Registry is mandatory. We estimate an almost 100% inclusion rate of patients on current biological treatment.
Which institutions/organisations can include patients in your registry? (Tick all that apply)	☒ private rheumatology practices<br>☒ departments of rheumatology at hospitals<br>☒ departments of rheumatology at university hospitals<br>☒ other
Please give an estimate of how many private rheumatology practices that include patients in your registry	5-10
Please give an estimate of how many departments of rheumatology at hospitals (not including university hospitals) that include patients in your registry	35
Please give an estimate of how many departments of rheumatology at university hospitals that include patients in your registry	15
Please specify	In Romania, pateints are included in the Registry by the treating psysician, so number of departments is not relevant. We have 372 rheumatologists, 27 rehabilitation specialists and 18 internal medicine specialists including patients in the Registry.
When is the data registered? (Tick all that apply)	☒ at pre-specified registry visits

What is the schedule for pre-specified registry visits?	usually each 6 months
Is it possible to register visits outside of the pre-specified visit schedule, eg. if a patient has a flare?	⊗ yes

Ethics

Is approval from a local ethics committee needed for a study on de-identified data (eg. a EuroSpA upload)?<br>(Tick all that apply)	☒ yes
Do patients need to sign informed consent to be included in your registry?<br>(Tick all that apply)	☒ yes
Are any additional local approvals needed for a study on de-identified data (eg. a EuroSpA upload)?<br>(Tick all that apply)	☒ yes
Please specify which additional approvals are needed	Scientific Committee of the RRBR

Funding

How is the registry funded?<br>(Tick all that apply)	☒ from research grants<br>☒ industry
Please estimate the percentage of funds coming from research grants	50<br>((0-100%))
Please estimate the percentage of funds coming from industry, eg. pharmaceutical company	50<br>((0-100%))
The percentages add correctly up to a 100%	
What is the basis of participation by a clinic/department/office in the registry?<br>(Tick all that apply)	☒ mandatory
Is the clinic/department/office financially compensated for registration?	⊗ no

Inclusion criteria

What event triggers the inclusion of a patient into the registry?<br>(Tick all that apply)	☒ new treatment
Which criteria do you base the inclusion on?<br>(Tick all that apply)	☒ treatment
Is a minimum age required for inclusion?	☒ yes, 18 years or above

Do patients need to be treated with biological DMARDs (including targeted synthetic DMARDs) to be included in the registry?	⊗ yes
Have the inclusion criteria changed over time?	⊗ no

Response was added on 2021-12-29 10:20:23.

Data management

What are the options for data entry?<br>(Tick all that apply)	☒ paper based<br>☒ electronic
Are the data fields in your registry interactive, such that invalid or unprobable data is flagged when entered (edit checks)<br>(Tick all that apply)	☒ yes
Since when (year) has the data fields been interactive?	2013
How does your registry retain data management services?<br>(Tick all that apply)	☒ external company<br>☒ person employed as data manager<br>☒ a researcher/administrative personal does data management beside other duties
Which is the background of your data manager(s)?<br>(Tick all that apply)	☒ clinical<br>☒ data science/biostatistician/technical
Which are the most commonly used data analysis software/programming languages in your organization?<br>(Tick all that apply)	☒ excel<br>☒ SPSS<br>☒ other
Please specify	Java
How is the data stored in the registry?<br>(Tick all that apply)	☒ a relational database framework (like SQL)
Which are the main data formats used for raw data extractions?<br>(Tick all that apply)	☒ excel<br>☒ SQL-output
Where is your data collection platform hosted?<br>(Tick all that apply)	☒ external company
Who maintains your data collection platform?<br>(Tick all that apply)	☒ external company
How frequently is the database updated with the latest information?<br>(Tick all that apply)	☒ real-time
Is linkage to other databases or registries possible?	⊗ yes
Which registries can be linked to?<br>(Tick all that apply)	☒ prescription registry<br>☒ electronic medical records

General comments	The registry can be linked to other databases, but the linkage is not active at present moment. Additional developments are needed.

Response was added on 2021-12-29 10:20:37.

Demography

Please indicate which of the following variables are collected in your registry
(Tick all that apply)

- ☒ age (year of birth)
- ☒ sex
- ☒ weight
- ☒ height
- ☒ death

Time points for registration

	at inclusion in registry	at start/change of treatment	at follow-up visits	other
Weight	☒	☒	☒	☐
Height	☒	☐	☐	☐

How is vital status registered
(Tick all that apply)

- ☒ by healthstaff, manually

Diagnosis

How is a diagnosis registered?
(Tick all that apply)

- ☒ classification criteria

Do you register
(Tick all that apply)

- ☒ month of diagnosis
- ☒ year of diagnosis

Do you register
(Tick all that apply)

- ☒ month of symptom onset
- ☒ year of symptom onset

Time points for registration

	at inclusion in registry	at start/change of treatment	at follow-up visits	other
Day/month/year of diagnosis	☒	☐	☐	☐
Day/month/year of symptom onset	☒	☐	☐	☐

Which classification criteria are registered?
(Tick all that apply)

- ☒ ASAS
- ☒ New York
- ☒ CASPAR

Time points for registration

	at inclusion in registry	at start/change of treatment	at follow-up visits	other
ASAS	☒	☐	☐	☐
New York	☒	☐	☐	☐
CASPAR	☒	☐	☐	☐

In which patients can you register ASAS?	⊗ AxSpA
Do you register individual ASAS classification items? (Tick all that apply)	☒ yes
Do you register individual New York classification items? (Tick all that apply)	☒ yes
Do you register individual CASPAR classification items? (Tick all that apply)	☒ yes

Response was added on 2021-12-29 10:20:49.

Axial spondyloarthritis

Which disease status characteristics can be registered in axSpA patients? (Tick all that apply)	☒ swollen joint count<br>☒ tender joint count<br>☒ enthesitis<br>☒ dactylitis<br>☒ other
Please specify	BASDAI, ASDAS

Time points for registration

	at inclusion in registry	at start/change of treatment	at follow-up visits	other
Swollen joint count	☒	☒	☒	☐
Tender joint count	☒	☒	☒	☐
Enthesitis	☒	☒	☒	☐
Dactylitis	☒	☒	☒	☐

Swollen joints

How many swollen joint counts can be registered? (Tick all that apply)	☒ 66
Do you register specific location of swollen joints? (Tick all that apply)	☒ yes

Tender joints

How many tender joint counts can be registered? (Tick all that apply)	☒ 68
Do you register specific location of tender joints? (Tick all that apply)	☒ yes

Enthesitis

Do you register specific location of enthesitis? (Tick all that apply)	☒ no, only as a total count<br>☒ other
Please specify	enthesitis are registered as a total number and it is possible to describe the location (optional)

Dactylitis

How is dactylitis assessed? (Tick all that apply)	☒ as part of classification criteria

Coxitis

Do you assess coxitis? If yes, please indicate how it is assessed	Clinically, in the tender joint count and by imaging (x-ray/MRI)

Response was added on 2021-12-29 10:21:05.

Psoriatic arthritis

Which disease status characteristics can be registered in PsA patients? (Tick all that apply)	☒ swollen joint count<br>☒ tender joint count<br>☒ enthesitis<br>☒ dactylitis<br>☒ skin<br>☒ physician Global<br>☒ other
Please specify	DAPSA

Time points for registration

	at inclusion in registry	at start/change of treatment	at follow-up visits	other
Swollen joint count	☒	☒	☒	☐
Tender joint count	☒	☒	☒	☐
Enthesitis	☒	☒	☒	☐
Dactylitis	☒	☒	☒	☐
Skin	☒	☒	☒	☐
Physician Global	☒	☒	☒	☐

Swollen joints

How many swollen joint counts can be registered? (Tick all that apply)	☒ 66
Do you register specific location of swollen joints? (Tick all that apply)	☒ yes

Tender joints

How many tender joint counts can be registered? (Tick all that apply)	☒ 68
Do you register specific location of tender joints? (Tick all that apply)	☒ yes

Entesitis

Do you register specific location of enthesitis? (Tick all that apply)	☒ yes
Which locations are registered? (Tick all that apply)	☒ medial femoral condyle<br>☒ achilles tendon insertion<br>☒ supraspinatus insertion
Please indicate which specific indices that are used, if applicable	Leeds

Dactylitis

How is dactylitis assessed? (Tick all that apply)	☒ as part of classification criteria<br>☒ as a count

Skin

Which instruments are used for registering skin involvment in PsA? (Tick all that apply)	☒ PASI

Physician global

Please write the wording of the question regarding physician global	Please rate how much the disease is globally affecting your patient, taking into account all the aspects of the disease (e.g. psoriasis and arthritis) over the past week<br>(Translate into english if possible)

Coxitis

Do you assess coxitis? If yes, please indicate how it is assessed	Clinically in TJC and imaging (x-ray /MRI)

Response was added on 2021-12-29 10:21:21.

Medication

Which therapies are registered in your registry? (Tick all that apply)	☒ biological dmards (bDMARDs), including targeted synthetic dmards (JAK)<br>☒ conventional synthetic dmards (csDMARDs)<br>☒ glucocorticoids<br>☒ NSAIDs<br>☒ medication for comorbidity

bDMARDs

Which year did bDMARD registration begin?	2013
Is it mandatory to register bDMARD therapy?	⊗ yes

csDMARDs

Which year did csDMARD registration begin?	2013
Is it mandatory to register csDMARD therapy?	⊗ yes

Glucocorticoids

Which year did glucocorticoid registration begin?	2013
Is it mandatory to register glucocorticoid therapy?	⊗ no
Is the mode of administration registered? (Tick all that apply)	☒ not registered

NSAIDs

Which year did NSAID registration begin?	2013
Is it mandatory to register NSAID therapy?	⊗ yes

Medicaton for comorbidities

What types of medical therapy for comorbidities are registered?	all

What information regarding ONGOING medications is registered?

	start date	stop date	temporary start and stop dates	discontinuation reasons	dosage	frequency	administration mode
bDMARDs	☒	☒	☐	☒	☒	☒	☒
csDMARDs	☒	☒	☐	☒	☒	☒	☐
NSAIDs	☒	☒	☐	☒	☒	☒	☐

Time points for registration (ONGOING medications)

	at inclusion in registry	at start/change of treatment	at follow-up visits	other
bDMARDs	☒	☒	☒	☐
csDMARDs	☒	☒	☒	☐
glucocorticoids	☒	☐	☒	☐
NSAIDs	☒	☐	☒	☐
medication for comorbidity	☒	☐	☒	☐

What information regarding PAST medications is registered at inclusion in registry?

	start date	stop date	temporary start and stop dates	discontinuation reasons	dosage	name of drug
bDMARDs	☒	☒	☐	☒	☒	☒
csDMARDs	☒	☒	☐	☒	☒	☒
NSAIDs	☒	☒	☐	☒	☒	☒

Discontinuation reasons

What are the possible reasons for discontinuation of a bDMARD?
(Tick all that apply)

☒ lack of efficacy
☒ adverse events
☒ remission
☒ pregnancy wish
☒ infection
☒ surgery
☒ cancer
☒ lost to follow-up
☒ death

Is it possible to register multiple reasons for discontinuation?

⊗ no

Does your registry link to prescription database or other external data sources on prescriptions on a regular basis as input to registry?
(Tick all that apply)

☒ no

General comments

Registration of glucocorticoids is done through categories (treatment since last visit, dosage over/under 7,5 mg daily) not actual doses.
Treatment for comorbidity: yes/no but not mandatory.
Past NSAIDs: only for AxSpA

Response was added on 2021-12-29 10:21:34.

Patient reported outcomes

Which patient reported outcomes (PROs) are registered in axSpA and/or PsA patients? (Tick all that apply)	☒ BASDAI<br>☒ pain<br>☒ global disease<br>☒ HAQ<br>☒ EQ-5D
Are any of the PROs registered in either axSpA or PsA only?	⊗ yes
Which PROs are registered uniquely in either axSpA or PsA?	axSpA: BASDAI; PsA: pain score

Mode of registration

What are the options for registration of PROs? (Tick all that apply)	☒ paper forms

Time points for registration

	at inclusion in registry	at start/change of treatment	at follow-up visits	other
BASDAI	☒	☒	☒	☐
Pain	☒	☒	☒	☐
Global disease	☒	☒	☒	☐
HAQ	☐	☐	☐	☒
EQ-5D	☐	☐	☐	☒

Please specify (HAQ)	optional anytime
Please specify (EQ-5D)	optional anytime

BASDAI

Are the individidual BASDAI components registered?	⊗ yes

Pain, fatigue and global assessments

Please write the wording of the question relating to pain	Please rate the level of your joint pain related to PsA during the last week (please translate into english if possible)
Please write the wording of the question relating to global assessment of disease	Please rate how much the disease is globally affecting you, taking into account all the aspects of the disease (e.g. psoriasis and arthritis) over the past week (please translate into english if possible)

HAQ

Are the individual HAQ items registered?	⊗ no
Which HAQ versions may be used in your registry? (Tick all that apply)	☒ stanford HAQ DI with adjustment for use of aids and devices

EQ-5D

Are the individual EQ-5D items registered?	⊗ yes
Which EQ-5D version is used?	EQ-5D-3L
Which algorithm is used?	according to EuroQol

Response was added on 2021-12-29 10:21:42.

Laboratory

Which laboratory test can be registered?
(Tick all that apply)

- ☒ ESR
- ☒ CRP
- ☒ hemoglobin
- ☒ ALAT
- ☒ creatinine
- ☒ Hba1c
- ☒ cholesterol
- ☒ HLA-B27
- ☒ IgM-RF

Time points for registration

	at inclusion in registry	at start/change of treatment	at follow-up visits	other
ESR	☒	☒	☒	☐
CRP	☒	☒	☒	☐
Hemoglobin	☒	☒	☒	☐
ALAT	☒	☒	☒	☐
Creatinine	☒	☒	☒	☐
Hba1c	☐	☐	☐	☒
Cholesterol	☐	☐	☐	☒

Please specify (Hba1c) — optional anytime, when available

Please specify (cholesterol) — optional anytime, when available

Cholesterol

Which specific cholesterol types are registered?
(Tick all that apply)

- ☒ total cholesterol
- ☒ LDL
- ☒ HDL
- ☒ triglycerid

How are laboratory test results registered?
(Tick all that apply)

- ☒ entered by health care staff

Response was added on 2021-12-29 10:21:48.

Imaging

Which imaging modalities can be registered? (Tick all that apply)

☒ Magnetic Resonance Imaging (MRI)
☒ X-ray
☒ DXA
☒ Ultrasound (US)

Information on each image

	date of examination	full image report	image file	scoring system	+/- progression	other
What information on each MRI exam is registered?	☒	☒	☐	☐	☐	☐
What information on each x-ray exam is registered?	☒	☒	☐	☒	☐	☐
What information on each DXA exam is registered?	☒	☒	☐	☐	☐	☐
What information on each US exam is registered?	☒	☒	☐	☐	☐	☐

MRI

Which anatomical regions can be registered? (Tick all that apply)

☒ spine MRI
☒ sacroiliac joint MRI

X-ray

Which anatomical regions can be registered? (Tick all that apply)

☒ spine radiographs
☒ sacroiliac joint radiographs
☒ hands and feet

Which scoring system? According to NY criteria for sakroileitis

DEXA

Which anatomical regions can be registered? (Tick all that apply)

☒ lumbar spine
☒ femoral neck

Ultrasound

Please indicate which anatomical regions that can be registered	at enthesitis sites

Frequency of image registration

Please comment on the frequency of registration of the various image modalities, eg. at specified time points, as needed, in connection with research projects or other.	at start of biological treatment and afterwards, as clinically needed

Response was added on 2021-12-29 10:22:00.

Comorbidities

Which extraarticular manifestations and comorbid conditions are registered?<br>(Tick all that apply)	☒ uveitis<br>☒ psoriasis<br>☒ inflammatory bowel disease (IBD)<br>☒ ischemic heart disease (IHD)<br>☒ cerebrovascular disease (CVD)<br>☒ hypertension<br>☒ diabetes (DM)<br>☒ dyslipidemia<br>☒ osteoporosis<br>☒ chronic kidney insufficiency (CKI)<br>☒ chronic liver disease, eg. hepatitis, cirrhosis<br>☒ solid cancer<br>☒ hematological cancer<br>☒ depression<br>☒ tuberculosis (TB)<br>☒ fibromyalgia<br>☒ other
Please specify	any other

Time points for registration

	at inclusion in registry	at start/change of treatment	at follow-up visits	other
uveitis	☒	☒	☒	☐
psoriasis	☒	☒	☒	☐
IBD	☒	☒	☒	☐
IHD	☒	☒	☒	☐
CVD	☒	☒	☒	☐
hypertension	☒	☒	☒	☐
DM	☒	☒	☒	☐
dyslipidemia	☒	☒	☒	☐
osteoporosis	☒	☒	☒	☐
CKI	☒	☒	☒	☐
chronic liver disease	☒	☒	☒	☐
solid cancer	☒	☒	☒	☐
hematological cancer	☒	☒	☒	☐
depression	☒	☒	☒	☐
TB	☒	☒	☒	☐

fibromyalgia	☒	☒	☒	☐
other	☒	☒	☒	☐

Mode of registration - how are comorbid conditions registered?

	patient-reported	by health-staff	linkage from other registry
uveitis	☐	☒	☐
psoriasis	☐	☒	☐
IBD	☐	☒	☐
IHD	☐	☒	☐
CVD	☐	☒	☐
hypertension	☐	☒	☐
DM	☐	☒	☐
dyslipidemia	☐	☒	☐
osteoporosis	☐	☒	☐
CKI	☐	☒	☐
chronic liver disease	☐	☒	☐
solid cancer	☐	☒	☐
hematological cancer	☐	☒	☐
depression	☐	☒	☐
TB	☐	☒	☐
fibromyalgia	☐	☒	☐
other	☐	☒	☐

Do you use ICD-10 codes for registration? ⊗ no

Response was added on 2021-12-29 10:22:09.

Lifestyle

Which lifestyle parameters are registered? (Tick all that apply)	☒ smoking

Time points for registration

	at inclusion in registry	at start/change of treatment	at follow-up visits	other
Smoking	☒	☐	☒	☐

Smoking

How is smoking status characterised? (Tick all that apply)	☒ current<br>☒ former<br>☒ never
Do you register a start date? (Tick all that apply)	☒ no date is registered
Do you register a stop date for former smokers?	⊗ No
How is average number of smoked cigarettes registered? (Tick all that apply)	☒ number of daily cigarettes

Response was added on 2021-12-29 10:22:14.

Safety

Can you register adverse events in your registry? (Tick all that apply)	☒ yes, directly into registry
Is it mandatory to register adverse events through your registry?	⊗ no
Which adverse events are registered in your registry? (Tick all that apply)	☒ non-serious adverse events<br>☒ serious adverse events?

Information on adverse events

	date of event	MeddRA	ICD10-code	outcome	other
Non serious AE	☒	☐	☐	☒	☐
Serious AE	☒	☐	☐	☒	☐

Participant information

Record ID	16
Name	Isabel Castrejon
Registry	BIOBADASER
E-mail	isabelcastrejonf@gmail.com
Deltager	⊗ ja

Response was added on 2022-02-03 18:13:36.

General registry information

What is the status of your registry?	⊗ running and including patients
Which institutions/organisations can include patients in your registry?<br>(Tick all that apply)	☒ departments of rheumatology at university hospitals
Please give an estimate of how many departments of rheumatology at university hospitals that include patients in your registry	28
When is the data registered?<br>(Tick all that apply)	☒ at routine visits
Are all routine visits registered in your registry - or only some?	⊗ only some visits

Ethics

Is approval from a local ethics committee needed for a study on de-identified data (eg. a EuroSpA upload)?<br>(Tick all that apply)	☒ yes
Do patients need to sign informed consent to be included in your registry?<br>(Tick all that apply)	☒ yes
Are any additional local approvals needed for a study on de-identified data (eg. a EuroSpA upload)?<br>(Tick all that apply)	☒ no

Funding

How is the registry funded?<br>(Tick all that apply)	☒ from public sector, eg. state or other<br>☒ industry
Please estimate the percentage of funds coming from public sector, eg. state or other	10<br>((0-100%))
Please estimate the percentage of funds coming from industry, eg. pharmaceutical company	90<br>((0-100%))
The percentages add correctly up to a 100%	
What is the basis of participation by a clinic/department/office in the registry?<br>(Tick all that apply)	☒ voluntary

Is the clinic/department/office financially compensated for registration?	⊗ yes

Inclusion criteria

What event triggers the inclusion of a patient into the registry?<br>(Tick all that apply)	☒ new treatment
Which criteria do you base the inclusion on?<br>(Tick all that apply)	☒ treatment
Is a minimum age required for inclusion?	☒ yes, 18 years or above
Do patients need to be treated with biological DMARDs (including targeted synthetic DMARDs) to be included in the registry?	⊗ yes
Have the inclusion criteria changed over time?	⊗ no
General comments	The finantial compesantion is to cover the data entry personnel at each centre.

Response was added on 2022-02-03 18:18:40.

Data management

What are the options for data entry?<br>(Tick all that apply)	☒ electronic
Are the data fields in your registry interactive, such that invalid or unprobable data is flagged when entered (edit checks)<br>(Tick all that apply)	☒ yes
Please describe any other data validation procedures that you may use	regular external monitoring
How does your registry retain data management services?<br>(Tick all that apply)	☒ a researcher/administrative personal does data management beside other duties
Which is the background of your data manager(s)?<br>(Tick all that apply)	☒ data science/biostatistician/technical
Which are the most commonly used data analysis software/programming languages in your organization?<br>(Tick all that apply)	☒ stata
How is the data stored in the registry?<br>(Tick all that apply)	☒ a relational database framework (like SQL)
Which are the main data formats used for raw data extractions?<br>(Tick all that apply)	☒ excel
Where is your data collection platform hosted?<br>(Tick all that apply)	☒ external company
Who maintains your data collection platform?<br>(Tick all that apply)	☒ data manager<br>☒ researcher/epidemiologist<br>☒ other
Other	The Spanish Society of Rheumatology Research Foundation
How frequently is the database updated with the latest information?<br>(Tick all that apply)	☒ real-time
Is linkage to other databases or registries possible?	⊗ no
General comments	As far as I know we did not linked BIOBADASER to other registries it would be possible

Response was added on 2022-02-03 18:21:06.

Demography

Please indicate which of the following variables are collected in your registry
(Tick all that apply)

☒ age (year of birth)
☒ sex
☒ ethnicity
☒ weight
☒ height
☒ death

Time points for registration

	at inclusion in registry	at start/change of treatment	at follow-up visits	other
Weight	☒	☐	☐	☐
Height	☒	☐	☐	☐

How is vital status registered
(Tick all that apply)

☒ by healthstaff, manually

Diagnosis

How is a diagnosis registered?
(Tick all that apply)

☒ other diagnostic categories

Specify which diagnostic categories

according to clinical diagnosis

Do you register
(Tick all that apply)

☒ date for diagnosis
☒ other

Please specify

if exact date is not available an aproximation

Do you register
(Tick all that apply)

☒ date for symptom onset

Time points for registration

	at inclusion in registry	at start/change of treatment	at follow-up visits	other
Day/month/year of diagnosis	☒	☐	☐	☐
Day/month/year of symptom onset	☒	☐	☐	☐

Which classification criteria are registered?
(Tick all that apply)

☒ none

Response was added on 2022-02-03 18:26:17.

Axial spondyloarthritis

Which disease status characteristics can be registered in axSpA patients? (Tick all that apply)	☒ swollen joint count<br>☒ tender joint count

Time points for registration

	at inclusion in registry	at start/change of treatment	at follow-up visits	other
Swollen joint count	☒	☐	☐	☒
Tender joint count	☒	☐	☐	☒

Please specify (swollen joint count)	when change of therapy or at least once a year
Please specify (tender joint count)	same

Swollen joints

How many swollen joint counts can be registered? (Tick all that apply)	☒ 28
Do you register specific location of swollen joints? (Tick all that apply)	☒ no, just joint counts

Tender joints

How many tender joint counts can be registered? (Tick all that apply)	☒ 28
Do you register specific location of tender joints? (Tick all that apply)	☒ no, just joint counts

Coxitis

Do you assess coxitis? If yes, please indicate how it is assessed	no

Response was added on 2022-02-03 18:27:43.

Psoriatic arthritis

Which disease status characteristics can be registered in PsA patients? (Tick all that apply)	☒ swollen joint count<br>☒ tender joint count<br>☒ other
Please specify	BASDAI and ASDAS-CRP if axial manifestations

Time points for registration

	at inclusion in registry	at start/change of treatment	at follow-up visits	other
Swollen joint count	☒	☐	☒	☐
Tender joint count	☒	☐	☒	☐

Swollen joints

How many swollen joint counts can be registered? (Tick all that apply)	☒ 28
Do you register specific location of swollen joints? (Tick all that apply)	☒ no, just joint counts

Tender joints

How many tender joint counts can be registered? (Tick all that apply)	☒ 28
Do you register specific location of tender joints? (Tick all that apply)	☒ no, just joint counts

Coxitis

Do you assess coxitis? If yes, please indicate how it is assessed	no

Response was added on 2022-02-03 18:39:28.

Medication

Which therapies are registered in your registry? (Tick all that apply)	☒ biological dmards (bDMARDs), including targeted synthetic dmards (JAK)<br>☒ conventional synthetic dmards (csDMARDs)<br>☒ glucocorticoids

bDMARDs

Which year did bDMARD registration begin?	2000
Is it mandatory to register bDMARD therapy?	⊗ yes

csDMARDs

Which year did csDMARD registration begin?	2000
Is it mandatory to register csDMARD therapy?	⊗ yes

Glucocorticoids

Which year did glucocorticoid registration begin?	2000
Is it mandatory to register glucocorticoid therapy?	⊗ yes
Is the mode of administration registered? (Tick all that apply)	☒ oral<br>☒ intraarticular
Are specific locations of injected joints registered?	⊗ yes

What information regarding ONGOING medications is registered?

	start date	stop date	temporary start and stop dates	discontinuation reasons	dosage	frequency	administration mode
bDMARDs	☒	☒	☐	☒	☒	☐	☐

Time points for registration (ONGOING medications)

	at inclusion in registry	at start/change of treatment	at follow-up visits	other
bDMARDs	☒	☒	☒	☒

Please specify (bDMARDs)	one year follow-up

What information regarding PAST medications is registered at inclusion in registry?

	start date	stop date	temporary start and stop dates	discontinuation reasons	dosage	name of drug
bDMARDs	☒	☒	☒	☒	☒	☒

Discontinuation reasons

What are the possible reasons for discontinuation of a bDMARD?<br>(Tick all that apply)	☒ lack of efficacy<br>☒ adverse events<br>☒ remission<br>☒ pregnancy wish<br>☒ infection<br>☒ surgery<br>☒ cancer<br>☒ lost to follow-up<br>☒ death<br>☒ other
Please specify	Discontinuation for non-medical reasons
Is it possible to register multiple reasons for discontinuation?	⊗ no
Does your registry link to prescription database or other external data sources on prescriptions on a regular basis as input to registry?<br>(Tick all that apply)	☒ no

Response was added on 2022-02-03 19:05:56.

Patient reported outcomes

Which patient reported outcomes (PROs) are registered in axSpA and/or PsA patients? (Tick all that apply)	☒ BASDAI<br>☒ global disease
Are any of the PROs registered in either axSpA or PsA only?	⊗ no, all PROs are registered in both diagnoses

Mode of registration

What are the options for registration of PROs? (Tick all that apply)	☒ paper forms<br>☒ other
Please specify	Variable according to each clinic, mostly in paper or during clinical encounter by doctor

Time points for registration

	at inclusion in registry	at start/change of treatment	at follow-up visits	other
BASDAI	☒	☐	☒	☐
Global disease	☒	☐	☒	☐

BASDAI

Are the individidual BASDAI components registered?	⊗ no, only the composite score
Please write the wording of the question relating to global assessment of disease	no specific wording, depending on each center (please translate into english if possible)

Response was added on 2022-02-03 19:11:36.

Laboratory

Which laboratory test can be registered? (Tick all that apply)

- ☒ ESR
- ☒ CRP
- ☒ HLA-B27
- ☒ IgM-RF

Time points for registration

	at inclusion in registry	at start/change of treatment	at follow-up visits	other
ESR	☒	☐	☒	☐
CRP	☒	☐	☒	☐

How are laboratory test results registered? (Tick all that apply)

- ☒ entered by health care staff

Response was added on 2022-02-03 19:18:47.

Frequency of image registration

Please comment on the frequency of registration of the various image modalities, eg. at specified time points, as needed, in connection with research projects or other.	No image registration

Response was added on 2022-02-03 19:21:49.

Comorbidities

Which extraarticular manifestations and comorbid conditions are registered?
(Tick all that apply)

- ☒ uveitis
- ☒ psoriasis
- ☒ inflammatory bowel disease (IBD)
- ☒ ischemic heart disease (IHD)
- ☒ cerebrovascular disease (CVD)
- ☒ hypertension
- ☒ diabetes (DM)
- ☒ dyslipidemia
- ☒ osteoporosis
- ☒ chronic kidney insufficiency (CKI)
- ☒ chronic liver disease, eg. hepatitis, cirrhosis
- ☒ solid cancer
- ☒ hematological cancer
- ☒ depression
- ☒ tuberculosis (TB)
- ☒ fibromyalgia

Time points for registration

	at inclusion in registry	at start/change of treatment	at follow-up visits	other
uveitis	☒	☐	☐	☐
psoriasis	☒	☐	☐	☐
IBD	☒	☐	☐	☐
IHD	☒	☐	☐	☐
CVD	☒	☐	☐	☐
hypertension	☒	☐	☐	☐
DM	☒	☐	☐	☐
dyslipidemia	☒	☐	☐	☐
osteoporosis	☒	☐	☐	☐
CKI	☒	☐	☐	☐
chronic liver disease	☒	☐	☐	☐
solid cancer	☒	☐	☐	☐
hematological cancer	☒	☐	☐	☐
depression	☒	☐	☐	☐
TB	☒	☐	☐	☐
fibromyalgia	☒	☐	☐	☐

Mode of registration - how are comorbid conditions registered?

	patient-reported	by health-staff	linkage from other registry
uveitis	☐	☒	☐
psoriasis	☐	☒	☐
IBD	☐	☒	☐
IHD	☐	☒	☐
CVD	☐	☒	☐
hypertension	☐	☒	☐
DM	☐	☒	☐
dyslipidemia	☐	☒	☐
osteoporosis	☐	☒	☐
CKI	☐	☒	☐
chronic liver disease	☐	☒	☐
solid cancer	☐	☒	☐
hematological cancer	☐	☒	☐
depression	☐	☒	☐
TB	☐	☒	☐
fibromyalgia	☐	☒	☐

Do you use ICD-10 codes for registration?	⊗ no
General comments	Charlson index with possibility to add additonal comorbidities

Response was added on 2022-02-03 19:24:05.

Lifestyle

Which lifestyle parameters are registered? (Tick all that apply)	☒ smoking

Time points for registration

	at inclusion in registry	at start/change of treatment	at follow-up visits	other
Smoking	☒	☐	☒	☐

Smoking

How is smoking status characterised? (Tick all that apply)	☒ current<br>☒ former<br>☒ never<br>☒ other
Please specify	non-specified
Do you register a start date? (Tick all that apply)	☒ for current smokers
Do you register a stop date for former smokers?	⊗ No
How is average number of smoked cigarettes registered? (Tick all that apply)	☒ not registered

Response was added on 2022-02-03 19:24:45.

Safety

Can you register adverse events in your registry? (Tick all that apply)	☒ yes, directly into registry
Is it mandatory to register adverse events through your registry?	⊗ yes
Which adverse events are registered in your registry? (Tick all that apply)	☒ non-serious adverse events<br>☒ serious adverse events?

Information on adverse events

	date of event	MeddRA	ICD10-code	outcome	other
Non serious AE	☒	☒	☐	☒	☐
Serious AE	☒	☒	☐	☒	☐
