# Supplementary material for: Commonalities and differences in set-up and data collection across European spondyloarthritis registries — results from the EuroSpA collaboration
Source: Arthritis Res Ther. 2023 Oct 19;25:205. doi: 10.1186/s13075-023-03184-7 (PMC10585911; doi:10.1186/s13075-023-03184-7)
Supplement: Supplementary file 2 — Additional file 2. Interview guide – data mapping. S1. Data availability as reported by the registries. S2. Overview of selected patient reported outcomes in PsA across registries. [file 13075_2023_3184_MOESM2_ESM.docx]

**Interview guide – data mapping**

1. **General registry information**

Coverage: Characterise an eligible patient:

How precise are the estimates of institutions/organisations that include patients into the registry?

If possible, provide an estimate of the total number of institutions/organisations (denominator) in your country.

How many yearly visits are registered on average?

Ethics: Has the registry been approved by any authority? Which?

Funding: If registry is funded by industry, how many different sources does the funding come from? Are the contributions equally distributed?

What is the funding used for?

1. **Axial SpA and PsA**

Is there a way to differentiate between nrAxSpA and AS in the registry?

Physician global:

Recall period?

1. **Medication**

Review of replies in ongoing/past medications

1. **PRO**

Assessment: If paper forms: How are they entered into the registry?

Pain, fatigue and global assessments:

Wording?

Recall period?

1. **Comorbidities**

Mode of registration:

How are comorbid conditions assessed? Eg. through medical record review, interview with patient or other?

1. **Registry specific questions**

See attached PDFs.

S1. Data availability as reported by the registries.

| Variables | Number of registries |
| --- | --- |
| **Lifestyle** |  |
| Physical activity | 4 |
| **Medications** |  |
| Intramuscular glucocorticoids | 4 |
| Intraarticular glucocorticoids | 5 |
| **Patient-reported outcomes** |  |
| EQ-5D | 9 |
| **Comorbidities** |  |
| Hypertension | 12 |
| Dyslipidaemia | 10 |
| Osteoporosis | 11 |
| Chronic liver disease | 13 |
| Solid cancer | 10 |
| Haematological cancer | 10 |
| Depression | 11 |
| Tuberculosis | 11 |
| Fibromyalgia | 7 |
| **Imaging** |  |
| X-ray | 7 |
| Magnetic Resonance Imaging | 6 |
| Ultrasound | 4 |
| Dual energy | 3 |
| Computer tomography | 2 |
| **Safety** |  |
| Serious adverse events | 15 |
| Non-serious adverse events | 13 |

S2. Overview of selected patient reported outcomes in PsA across registries.

| Registry | Patient global assessment | | Patient pain assessment | | Patient fatigue assessment | | BASDAI/BASFI | |
| --- | --- | --- | --- | --- | --- | --- | --- | --- |
|  | Wording* | Scale** | Wording* | Scale** | Wording* | Scale** | Registered | Scale** |
| ATTRA | Please indicate below how you feel when you consider all the ways in which your illness now affects you | VAS and NRS (0-100) | How much pain has your illness caused you DURING THE PAST WEEK? | NRS (0-100) | How much of a problem has unsual fatigue been for you DURING THE PAST WEEK? | NRS (0-100) | no/no |  |
| DANBIO | How does the arthrits affect your overall life at the moment? | VAS | How much pain due to arthritis do you have at the moment? | VAS | How tired are you at the moment? | VAS | yes/yes | VAS |
| ESRBTR | Patient´s evaluation of disease activity on a VAS ranging from “no activity” to “very active”. | VAS | Patient´s evaluation of pain on a VAS ranging from “no pain” to “strong pain”. | VAS | - | - | yes/no | NRS |
| ROBFIN | How are you doing regarding your joint disease during last week? | VAS | How much pain have you been experiencing during last week? | VAS | How much fatigue have you been experiencing during last week? | VAS | yes/yes | VAS |
| ICEBIO | Put a mark on the line below which illustrates the disease activity on your health due to your disease during the last week | VAS | Put a mark on the line below which illustrates the pain due to your disease during the last week | VAS | Put a mark on the line below which illustrates the fatigue due to your disease during the last week | VAS | yes/yes | VAS |
| GISEA | Considering all the ways your arthritis has affected you, how active do you feel your arthritis is today on a scale ranging from 0 to 100? | NRS 0-100 | Numerical rating scale ranging from 0 (no pain) to 100 (worst imaginable pain) measuring actual pain intensity during the last 24 hours | NRS 0-100 | - | - | yes/yes | VAS |
| NOR-DMARD | We kindly ask you to evaluate the activity in your arthritis during the last week. Considering all the symptoms you have had, how would you evaluate your condition? | VAS (from "good, no symptoms" to "very bad") | How much pain have you had during the last week? | VAS (from "no pain" to "unbearable pain") | To what degree has a feeling of unusual tiredness or exhaustion been a problem for you during the last week? | VAS (from "fatigue is no problem” to "fatigue is a big problem") | yes/no | VAS |
| Reuma.pt | Considering the way the disease disturbs you, how did you feel during the last week? | NRS and VAS | Please indicate the level of pain that you felt in your spine at any moment (day or night) during last week | VAS | FACIT questionnaire | - | yes/yes | VAS |
| RRBR | Please rate how much the disease is globally affecting you, taking into account all the aspects of the disease (e.g. psoriasis and arthritis) over the past week | NRS | Please rate the level of your joint pain related to PsA during the last week | NRS | *** | - | no/no |  |
| Biorx.si | How does your disease affect you today? | NRS | How severe was the pain in the past week? | NRS | - | - | yes/yes | VAS |
| BIOBADASER | No specific wording, depending on each center | NRS | - |  | - | - | yes/no | VAS |
| SRQ | How have you felt in the last week, in general, given your rheumatic disease? | VAS | How much pain have you had in the last week due to your rheumatic disease? | VAS | How tired have you been in the last week due to your rheumatic disease? | VAS | yes/yes | VAS |
| SCQM | How active is your disease today? | NRS | How would you rate your overall pain in the past 7 days? | NRS | *** | - | yes/yes | VAS |

NRS: numeric rating scale from 0 to 10 unless stated otherwise; VAS: visual analogue scale from 0 to 100 millimeter; BASDAI: bath ankylosing spondylitis disease activity index; BASFI: bath ankylosing spondylitis functional index.

*The wording is based on a translation from the original language (if not English) in the online survey and follow-up interviews; **Scales are evaluated visually using graphic presentation of the respective patient assessments in secondary anonymized data on patients with a clinical diagnosis of axial spondyloarthritis, 18 years or older, followed in one of the participating registries since the start of their first course of biological disease modifying anti-rheumatic drug (bDMARD) or targeted synthetic (ts)DMARD therapy between 2010 to 2021. Data from the first b/tsDMARD treatment course were used for this study. SRQ has provided data on Secukinumab treated patients only; ***BASDAI pain question; ****BASDAI fatigue question.
