# Supplementary material for: Commonalities and differences in set-up and data collection across European spondyloarthritis registries — results from the EuroSpA collaboration
Source: Arthritis Res Ther. 2023 Oct 19;25:205. doi: 10.1186/s13075-023-03184-7 (PMC10585911; doi:10.1186/s13075-023-03184-7)
Supplement: Supplementary file 3 — Additional file 3. Supplementary data: CRediT statement. [file 13075_2023_3184_MOESM3_ESM.docx]

| **Supplementary data: CRediT statement** |
| --- |

Author contributions, as defined by CRediT (Contributor Roles Taxonomy; <https://www.elsevier.com/authors/policies-and-guidelines/credit-author-statement>)

| **Author** | **Conceptualisation** | **Methodology** | **Software** | **Validation** | **Formal analysis** | **Investigation** | **Resources** | **Data curation** | **Writing**  **(Original draft)** | **Writing**  **(Review and editing)** | **Visualisation** | **Supervision** | **Project administration** | **Funding acquisition** |
| --- | --- | --- | --- | --- | --- | --- | --- | --- | --- | --- | --- | --- | --- | --- |
| Louise Linde | ✓ | ✓ | ✓ | ✓ | ✓ | ✓ |  | ✓ | ✓ | ✓ | ✓ |  | ✓ | ✓ |
| Lykke Midtbøll Ørnbjerg | ✓ | ✓ | ✓ |  | ✓ | ✓ |  | ✓ |  | ✓ |  |  |  | ✓ |
| Simon H Rasmussen | ✓ | ✓ | ✓ |  | ✓ | ✓ |  | ✓ |  | ✓ | ✓ |  |  | ✓ |
| Torvardur Jon Love | ✓ | ✓ | ✓ |  |  | ✓ |  |  |  | ✓ |  |  |  |  |
| Anne G Loft | ✓ | ✓ |  |  |  | ✓ | ✓ |  |  | ✓ |  |  |  |  |
| Jakub Zavada |  |  |  |  |  | ✓ | ✓ |  |  | ✓ |  |  |  |  |
| Jiri Vencovsky |  |  |  |  |  | ✓ | ✓ |  |  | ✓ |  |  |  |  |
| Karin Laas |  |  |  |  |  | ✓ | ✓ |  |  | ✓ |  |  |  |  |
| Dan Nordstrøm |  |  |  |  |  | ✓ | ✓ |  |  | ✓ |  |  |  |  |
| Tuulikki Sokka-Isler |  |  |  |  |  | ✓ | ✓ |  |  | ✓ |  |  |  |  |
| Bjorn Gudbjornsson |  |  |  |  |  | ✓ | ✓ |  |  | ✓ |  |  |  |  |
| Gerdur Gröndal |  |  |  |  |  | ✓ | ✓ |  |  | ✓ |  |  |  |  |
| Florenzo Iannone |  |  |  |  |  | ✓ | ✓ |  |  | ✓ |  |  |  |  |
| Roberta Ramonda |  |  |  |  |  | ✓ | ✓ |  |  | ✓ |  |  |  |  |
| Pasoon Hellmand |  |  |  |  |  | ✓ | ✓ |  |  | ✓ |  |  |  |  |
| Eirik K Kristianslund |  |  |  |  |  | ✓ | ✓ |  |  | ✓ |  |  |  |  |
| Tore K Kvien |  |  |  |  |  | ✓ | ✓ |  |  | ✓ |  |  |  |  |
| Ana M Rodrigues |  |  |  |  |  | ✓ | ✓ |  |  | ✓ |  |  |  |  |
| Maria J Santos |  |  |  |  |  | ✓ | ✓ |  |  | ✓ |  |  |  |  |
| Catalin Codreanu |  |  |  |  |  | ✓ | ✓ |  |  | ✓ |  |  |  |  |
| Ziga Rotar |  |  |  |  |  | ✓ | ✓ |  |  | ✓ |  |  |  |  |
| Matija Tomsic |  |  |  |  |  | ✓ | ✓ |  |  | ✓ |  |  |  |  |
| Isabel Castrejón |  |  |  |  |  | ✓ | ✓ |  |  | ✓ |  |  |  |  |
| Federico Díaz-González |  |  |  |  |  | ✓ | ✓ |  |  | ✓ |  |  |  |  |
| Daniela Di Giuseppe |  |  |  |  |  | ✓ | ✓ |  |  | ✓ |  |  |  |  |
| Lotta Ljung |  |  |  |  |  | ✓ | ✓ |  |  | ✓ |  |  |  |  |
| Michael J Nissen |  |  |  |  |  | ✓ | ✓ |  |  | ✓ |  |  |  |  |
| Adrian Ciurea |  |  |  |  |  | ✓ | ✓ |  |  | ✓ |  |  |  |  |
| Gary J Macfarlane |  |  |  |  |  | ✓ | ✓ |  |  | ✓ |  |  |  |  |
| Maureen Heddle |  |  |  |  |  | ✓ | ✓ |  |  | ✓ |  |  |  |  |
| Bente Glintborg |  |  |  |  |  | ✓ | ✓ |  |  | ✓ |  |  |  |  |
| Mikkel Østergaard | ✓ | ✓ |  |  |  | ✓ | ✓ |  |  | ✓ |  | ✓ | ✓ | ✓ |
| Merete Lund Hetland | ✓ | ✓ |  |  |  | ✓ | ✓ |  |  | ✓ |  | ✓ | ✓ | ✓ |

| **Term** | **Definition** |  |
| --- | --- | --- |
| Conceptualisation | Ideas; formulation or evolution of overarching research goals and aims | |
| Methodology | Development or design of methodology; creation of models | |
| Software | Programming, software development; designing computer programs; implementation of the computer code and supporting algorithms; testing of existing code components | |
| Validation | Verification, whether as a part of the activity or separate, of the overall replication/ reproducibility of results/experiments and other research outputs | |
| Formal analysis | Application of statistical, mathematical, computational, or other formal techniques to analyse or synthesize study data | |
| Investigation | Conducting a research and investigation process, specifically performing the experiments, or data/evidence collection | |
| Resources | Provision of study materials, reagents, materials, patients, laboratory samples, animals, instrumentation, computing resources, or other analysis tools | |
| Data curation | Management activities to annotate (produce metadata), scrub data and maintain research data (including software code, where it is necessary for interpreting the data itself) for initial use and later reuse | |
| Writing (Original draft) | Preparation, creation and/or presentation of the published work, specifically writing the initial draft (including substantive translation) | |
| Writing (Review and editing) | Preparation, creation and/or presentation of the published work by those from the original research group, specifically critical review, commentary or revision – including pre- or post-publication stages | |
| Visualisation | Preparation, creation and/or presentation of the published work, specifically visualization/ data presentation | |
| Supervision | Oversight and leadership responsibility for the research activity planning and execution, including mentorship external to the core team | |
| Project administration | Management and coordination responsibility for the research activity planning and execution | |
| Funding acquisition | Acquisition of the financial support for the project leading to this publication | |
